# Supplementary material for: Multicomponent Domino Cyclization of Ethyl Trifluoropyruvate with Methyl Ketones and Amino Alcohols as A New Way to γ-Lactam Annulated Oxazacycles
Source: Molecules. 2023 Feb 20;28(4):1983. doi: 10.3390/molecules28041983 (PMC9967980; doi:10.3390/molecules28041983)

checkCIF/PLATON report

Structure factors have been supplied for datablock(s) dc

THIS REPORT IS FOR GUIDANCE ONLY. IF USED AS PART OF A REVIEW PROCEDURE FOR PUBLICATION, IT SHOULD NOT REPLACE THE EXPERTISE OF AN EXPERIENCED CRYSTALLOGRAPHIC REFEREE.

No syntax errors found. CIF dictionary Interpreting this report

Datablock: dc

```
Bond precision:   C-C = 0.0076 A          Wavelength=0.71073

Cell:             a=19.1431(10)     b=6.4882(4)         c=16.4430(9)
                  alpha=90          beta=107.551(6)     gamma=90
Temperature:      295 K

                 Calculated                    Reported
Volume           1947.2(2)                     1947.2(2)
Space group      P 21/c                        P 1 21/c 1
Hall group       -P 2ybc                       -P 2ybc
Moiety formula   C15 H19 F6 N O6               C15 H19 F6 N O6
Sum formula      C15 H19 F6 N O6               C15 H19 F6 N O6
Mr               423.31                        423.31
Dx,g cm-3        1.444                         1.444
Z                4                             4
Mu (mm-1)        0.145                         0.145
F000             872.0                         872.0
F000'            872.78
h,k,lmax         23,8,20                       23,8,20
Nref             3974                          3972
Tmin,Tmax        0.986,0.999                   0.424,1.000
Tmin'            0.969

Correction method= # Reported T Limits: Tmin=0.424 Tmax=1.000
AbsCorr = MULTI-SCAN

Data completeness= 0.999           Theta(max)= 26.371

R(reflections)= 0.0601( 1265)                           wR2(reflections)=
                                                        0.2534( 3972)
S = 1.015                    Npar= 270
```

The following ALERTS were generated. Each ALERT has the format
test-name_ALERT_alert-type_alert-level.
Click on the hyperlinks for more details of the test.

Alert level B

```
PLAT026_ALERT_3_B Ratio Observed / Unique Reflections (too) Low ..        32% Check
PLAT031_ALERT_4_B Refined Extinction Parameter Within Range of ...     2.389 Sigma
PLAT241_ALERT_2_B High   'MainMol' Ueq as Compared to Neighbors of       C12 Check
```

Alert level C

```
RINTA01_ALERT_3_C  The value of Rint is greater than 0.12
            Rint given   0.153
PLAT220_ALERT_2_C NonSolvent   Resd 1  C   Ueq(max)/Ueq(min) Range        3.3 Ratio
PLAT242_ALERT_2_C Low    'MainMol' Ueq as Compared to Neighbors of         C8 Check
PLAT260_ALERT_2_C Large Average Ueq of Residue Including       F1      0.102 Check
PLAT340_ALERT_3_C Low Bond Precision on  C-C Bonds ...............    0.00764 Ang.
PLAT905_ALERT_3_C Negative K value in the Analysis of Variance ...   -169.676 Report
PLAT905_ALERT_3_C Negative K value in the Analysis of Variance ...     -4.068 Report
PLAT905_ALERT_3_C Negative K value in the Analysis of Variance ...    -27.777 Report
PLAT905_ALERT_3_C Negative K value in the Analysis of Variance ...     -2.944 Report
PLAT905_ALERT_3_C Negative K value in the Analysis of Variance ...     -7.424 Report
PLAT905_ALERT_3_C Negative K value in the Analysis of Variance ...     -1.329 Report
PLAT905_ALERT_3_C Negative K value in the Analysis of Variance ...     -2.939 Report
PLAT905_ALERT_3_C Negative K value in the Analysis of Variance ...     -0.249 Report
PLAT905_ALERT_3_C Negative K value in the Analysis of Variance ...     -0.789 Report
```

Alert level G

```
PLAT003_ALERT_2_G Number of Uiso or Uij Restrained non-H Atoms ...          2 Report
PLAT005_ALERT_5_G No Embedded Refinement Details Found  in the CIF     Please Do !
PLAT020_ALERT_3_G The Value of Rint is Greater Than 0.12 .........      0.153 Report
PLAT230_ALERT_2_G Hirshfeld Test Diff for    C11      --C12      .        5.8 s.u.
PLAT230_ALERT_2_G Hirshfeld Test Diff for    C11A     --C12      .       10.4 s.u.
PLAT242_ALERT_2_G Low    'MainMol' Ueq as Compared to Neighbors of         C1 Check
PLAT242_ALERT_2_G Low    'MainMol' Ueq as Compared to Neighbors of         C9 Check
PLAT300_ALERT_4_G Atom Site Occupancy of C11        Constrained at        0.5 Check
PLAT300_ALERT_4_G Atom Site Occupancy of C11A       Constrained at        0.5 Check
PLAT300_ALERT_4_G Atom Site Occupancy of H11A       Constrained at        0.5 Check
PLAT300_ALERT_4_G Atom Site Occupancy of H11B       Constrained at        0.5 Check
PLAT300_ALERT_4_G Atom Site Occupancy of H11C       Constrained at        0.5 Check
PLAT300_ALERT_4_G Atom Site Occupancy of H11D       Constrained at        0.5 Check
PLAT300_ALERT_4_G Atom Site Occupancy of H12A       Constrained at        0.5 Check
PLAT300_ALERT_4_G Atom Site Occupancy of H12B       Constrained at        0.5 Check
PLAT300_ALERT_4_G Atom Site Occupancy of H12C       Constrained at        0.5 Check
PLAT300_ALERT_4_G Atom Site Occupancy of H12D       Constrained at        0.5 Check
PLAT300_ALERT_4_G Atom Site Occupancy of H12E       Constrained at        0.5 Check
PLAT300_ALERT_4_G Atom Site Occupancy of H12F       Constrained at        0.5 Check
PLAT301_ALERT_3_G Main Residue  Disorder .............(Resd  1  )         4% Note
PLAT793_ALERT_4_G Model has Chirality at C3          (Centro SPGR)          S Verify
PLAT793_ALERT_4_G Model has Chirality at C8          (Centro SPGR)          R Verify
PLAT860_ALERT_3_G Number of Least-Squares Restraints ............          12 Note
PLAT910_ALERT_3_G Missing # of FCF Reflection(s) Below Theta(Min).          2 Note
PLAT978_ALERT_2_G Number C-C Bonds with Positive Residual Density.          1 Info
```

```
PLAT992_ALERT_5_G Repd & Actual _reflns_number_gt Values Differ by          2 Check
```

```
   0 ALERT level A = Most likely a serious problem - resolve or explain
   3 ALERT level B = A potentially serious problem, consider carefully
  14 ALERT level C = Check. Ensure it is not caused by an omission or oversight
  26 ALERT level G = General information/check it is not something unexpected

   0 ALERT type 1 CIF construction/syntax error, inconsistent or missing data
  10 ALERT type 2 Indicator that the structure model may be wrong or deficient
  16 ALERT type 3 Indicator that the structure quality may be low
  15 ALERT type 4 Improvement, methodology, query or suggestion
   2 ALERT type 5 Informative message, check
```

It is advisable to attempt to resolve as many as possible of the alerts in all categories. Often the minor alerts point to easily fixed oversights, errors and omissions in your CIF or refinement strategy, so attention to these fine details can be worthwhile. In order to resolve some of the more serious problems it may be necessary to carry out additional measurements or structure refinements. However, the purpose of your study may justify the reported deviations and the more serious of these should normally be commented upon in the discussion or experimental section of a paper or in the "special_details" fields of the CIF. checkCIF was carefully designed to identify outliers and unusual parameters, but every test has its limitations and alerts that are not important in a particular case may appear. Conversely, the absence of alerts does not guarantee there are no aspects of the results needing attention. It is up to the individual to critically assess their own results and, if necessary, seek expert advice.

Publication of your CIF in IUCr journals

A basic structural check has been run on your CIF. These basic checks will be run on all CIFs submitted for publication in IUCr journals (*Acta Crystallographica*, *Journal of Applied Crystallography*, *Journal of Synchrotron Radiation*); however, if you intend to submit to *Acta Crystallographica Section C* or *E* or *IUCrData*, you should make sure that full publication checks are run on the final version of your CIF prior to submission.

Publication of your CIF in other journals

Please refer to the *Notes for Authors* of the relevant journal for any special instructions relating to CIF submission.

PLATON version of 28/11/2022; check.def file version of 28/11/2022

Datablock dc - ellipsoid plot

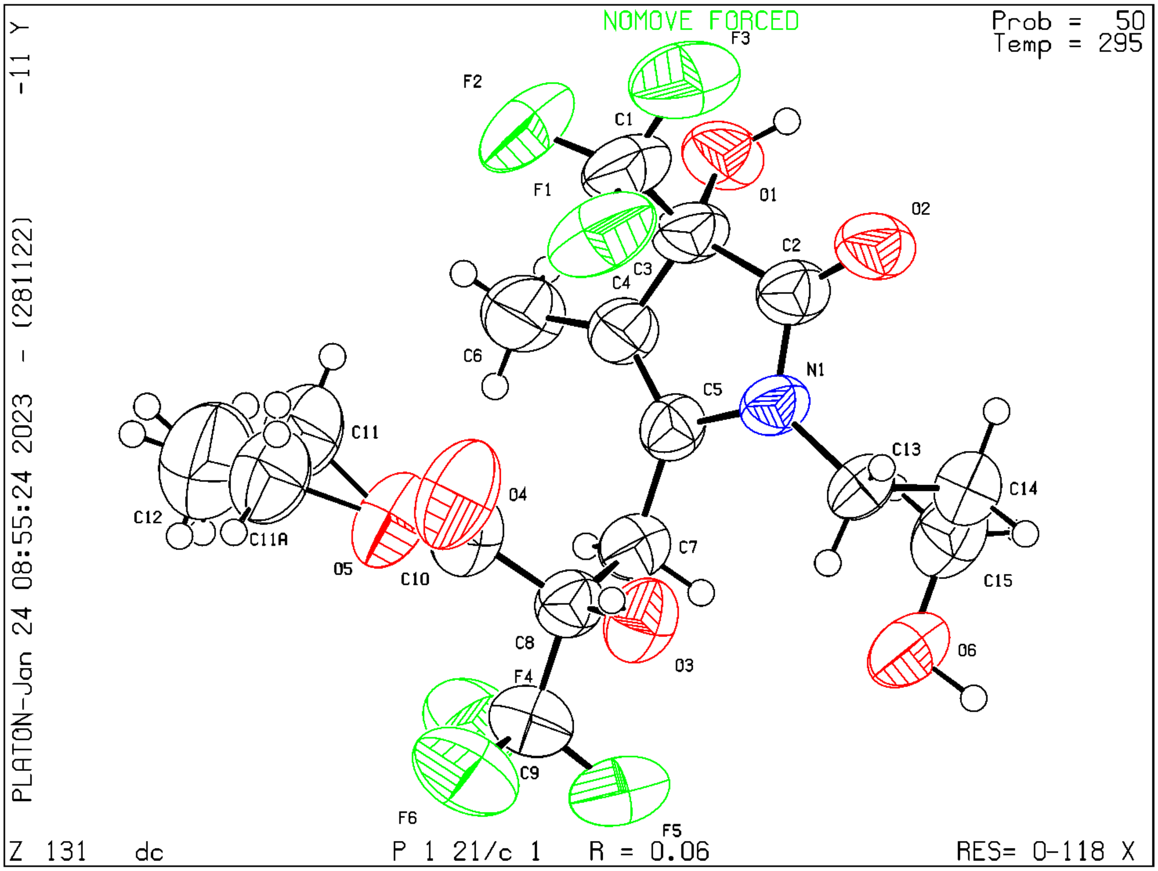

Supplement: Supplementary file 1 [file molecules-28-01983-s001.zip › Supporting informations/checkcif 11b.pdf]
