# Supplementary material for: Multicomponent Domino Cyclization of Ethyl Trifluoropyruvate with Methyl Ketones and Amino Alcohols as A New Way to γ-Lactam Annulated Oxazacycles
Source: Molecules. 2023 Feb 20;28(4):1983. doi: 10.3390/molecules28041983 (PMC9967980; doi:10.3390/molecules28041983)

checkCIF/PLATON report

Structure factors have been supplied for datablock(s) exp_982

THIS REPORT IS FOR GUIDANCE ONLY. IF USED AS PART OF A REVIEW PROCEDURE FOR PUBLICATION, IT SHOULD NOT REPLACE THE EXPERTISE OF AN EXPERIENCED CRYSTALLOGRAPHIC REFEREE.

No syntax errors found. CIF dictionary Interpreting this report

Datablock: exp_982

Bond precision: C-C = 0.0049 A Wavelength=0.71073

Cell:	a=10.6332(12)	b=13.8923(13)	c=9.5664(15)
	alpha=90	beta=102.235(13)	gamma=90
Temperature:	295 K		

	Calculated	Reported
Volume	1381.1(3)	1381.0(3)
Space group	P 21/c	P 1 21/c 1
Hall group	-P 2ybc	-P 2ybc
Moiety formula	C12 H18 F3 N O3	C12 H18 F3 N O3
Sum formula	C12 H18 F3 N O3	C12 H18 F3 N O3
Mr	281.27	281.27
Dx,g cm-3	1.353	1.353
Z	4	4
Mu (mm-1)	0.122	0.122
F000	592.0	592.0
F000'	592.44	
h,k,lmax	15,20,13	14,19,12
Nref	4343	3779
Tmin,Tmax	0.949,0.966	0.767,1.000
Tmin'	0.948	

Correction method= # Reported T Limits: Tmin=0.767 Tmax=1.000
AbsCorr = MULTI-SCAN

Data completeness= 0.870 Theta(max)= 30.861

R(reflections)= 0.0611(1481) wR2(reflections)= 0.2201(3779)

S = 0.980 Npar= 210

The following ALERTS were generated. Each ALERT has the format
test-name_ALERT_alert-type_alert-level.
Click on the hyperlinks for more details of the test.

Alert level B

```
PLAT026_ALERT_3_B Ratio Observed / Unique Reflections (too) Low ..        39% Check
```

Alert level C

```
PLAT031_ALERT_4_C Refined Extinction Parameter Within Range of ...      2.667 Sigma
PLAT220_ALERT_2_C NonSolvent   Resd 1  C   Ueq(max)/Ueq(min) Range        3.1 Ratio
PLAT230_ALERT_2_C Hirshfeld Test Diff for    C11       --C12      .       5.6 s.u.
PLAT340_ALERT_3_C Low Bond Precision on  C-C Bonds ..............      0.0049 Ang.
PLAT360_ALERT_2_C Short  C(sp3)-C(sp3) Bond  C12       - C13      .      1.43 Ang.
PLAT905_ALERT_3_C Negative K value in the Analysis of Variance ...    -11.313 Report
PLAT905_ALERT_3_C Negative K value in the Analysis of Variance ...     -0.073 Report
PLAT905_ALERT_3_C Negative K value in the Analysis of Variance ...     -1.947 Report
PLAT910_ALERT_3_C Missing # of FCF Reflection(s) Below Theta(Min).          5 Note
PLAT911_ALERT_3_C Missing FCF Refl Between Thmin & STh/L=    0.600         17 Report
```

Alert level G

```
PLAT012_ALERT_1_G No       _shelx_res_checksum Found in CIF ......     Please Check
PLAT242_ALERT_2_G Low    'MainMol' Ueq as Compared to Neighbors of        C14 Check
PLAT793_ALERT_4_G Model has Chirality at C7          (Centro SPGR)          R Verify
PLAT793_ALERT_4_G Model has Chirality at C9          (Centro SPGR)          S Verify
PLAT912_ALERT_4_G Missing # of FCF Reflections Above STh/L=  0.600        474 Note
PLAT933_ALERT_2_G Number of HKL-OMIT Records in Embedded .res File          4 Note
PLAT978_ALERT_2_G Number C-C Bonds with Positive Residual Density.          0 Info
```

0 **ALERT level A** = Most likely a serious problem - resolve or explain
1 **ALERT level B** = A potentially serious problem, consider carefully
10 **ALERT level C** = Check. Ensure it is not caused by an omission or oversight
7 **ALERT level G** = General information/check it is not something unexpected

1 ALERT type 1 CIF construction/syntax error, inconsistent or missing data
6 ALERT type 2 Indicator that the structure model may be wrong or deficient
7 ALERT type 3 Indicator that the structure quality may be low
4 ALERT type 4 Improvement, methodology, query or suggestion
0 ALERT type 5 Informative message, check

It is advisable to attempt to resolve as many as possible of the alerts in all categories. Often the minor alerts point to easily fixed oversights, errors and omissions in your CIF or refinement strategy, so attention to these fine details can be worthwhile. In order to resolve some of the more serious problems it may be necessary to carry out additional measurements or structure refinements. However, the purpose of your study may justify the reported deviations and the more serious of these should normally be commented upon in the discussion or experimental section of a paper or in the "special_details" fields of the CIF. checkCIF was carefully designed to identify outliers and unusual parameters, but every test has its limitations and alerts that are not important in a particular case may appear. Conversely, the absence of alerts does not guarantee there are no aspects of the results needing attention. It is up to the individual to critically assess their own results and, if necessary, seek expert advice.

Publication of your CIF in IUCr journals

A basic structural check has been run on your CIF. These basic checks will be run on all CIFs submitted for publication in IUCr journals (*Acta Crystallographica*, *Journal of Applied Crystallography*, *Journal of Synchrotron Radiation*); however, if you intend to submit to *Acta Crystallographica Section C* or *E* or *IUCrData*, you should make sure that full publication checks are run on the final version of your CIF prior to submission.

Publication of your CIF in other journals

Please refer to the *Notes for Authors* of the relevant journal for any special instructions relating to CIF submission.

PLATON version of 28/11/2022; check.def file version of 28/11/2022

Datablock exp_982 - ellipsoid plot

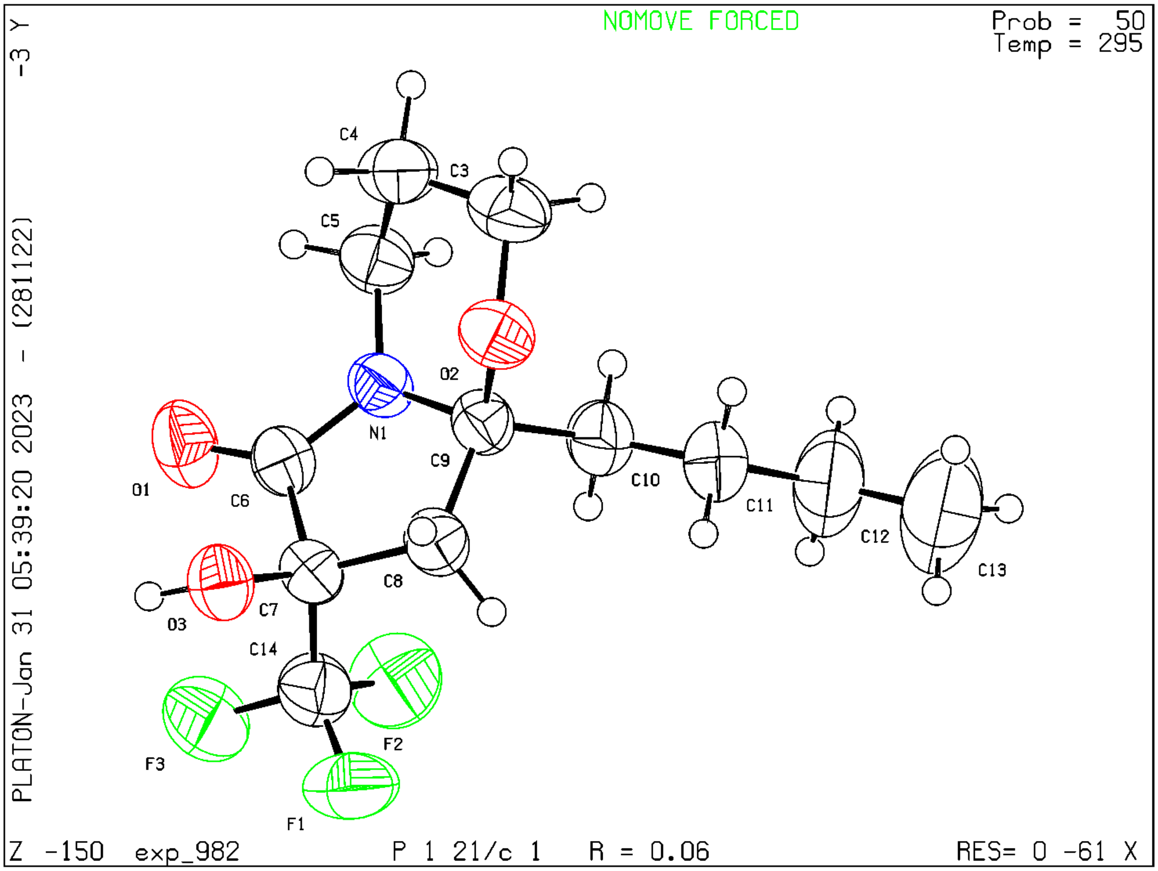

Supplement: Supplementary file 1 [file molecules-28-01983-s001.zip › Supporting informations/checkcif 8cc.pdf]
