# Supplementary material for: Multicomponent Domino Cyclization of Ethyl Trifluoropyruvate with Methyl Ketones and Amino Alcohols as A New Way to γ-Lactam Annulated Oxazacycles
Source: Molecules. 2023 Feb 20;28(4):1983. doi: 10.3390/molecules28041983 (PMC9967980; doi:10.3390/molecules28041983)
Supplement: Supplementary file 1 [file molecules-28-01983-s001.zip › Supporting informations/Supporting information.pdf]

Supporting Information

Multicomponent domino cyclization of ethyl trifluoropyruvate with methyl ketones and amino alcohols – a new way to γ–lactam annulated oxazacycles

Marina V. Goryaeva [1], Olesya A. Fefelova [1], Yanina V. Burgart [1], Marina A. Ezhikova [1], Mikhail I. Kodess [1], Pavel A. Slepukhin [1], Vasily S. Gaviko [2], Victor I. Saloutin [1,*]

[1]Postovsky Institute of Organic Synthesis of the Ural Branch of the Russian Academy of Science (IOS UB RAS), S. Kovalevskoi St., 22, Ekaterinburg 620108, Russia.

[2]M.N. Mikheev Institute of Metal Physics of the Ural Branch of the Russian Academy of Sciences (IMP UB RAS), S. Kovalevskoi St., 18, Ekaterinburg 620108, Russia.

*Correspondence: saloutin@ios.uran.ru

Contents

1. Crystallographic parameters and X-ray experiment X-ray details

   (Tables S1, S2)……………………………………………………………2

2. Copies of ^{1}H, ^{13}C and ^{19}F NMR spectra for all compounds

   (Figures S1-S65)……………………………………………………………4

Table S1. Crystallographic parameters and X-ray experiment details for **4d**[c], **5**[tc].

Compound	4d[c]	5[tc]
Empirical formula	$C_{13}H_{12}F_3NO_3$	$C_{13}H_{15}F_6NO_6$
Formula weight	287.24	395.26
Temperature/K	295(2)	295(2)
Crystal system	triclinic	monoclinic
Space group	P-1	$P2_1/n$
a/Å	6.0290(7)	10.8844(8)
b/Å	8.1542(10)	13.1919(9)
c/Å	13.4778(17)	11.3734(9)
α/°	79.481(10)	90
β/°	86.443(10)	92.641(7)
γ/°	75.029(10)	90
Volume/$Å^3$	629.27(14)	1631.3(2)
Z	2	4
$\rho_{calc}g/cm^3$	1.516	1.609
μ/mm^{-1}	0.136	0.167
F(000)	296.0	808.0
Crystal size/mm^3	0.41 × 0.29 × 0.17	0.45 × 0.29 × 0.13
Radiation	MoKα (λ = 0.71073)	MoKα (λ = 0.71073)
2Θ range for data collection/°	7.366 to 61.83	7.144 to 62.028
Index ranges	$-8 \le h \le 8, -11 \le k \le 10, -15 \le l \le 18$	$-15 \le h \le 13, -17 \le k \le 18, -16 \le l \le 15$
Reflections collected	5481	11283
Independent reflections	3339 [R_{int} = 0.0434, R_{sigma} = 0.0881]	4404 [R_{int} = 0.0496, R_{sigma} = 0.0604]
Data/restraints/parameters	3339/0/186	4404/0/245
Goodness-of-fit on F^2	1.015	1.003
Final R indexes [I>=2σ (I)]	R_1 = 0.0702, wR_2 = 0.1634	R_1 = 0.0544, wR_2 = 0.1455
Final R indexes [all data]	R_1 = 0.1428, wR_2 = 0.2335	R_1 = 0.1148, wR_2 = 0.2039
Largest diff. peak/hole / e $Å^{-3}$	0.26/-0.24	0.22/-0.21

Table S2. Crystallographic parameters and X-ray experiment details for **8c**[c], **11b.**

**Compound**	**8c**[c]	**11b**
Empirical formula	$C_{12}H_{18}F_3NO_3$	$C_{15}H_{19}NO_6F_6$
Formula weight	281.27	423.31
Temperature/K	295(2)	295(2)
Crystal system	monoclinic	monoclinic
Space group	$P2_1/c$	$P2_1/c$
a/Å	10.6332(12)	19.1431(10)
b/Å	13.8923(13)	6.4882(4)
c/Å	9.5664(15)	16.4430(9)
α/°	90	90
β/°	102.235(13)	107.551(6)
γ/°	90	90
Volume/$Å^3$	1381.0(3)	1947.2(2)
Z	4	4
$\rho_{calc}g/cm^3$	1.353	1.444
μ/mm^{-1}	0.122	0.145
F(000)	592.0	872.0
Crystal size/mm^3	0.44 × 0.36 × 0.28	0.22 × 0.08 × 0.01
Radiation	Mo Kα (λ = 0.71073)	MoKα (λ = 0.71073)
2Θ range for data collection/°	7.056 to 61.722	5 to 52.742
Index ranges	-14 ≤ h ≤ 14, -18 ≤ k ≤ 19, -12 ≤ l ≤ 11	-23 ≤ h ≤ 23, -8 ≤ k ≤ 8, -20 ≤ l ≤ 20
Reflections collected	10666	15118
Independent reflections	3779 [R_{int} = 0.0582, R_{sigma} = 0.0803]	3972 [R_{int} = 0.1526, R_{sigma} = 0.0998]
Data/restraints/parameters	3779/0/210	3972/12/270
Goodness-of-fit on F^2	0.980	1.015
Final R indexes [I>=2σ (I)]	R_1 = 0.0611, wR_2 = 0.1539	R_1 = 0.0601, wR_2 = 0.1547
Final R indexes [all data]	R_1 = 0.1610, wR_2 = 0.2201	R_1 = 0.2070, wR_2 = 0.2534
Largest diff. peak/hole / e $Å^{-3}$	0.30/-0.22	0.27/-0.28

Copies of ^{1}H, ^{13}C and ^{19}F NMR spectra for all compounds

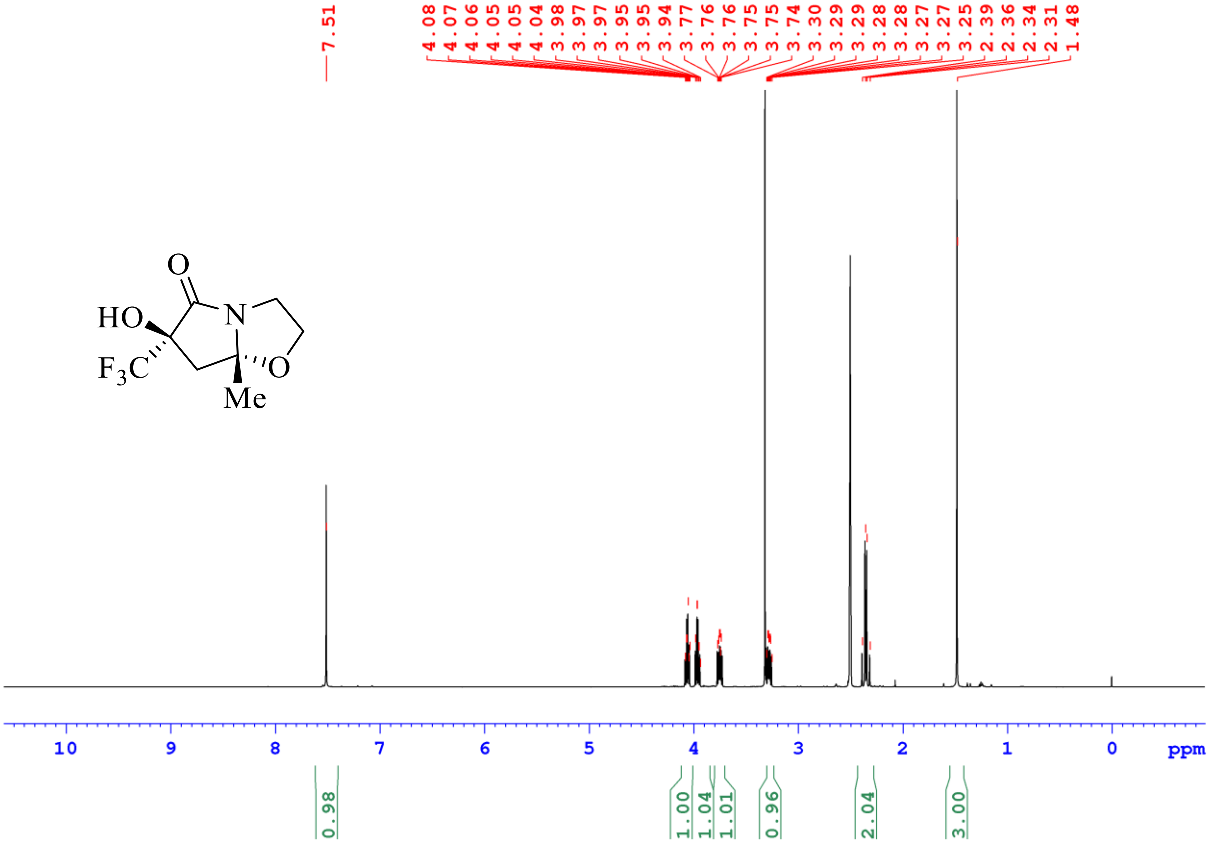

Figure S1. ^{1}H NMR spectrum of **4a^{t}.**

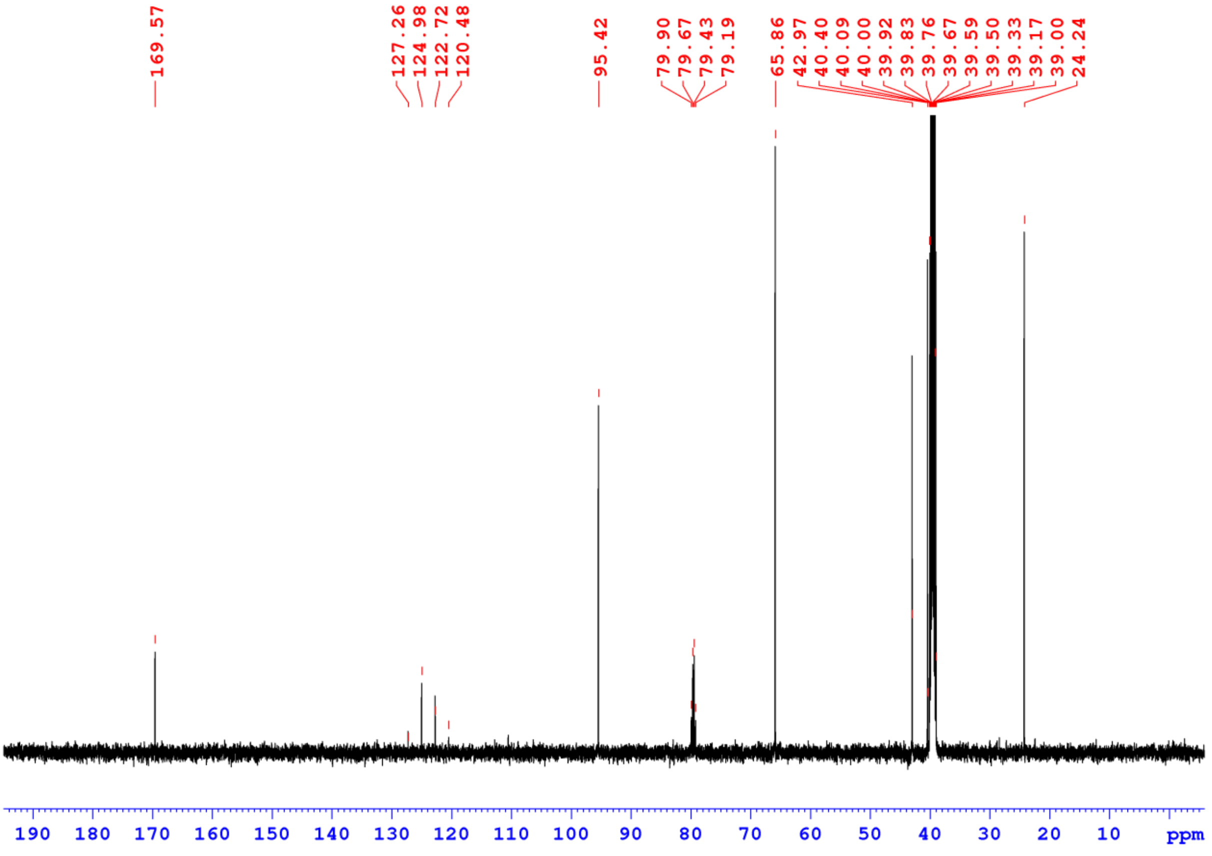

Figure S2. ^{13}C NMR spectrum of **4a^{t}.**

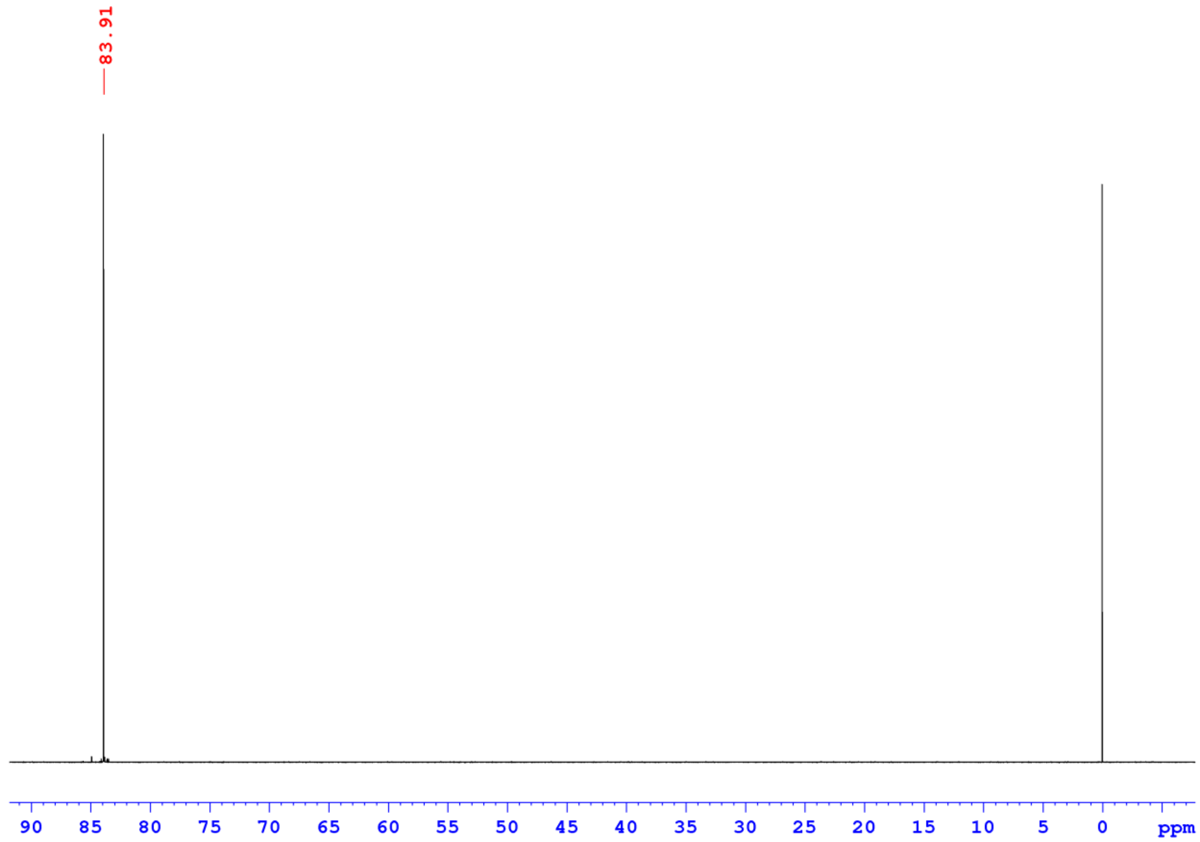

Figure S3. ^{19}F NMR spectrum of **4a^{t}**.

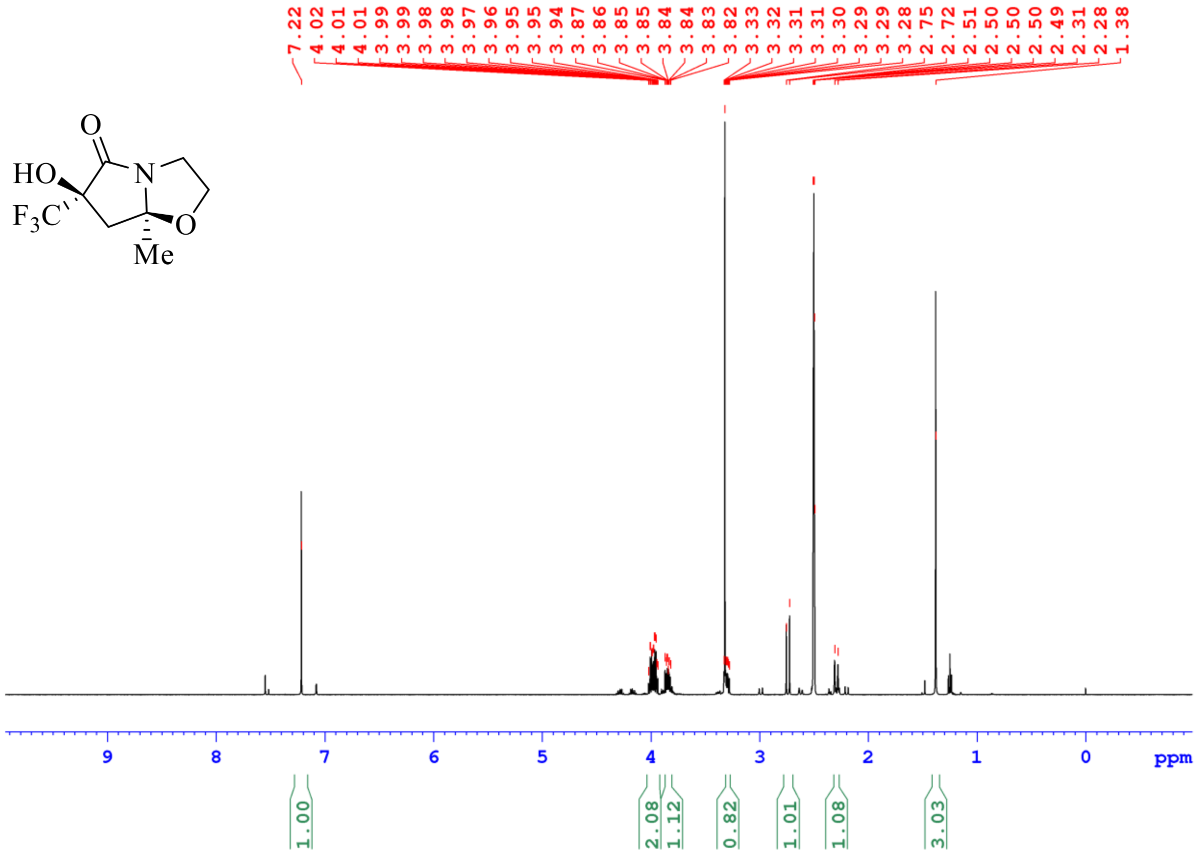

Figure S4. ^{1}H NMR spectrum of **4a^{c}** (mixed with **5^{ct}** (20%) and **4a^{t}** (2%)).

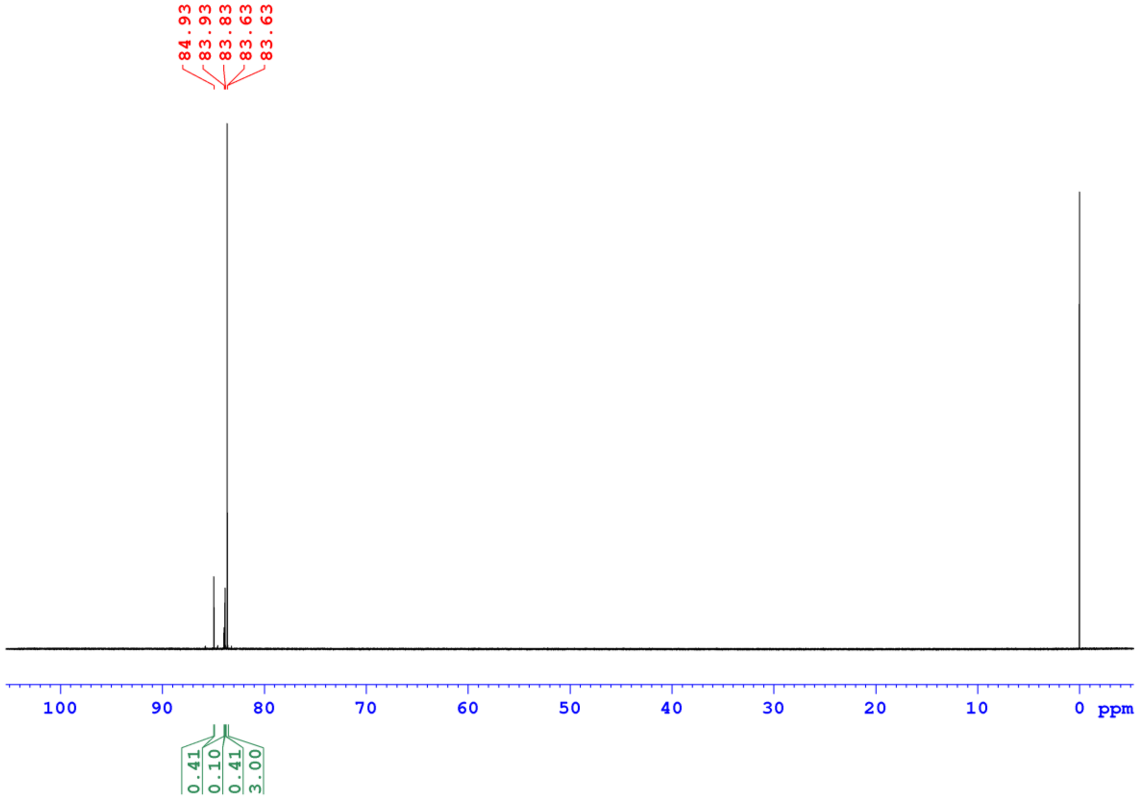

Figure S5. ^{19}F NMR spectrum of **4a**^{c} (mixed with **5**^{ct} (20%) and **4a**^{t} (2%)).

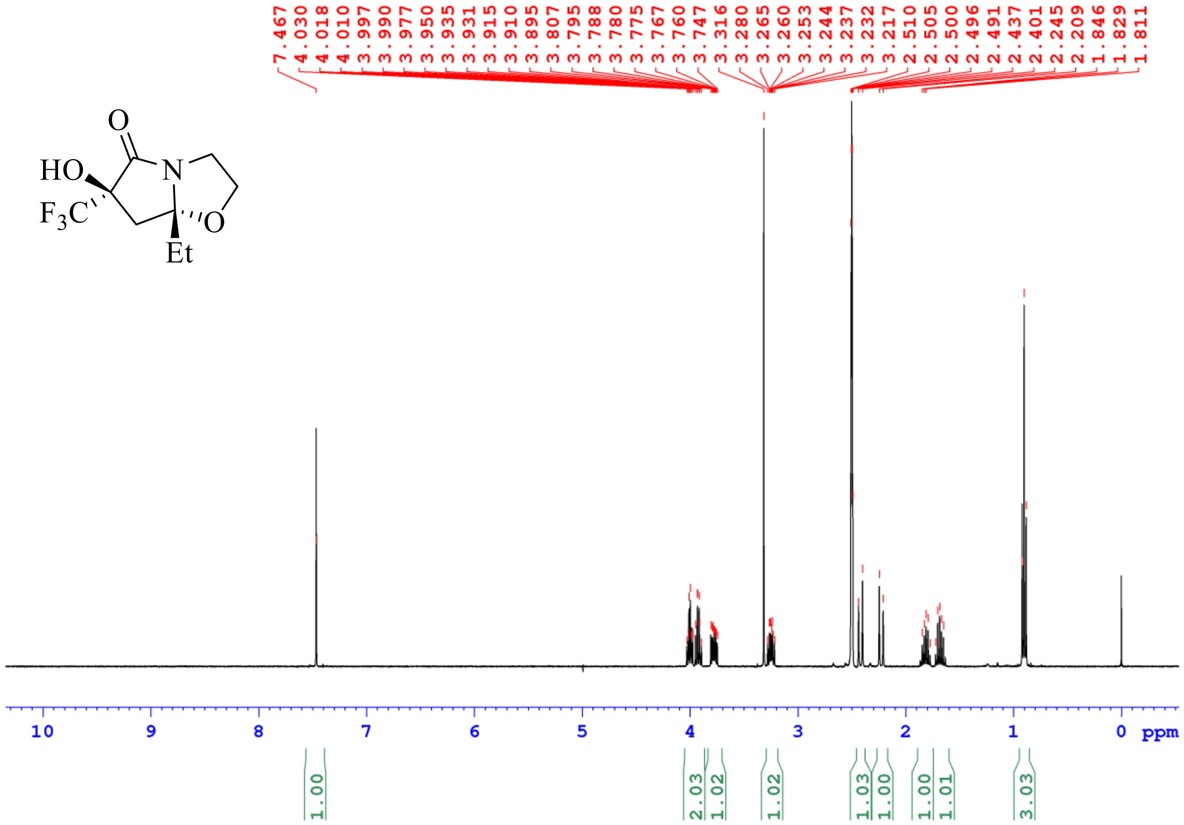

Figure S6. ^{1}H NMR spectrum of **4b**^{t}.

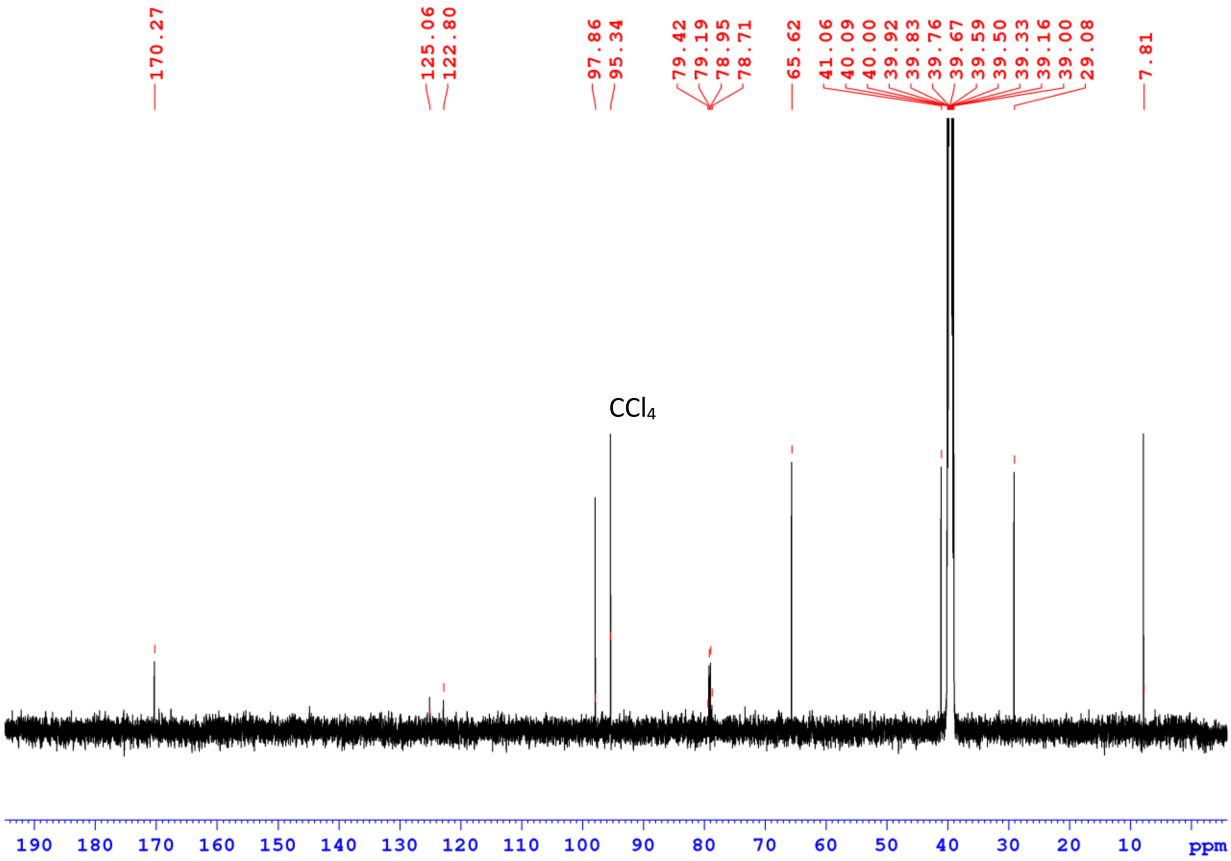

Figure S7. ^{1}H NMR spectrum of **4b^{t}**.

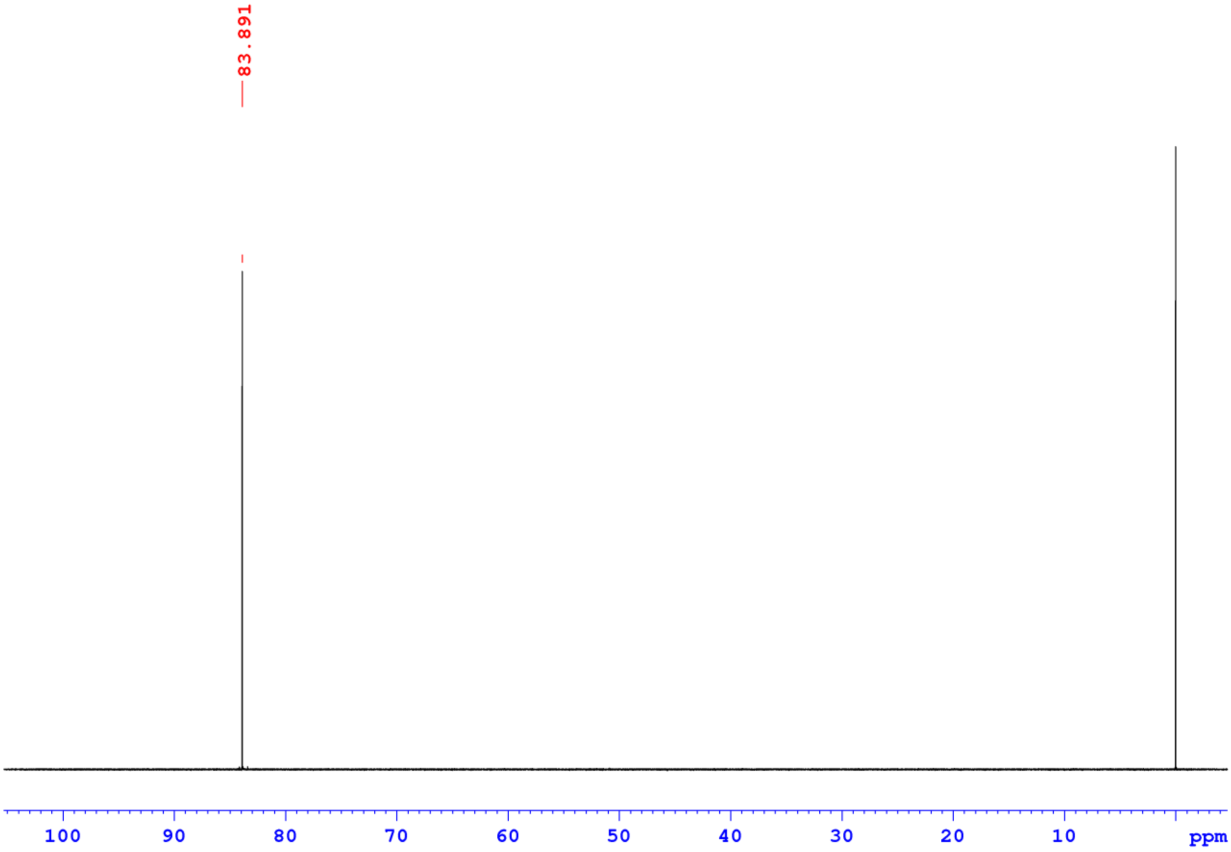

Figure S8. ^{19}F NMR spectrum of **4b^{t}**.

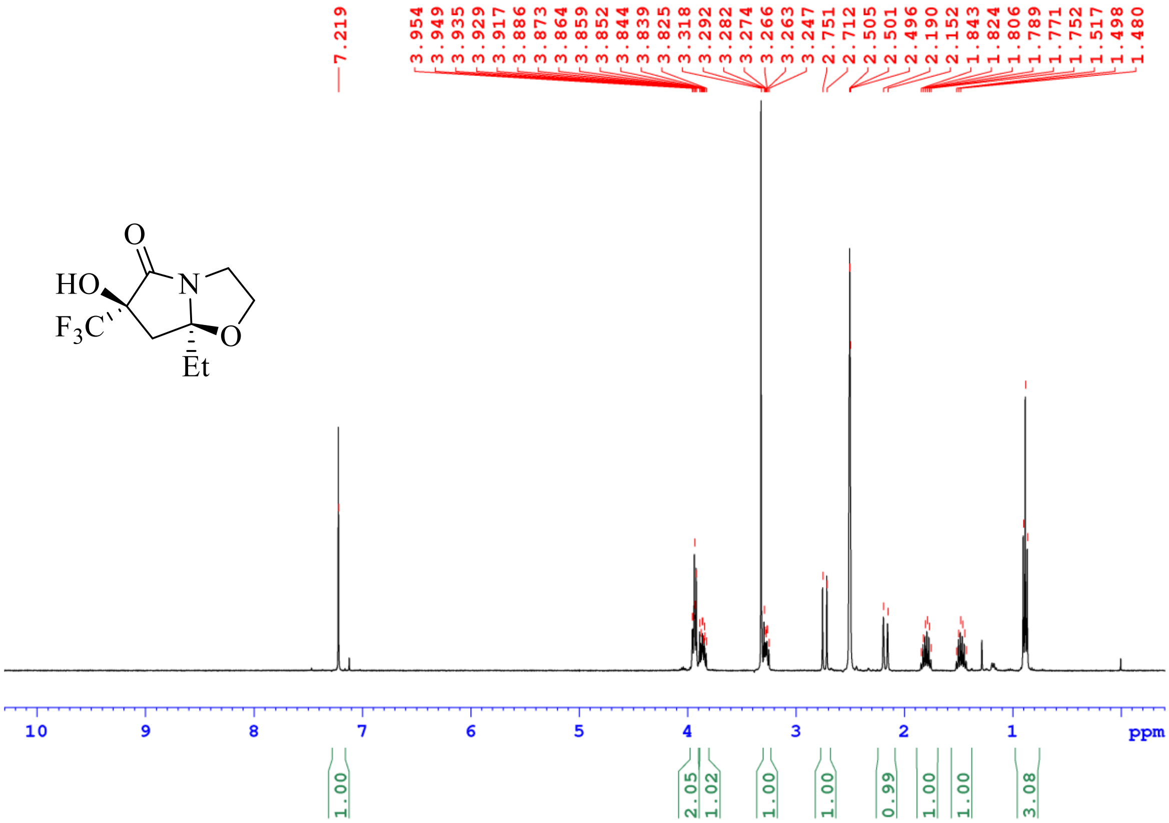

Figure S9. ^{1}H NMR spectrum of **4b^{c}** (mixed with **4b^{t}** in the ratio 90:10).

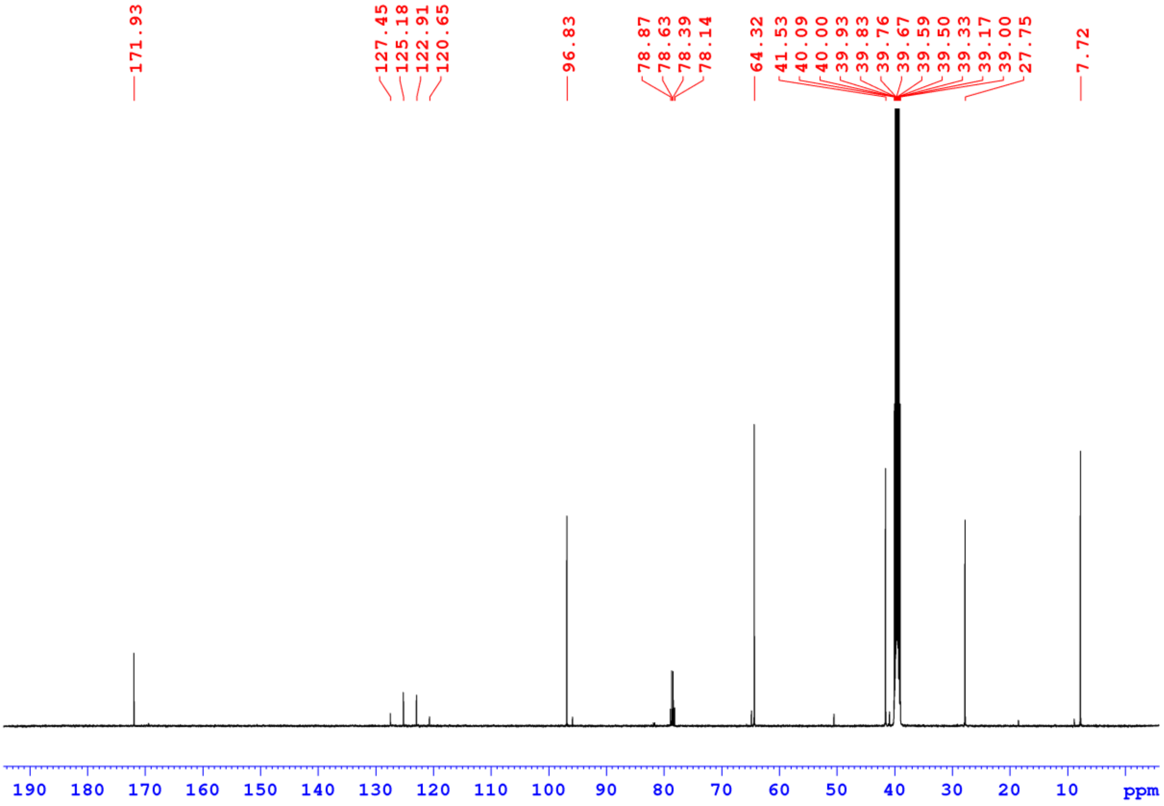

Figure S10. ^{13}C NMR spectrum of **4b^{c}** (mixed with **4b^{t}** in the ratio 90:10).

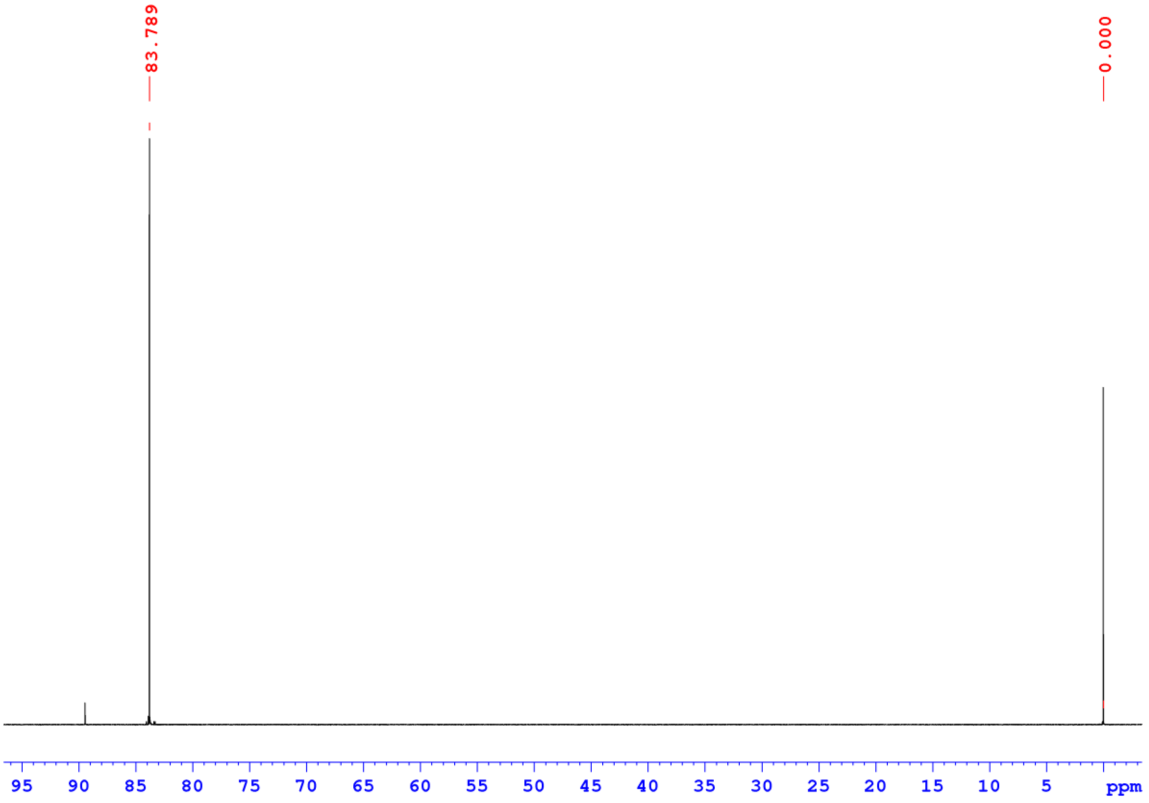

Figure S11. ^{19}F NMR spectrum of **4b^{c}** (mixed with **4b^{t}** in the ratio 90:10).

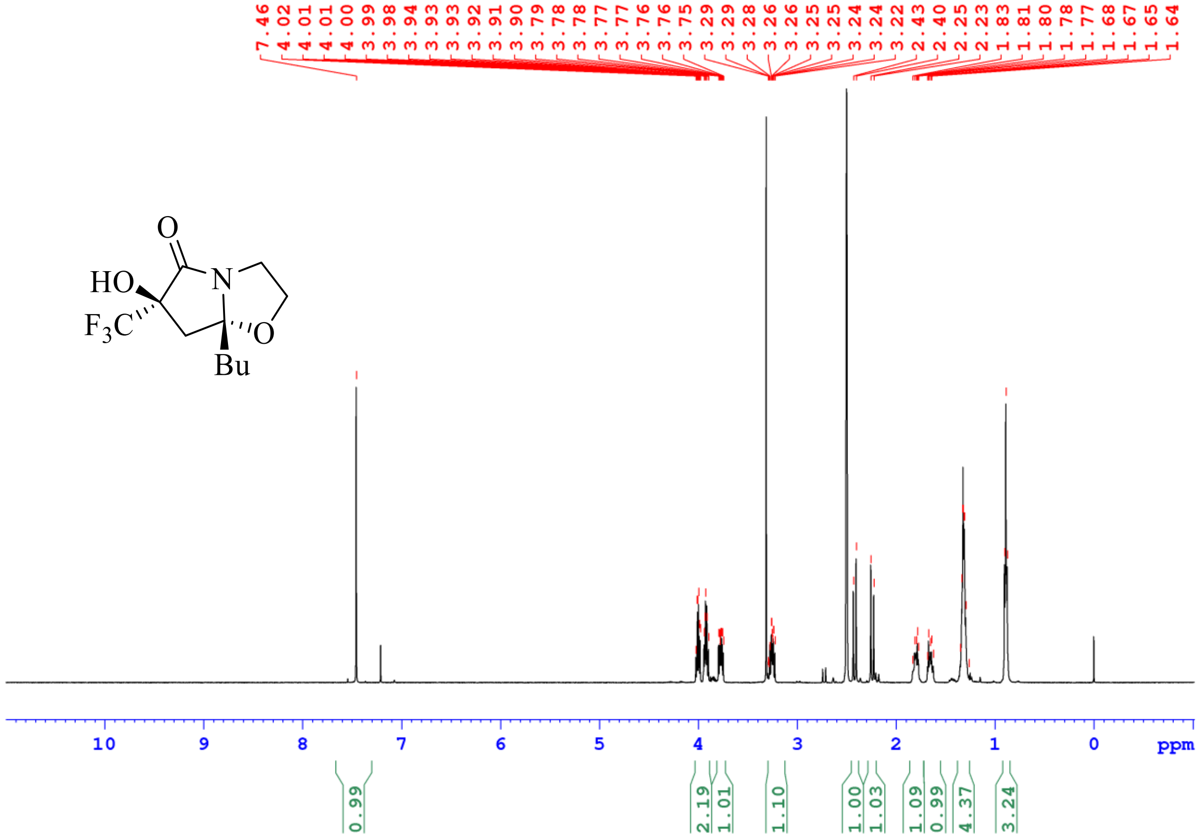

Figure S12. ^{1}H NMR spectrum of **4c^{t}** (mixed with **4c^{c}** in the ratio 90:10).

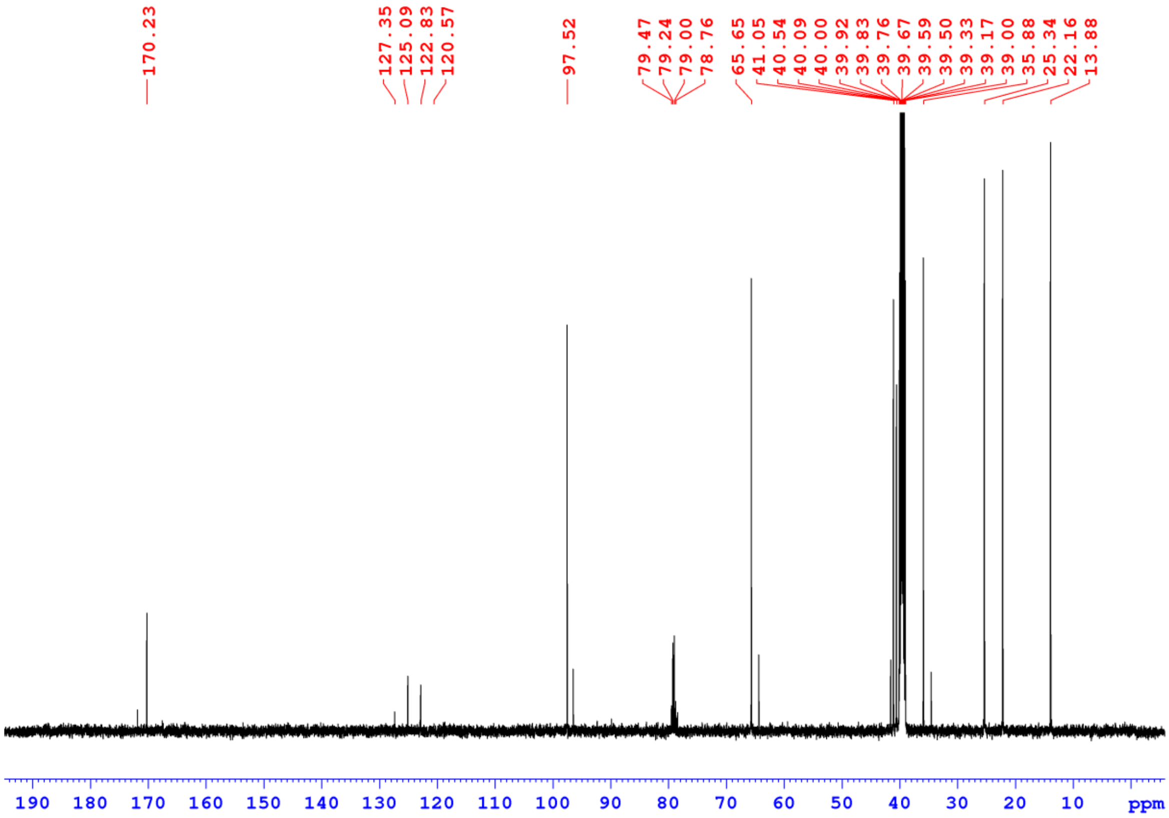

Figure S13. ^{13}C NMR spectrum of **4c**^{t} (mixed with **4c**^{c} in the ratio 90:10).

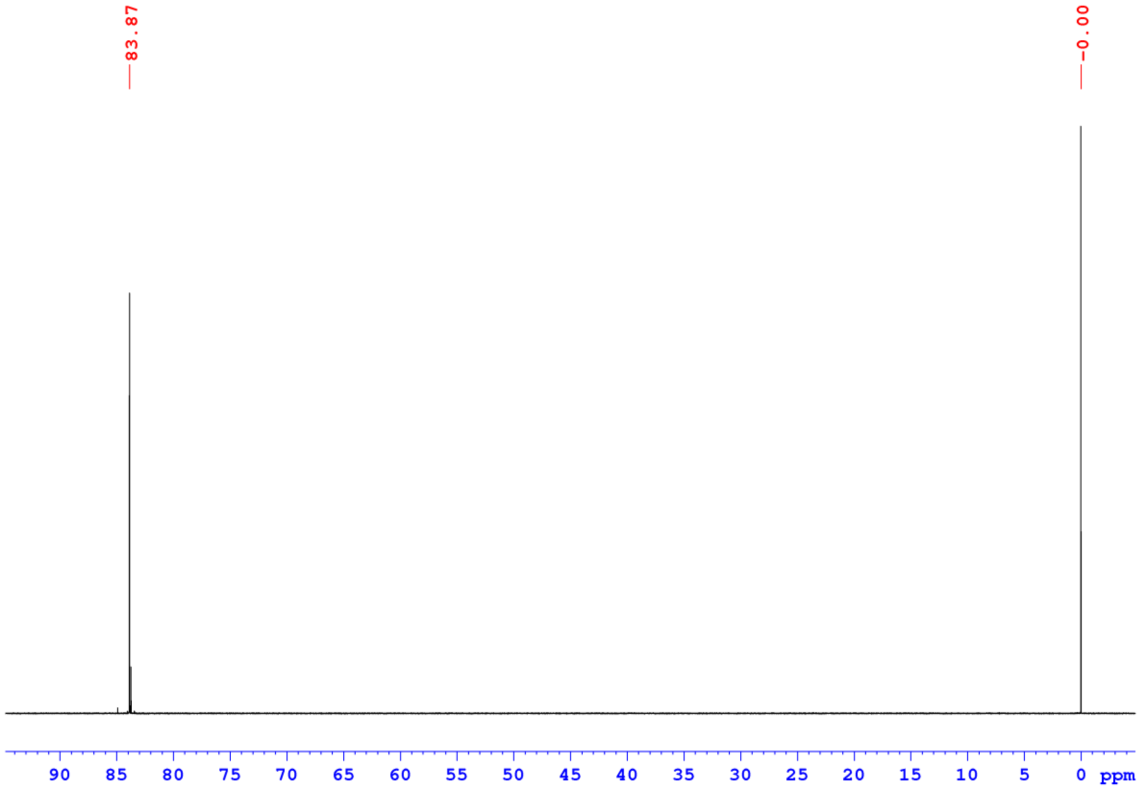

Figure S14. ^{19}F NMR spectrum of **4c**^{t} (mixed with **4c**^{c} in the ratio 90:10).

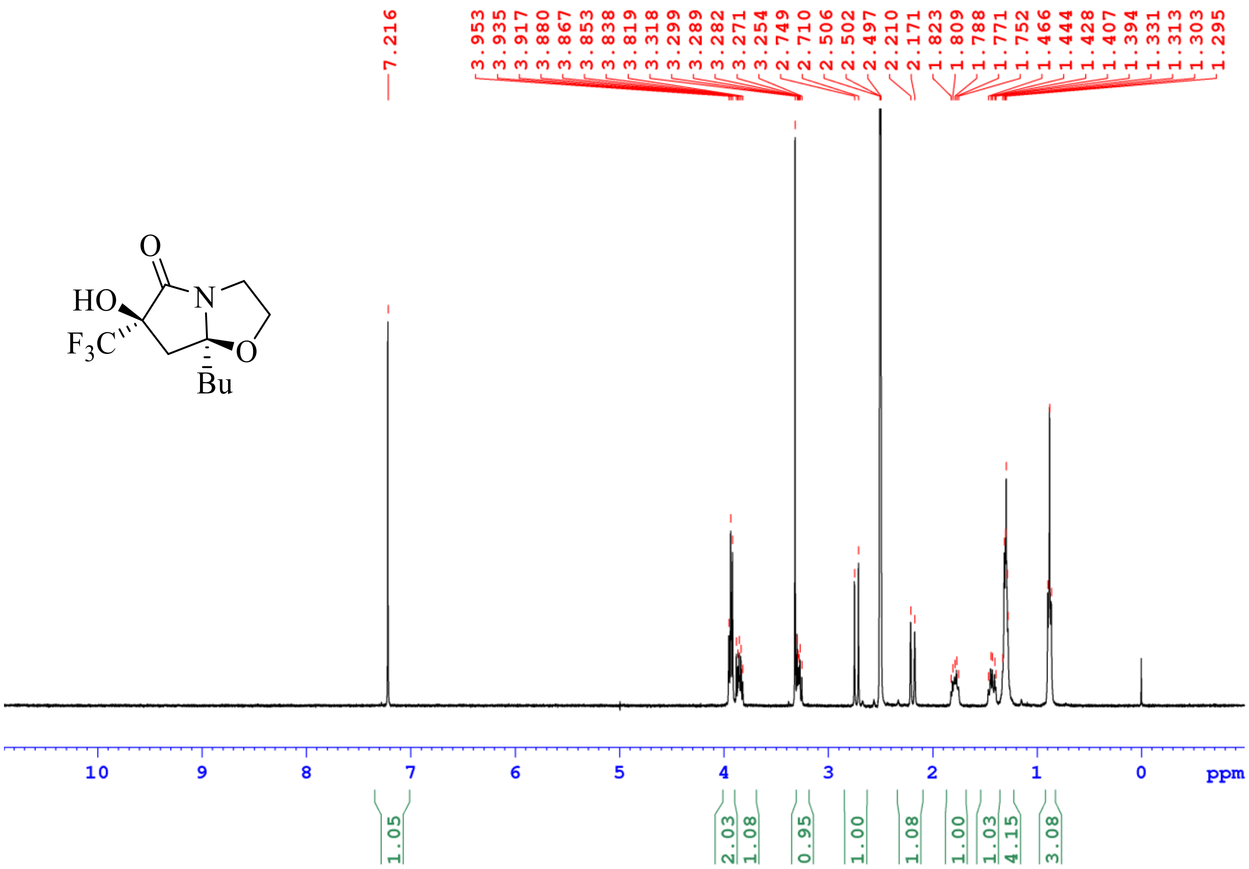

Figure S15. ^{1}H NMR spectrum of **4c**[c].

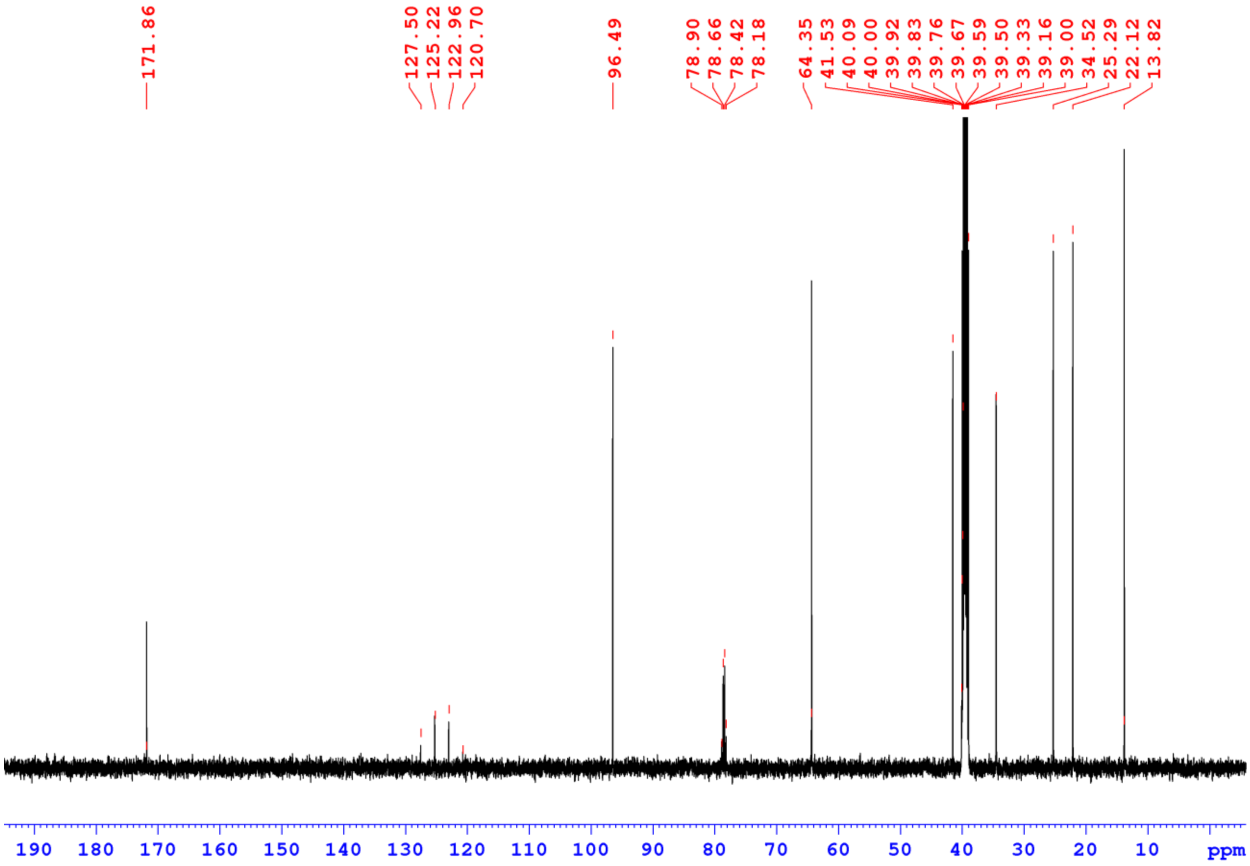

Figure S16. ^{13}C NMR spectrum of **4c**[c].

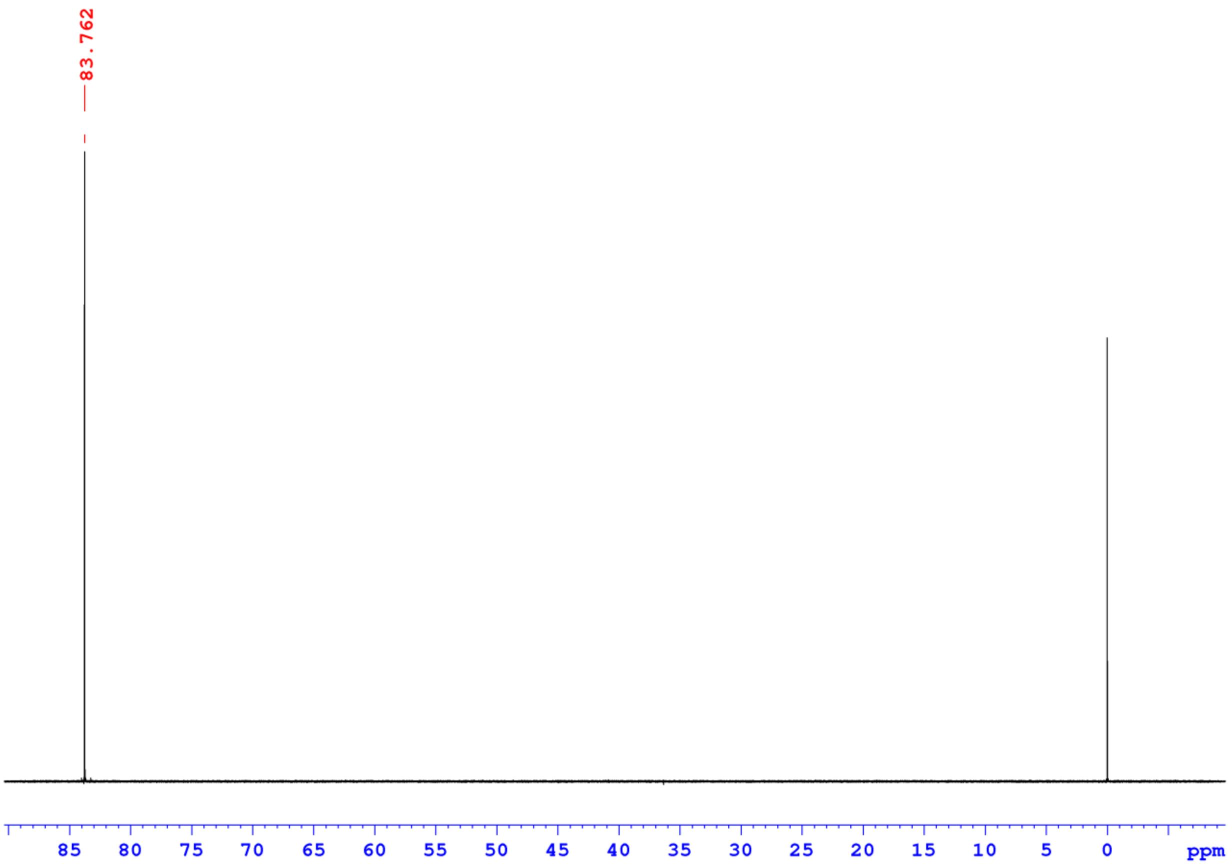

Figure S17. ^{19}F NMR spectrum of **4c**^{c}.

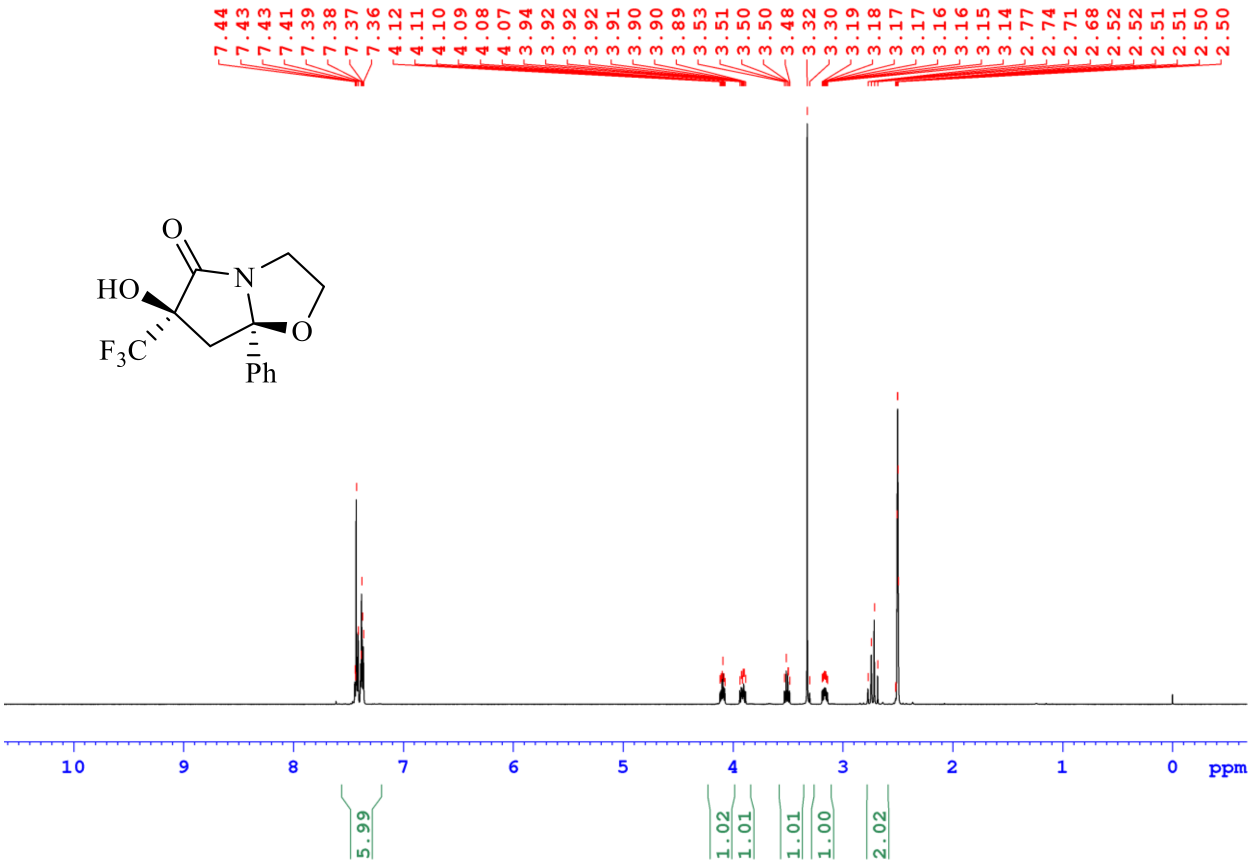

Figure S18. ^{1}H NMR spectrum of **4d**^{c}.

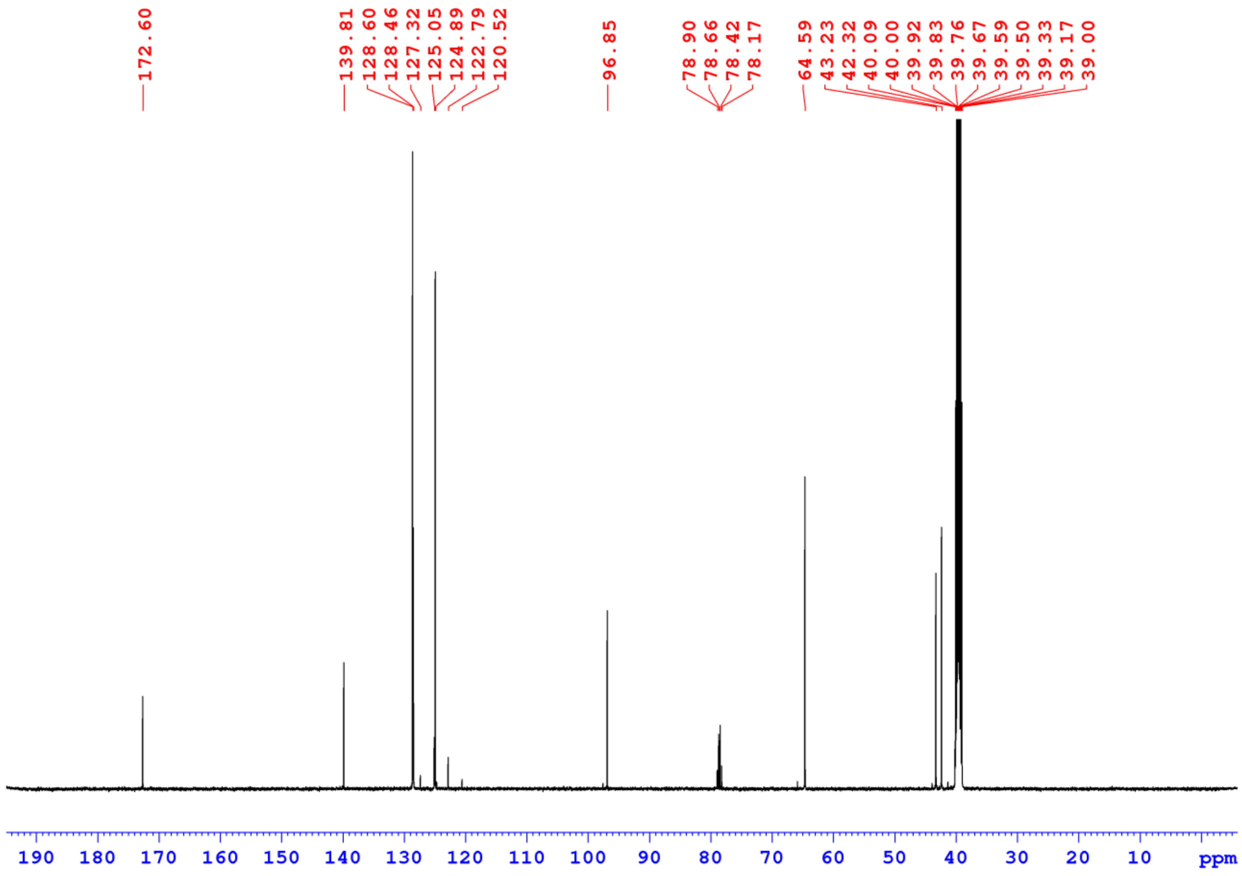

Figure S19. ^{13}C NMR spectrum of **4d^{c}.**

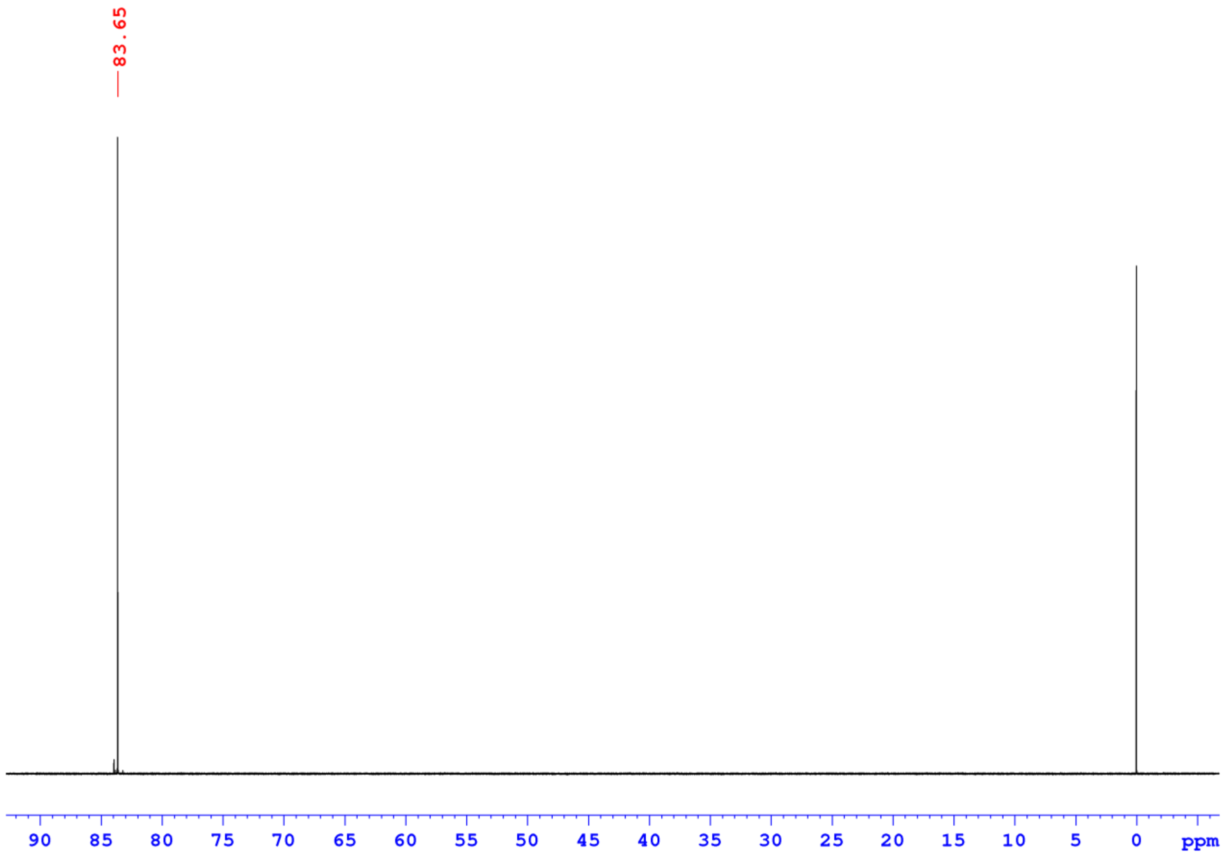

Figure S20. ^{19}F NMR spectrum of **4d^{c}.**

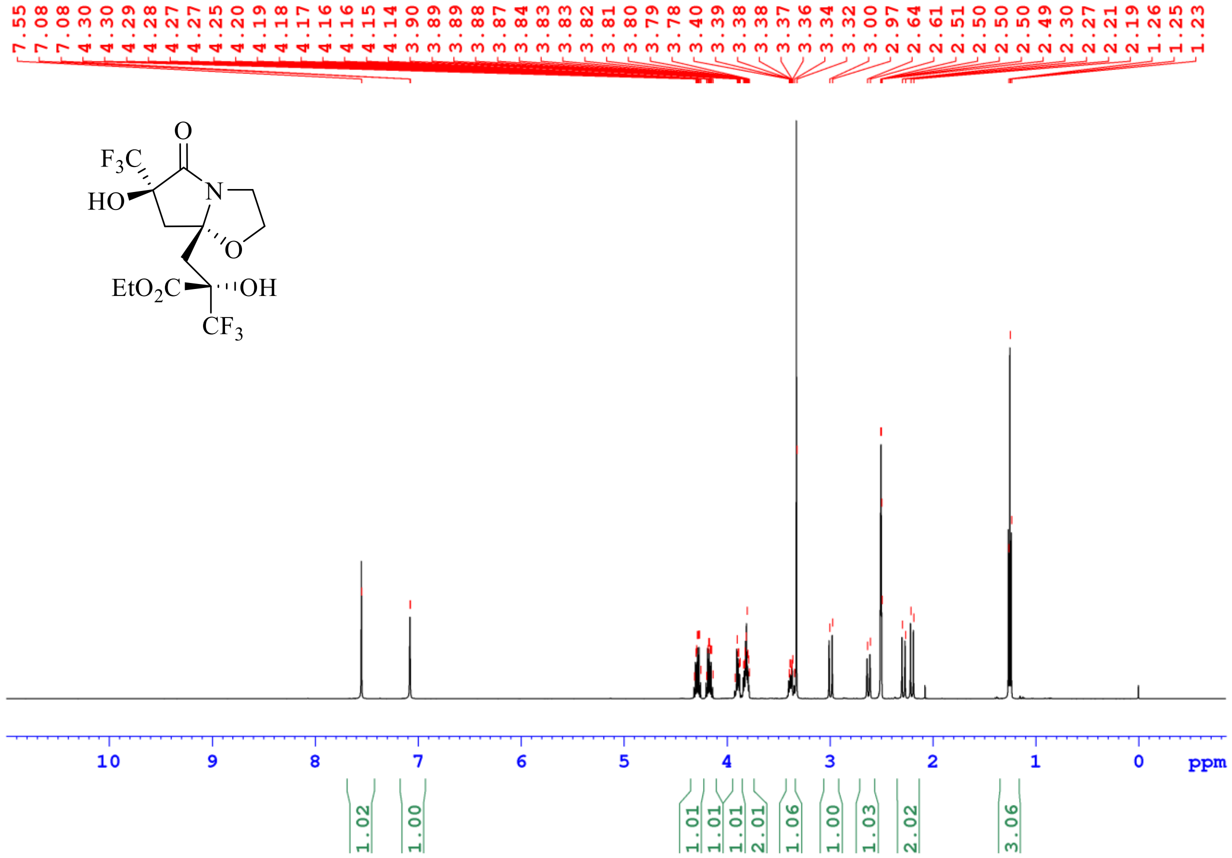

Figure S21. ^{1}H NMR spectrum of **5^{tc}.**

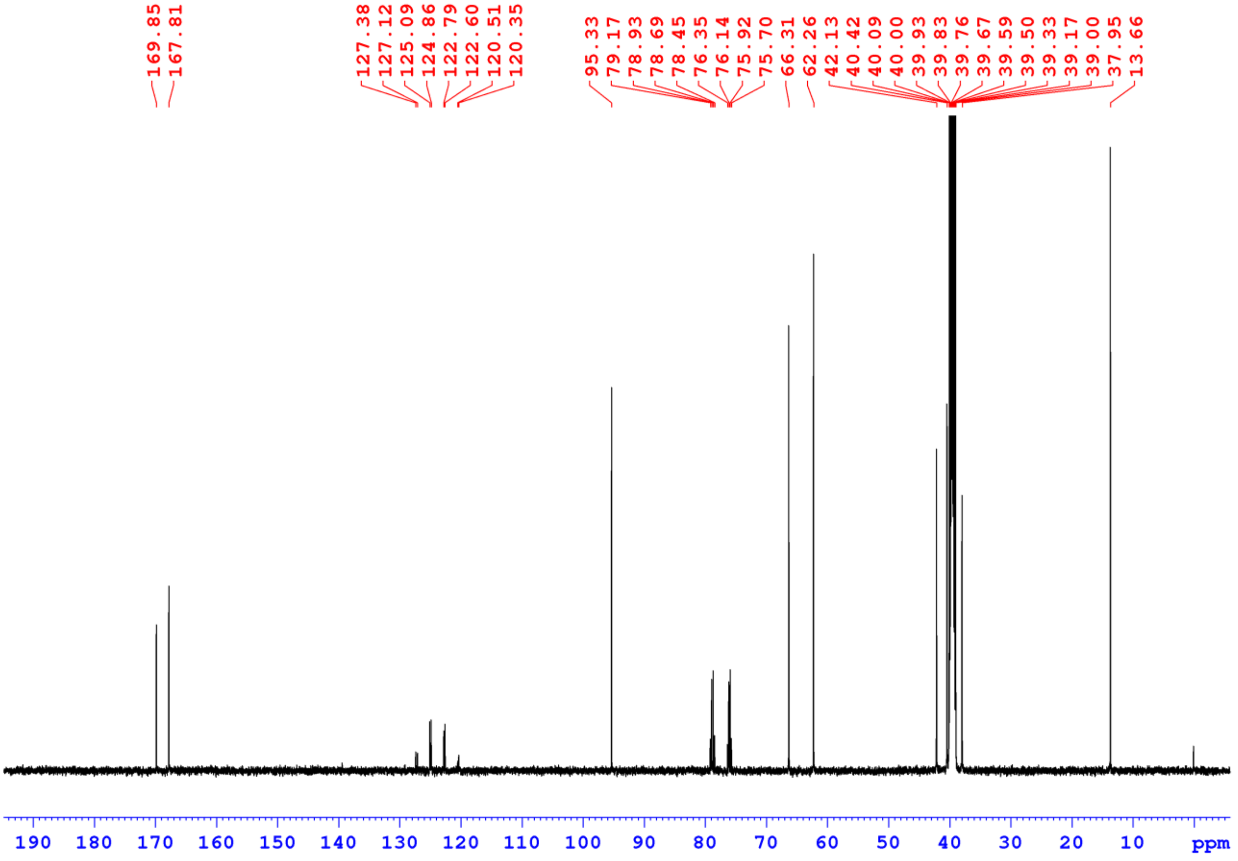

Figure S22. ^{13}C NMR spectrum of **5^{tc}**.

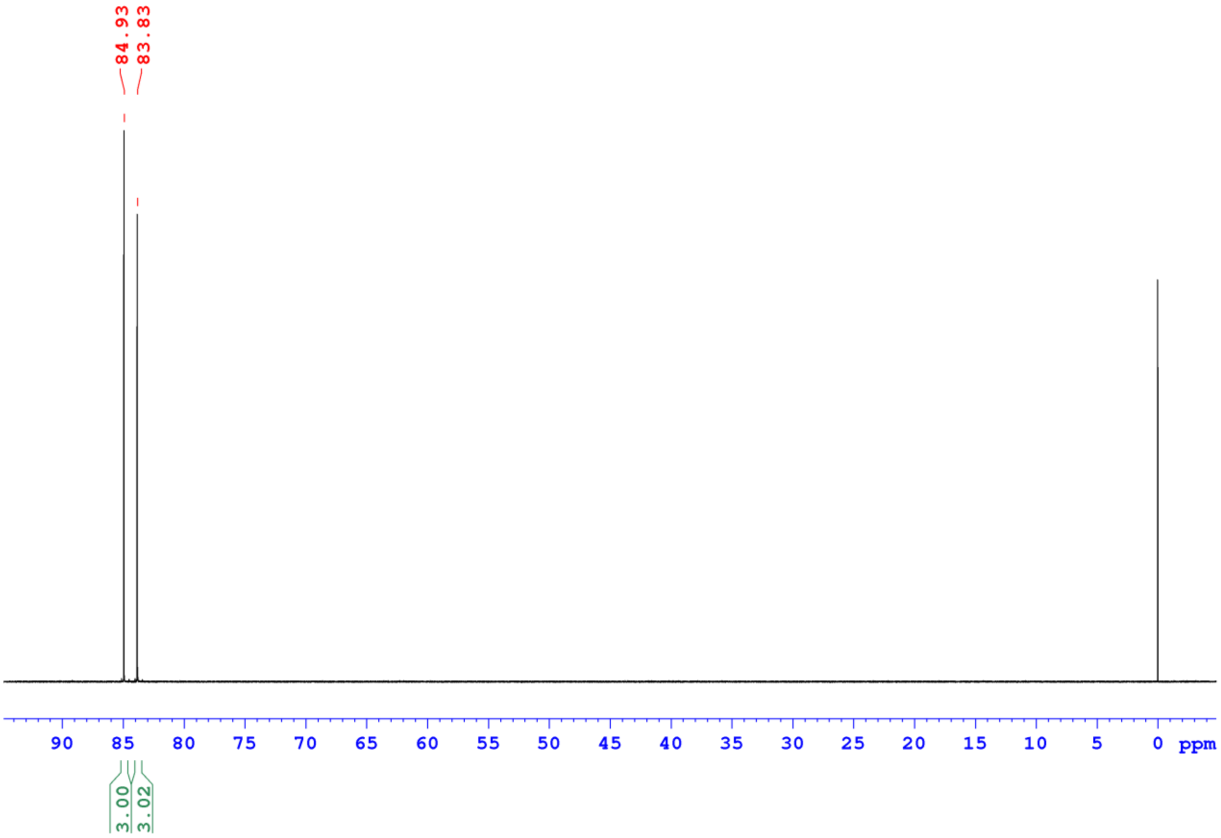

Figure S23. ^{19}F NMR spectrum of $\mathbf{5^{tc}}$.

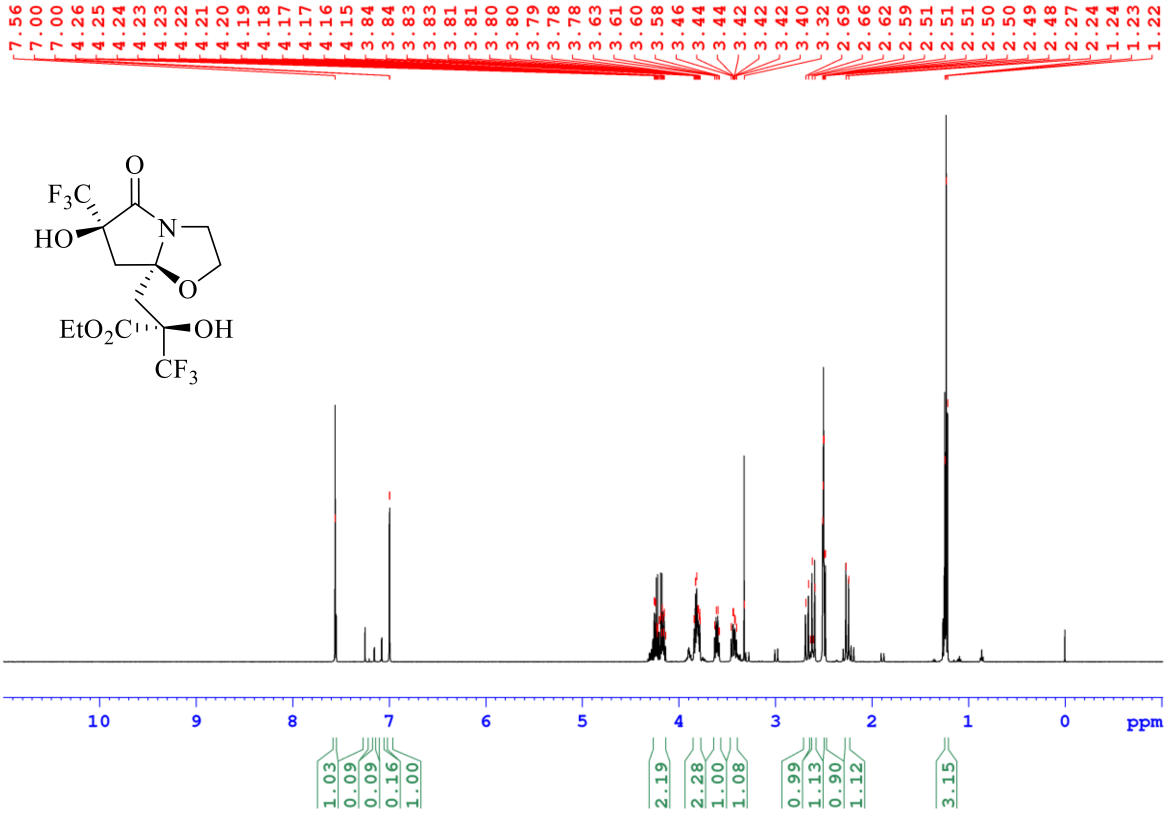

Figure S24. ^{1}H NMR spectrum of $\mathbf{5^{cc}}$ (mixed with $\mathbf{5^{tc}}$ in the ratio 80:20)

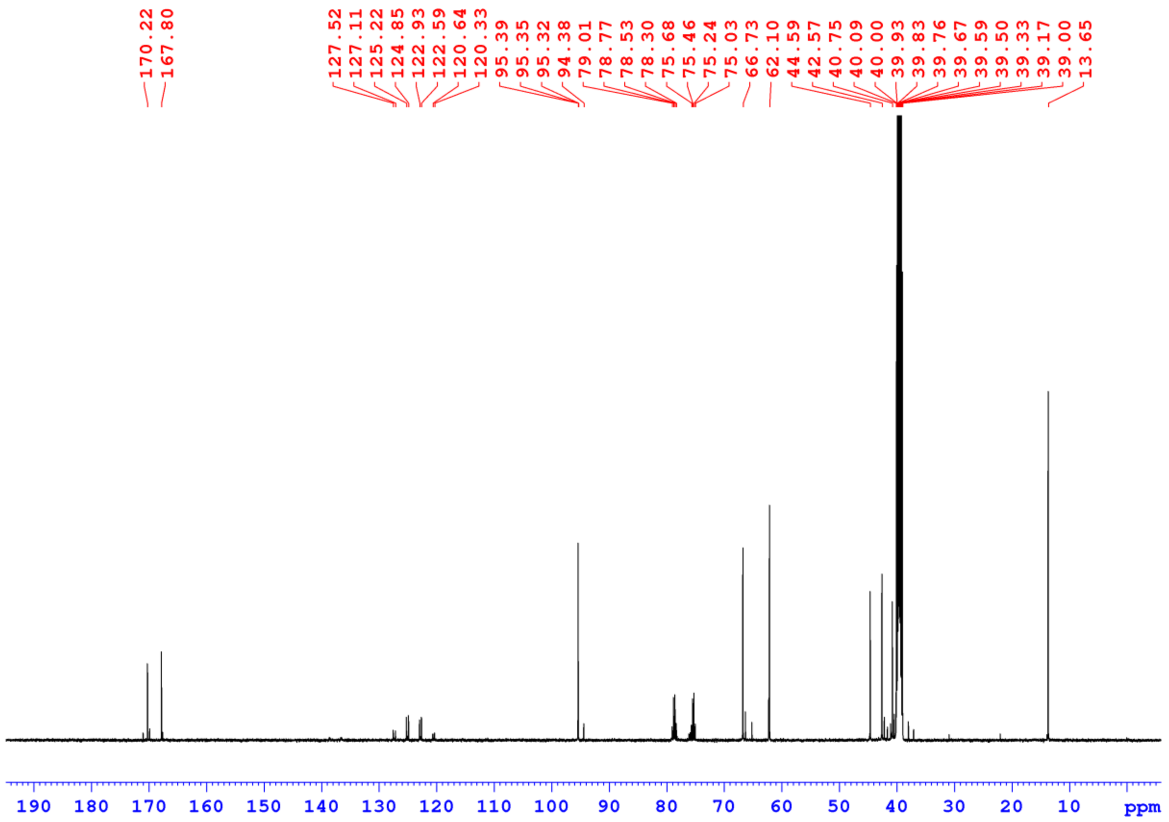

Figure S25. ^{13}C NMR spectrum of $\mathbf{5^{cc}}$ (mixed with $\mathbf{5^{tc}}$ in the ratio 80:20).

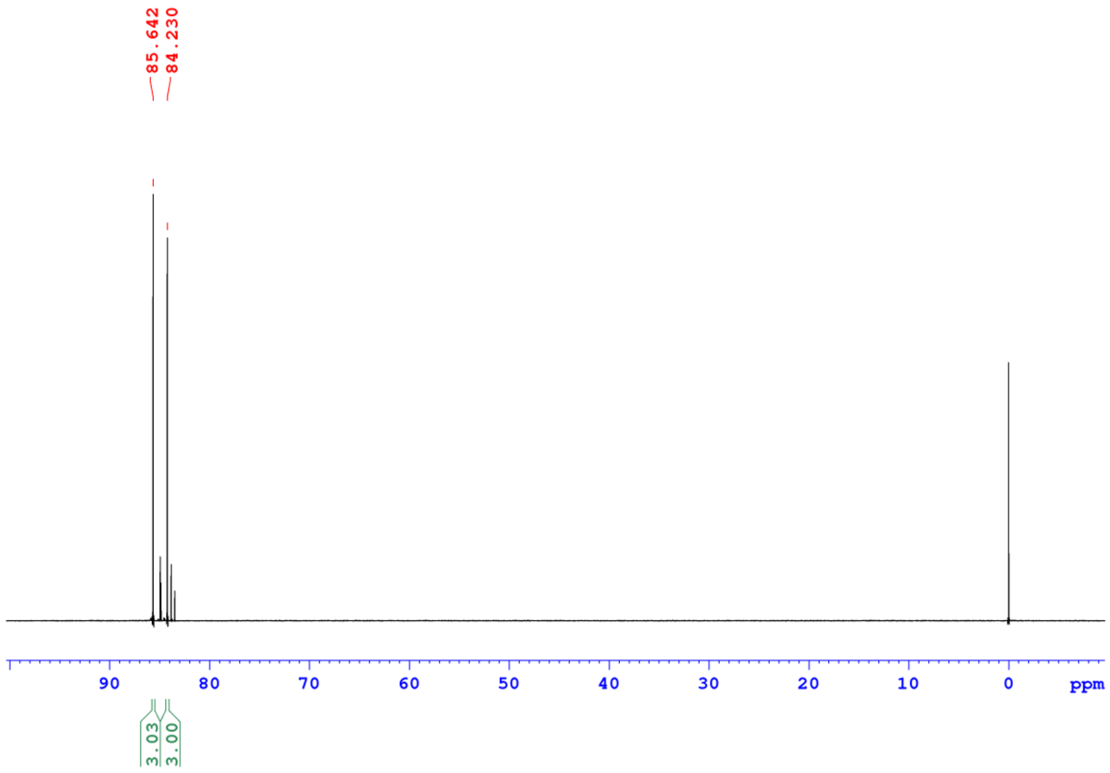

Figure S26. ^{19}F NMR spectrum of $\mathbf{5^{cc}}$ (mixed with $\mathbf{5^{tc}}$ in the ratio 80:20).

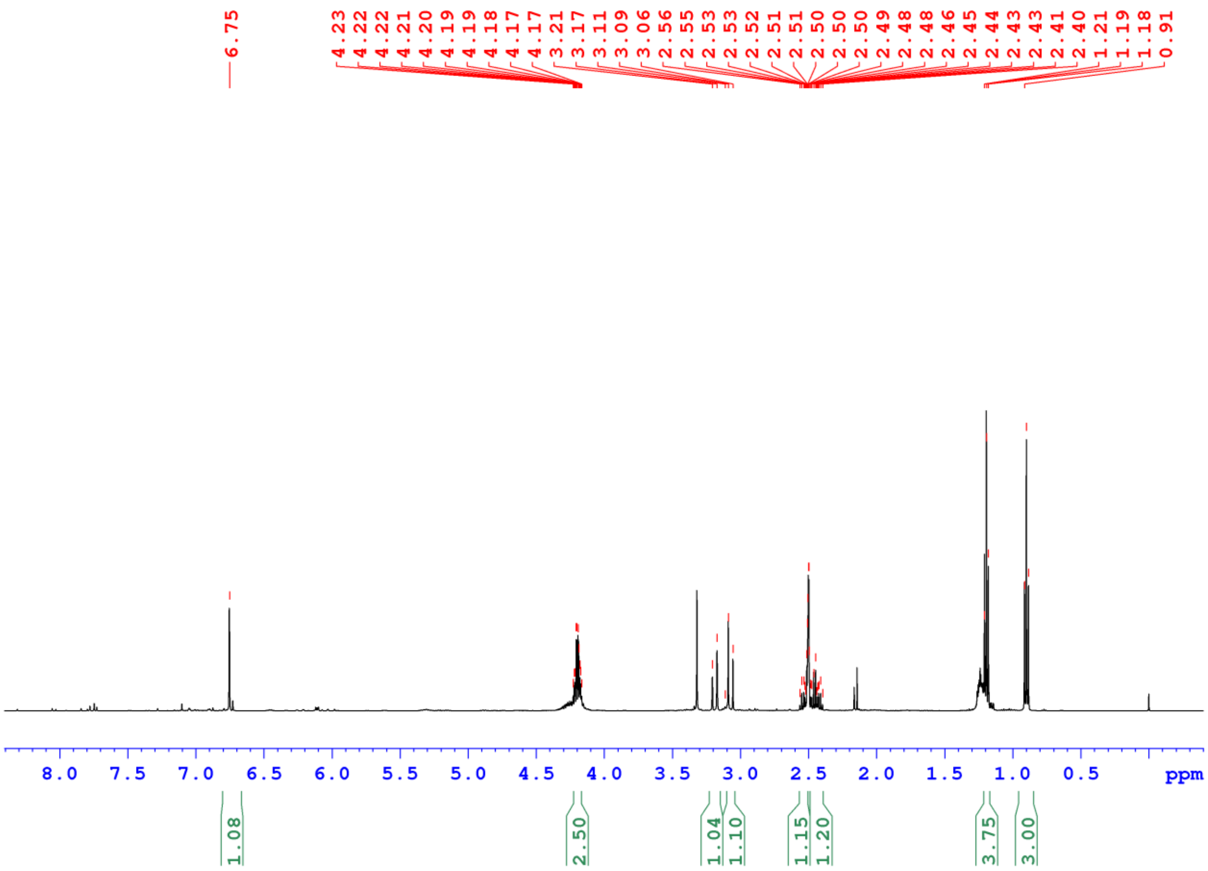

Figure S27. ^{1}H NMR spectrum of **6b.**

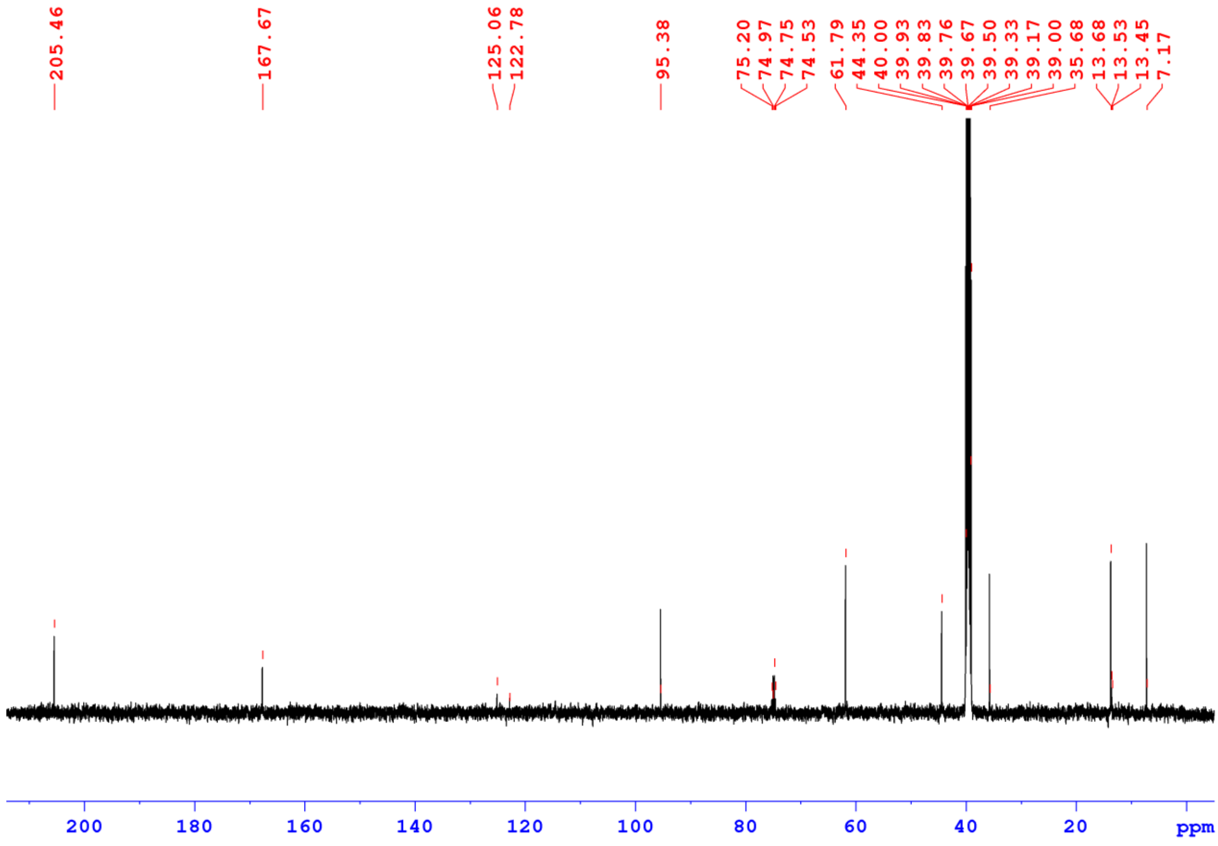

Figure S28. ^{13}C NMR spectrum of **6b.**

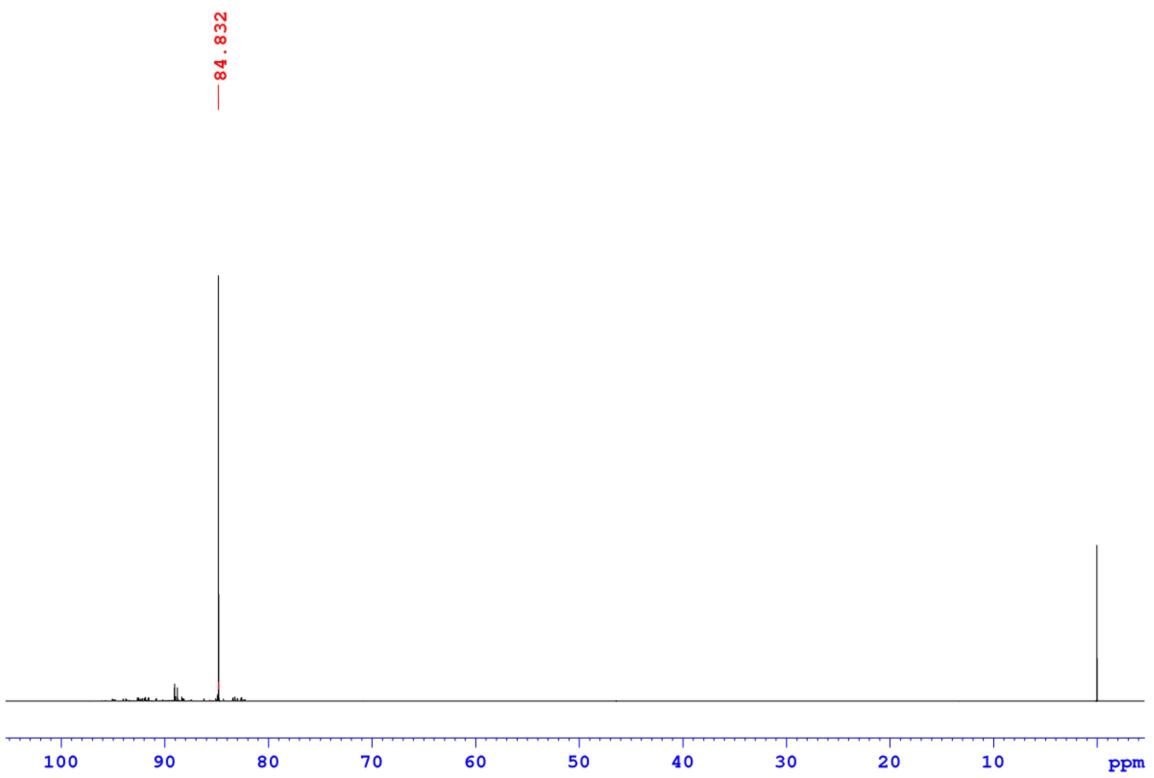

Figure S29. ^{19}F NMR spectrum of **6b.**

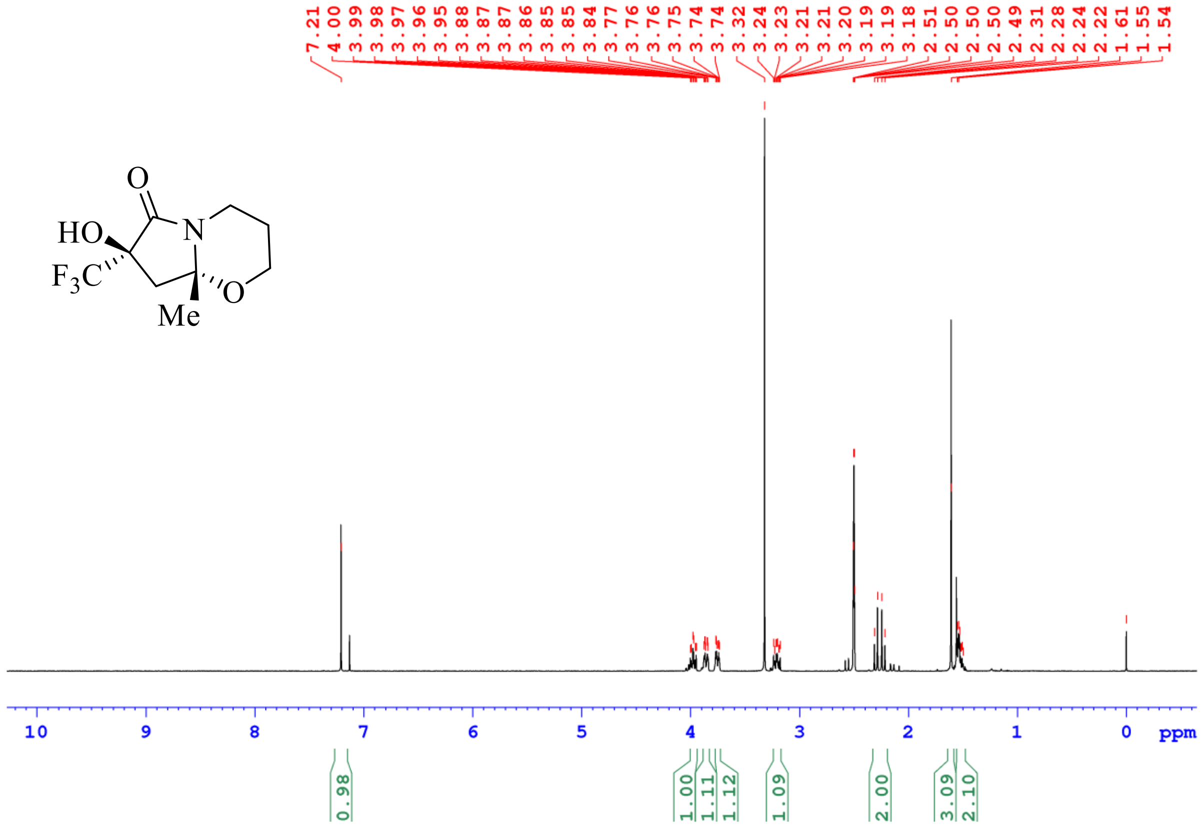

Figure S30. ^{1}H NMR spectrum of **8a^{t}** (mixed with **8a^{c}** in the ratio 84:16).

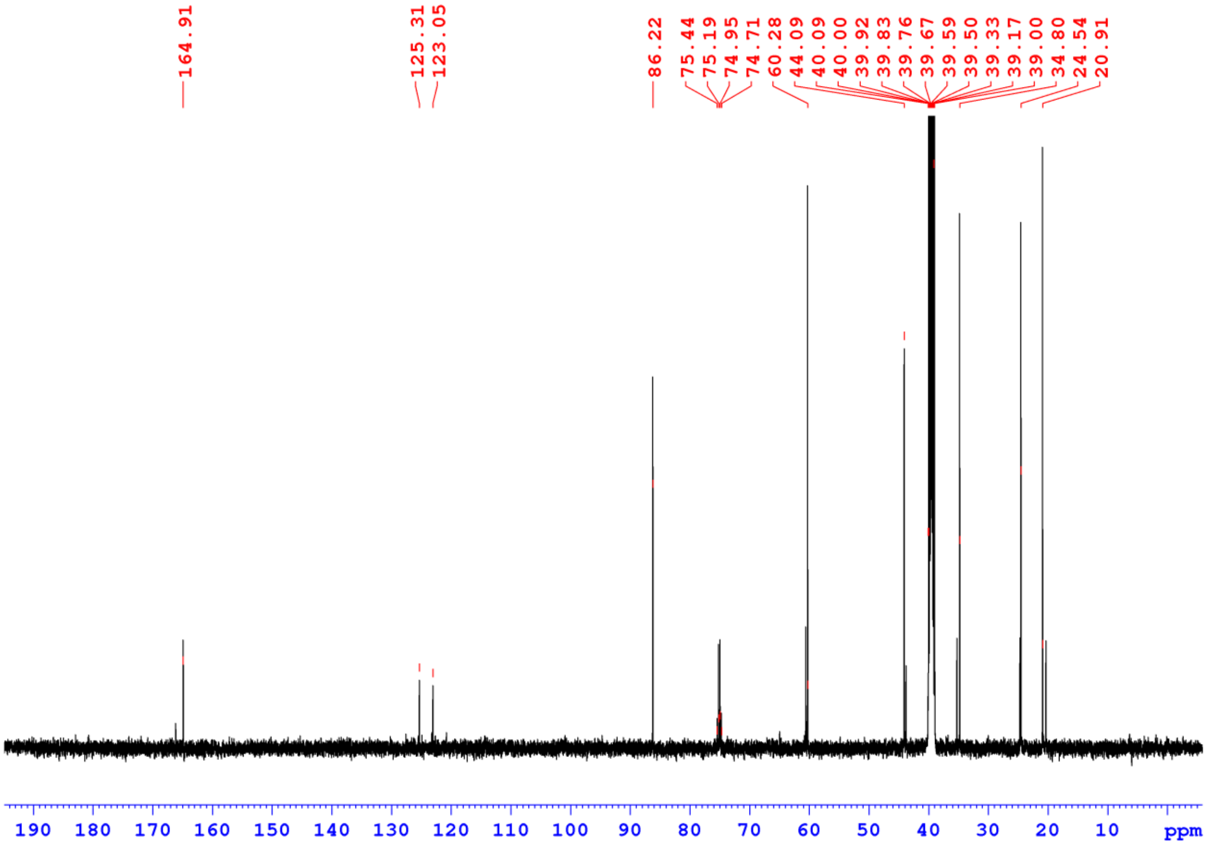

Figure S31. ^{13}C NMR spectrum of **8a^{t}** (mixed with **8a^{c}** in the ratio 84:16).

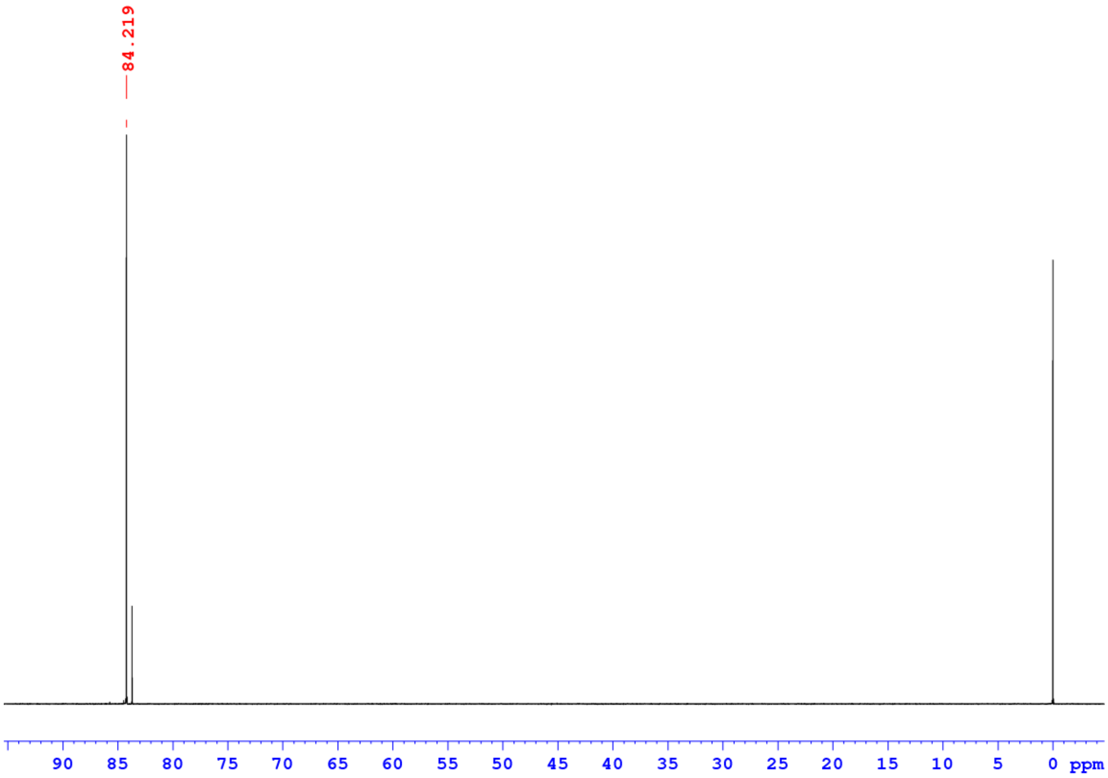

Figure S32. ^{19}F NMR spectrum of **8a^{t}** (mixed with **8a^{c}** in the ratio 84:16).

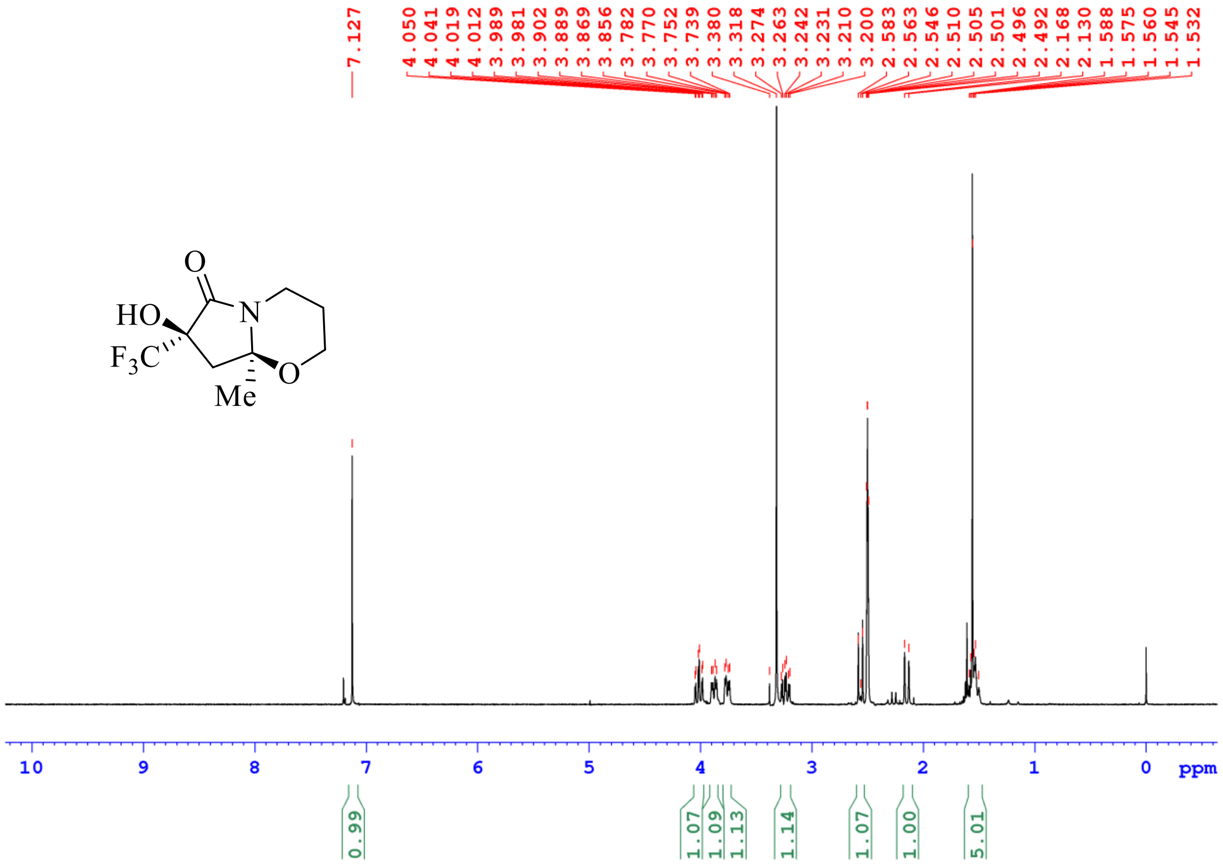

Figure S33. ^{1}H NMR spectrum of **8a**^{c} (mixed with **8a**^{t} in the ratio 89:11).

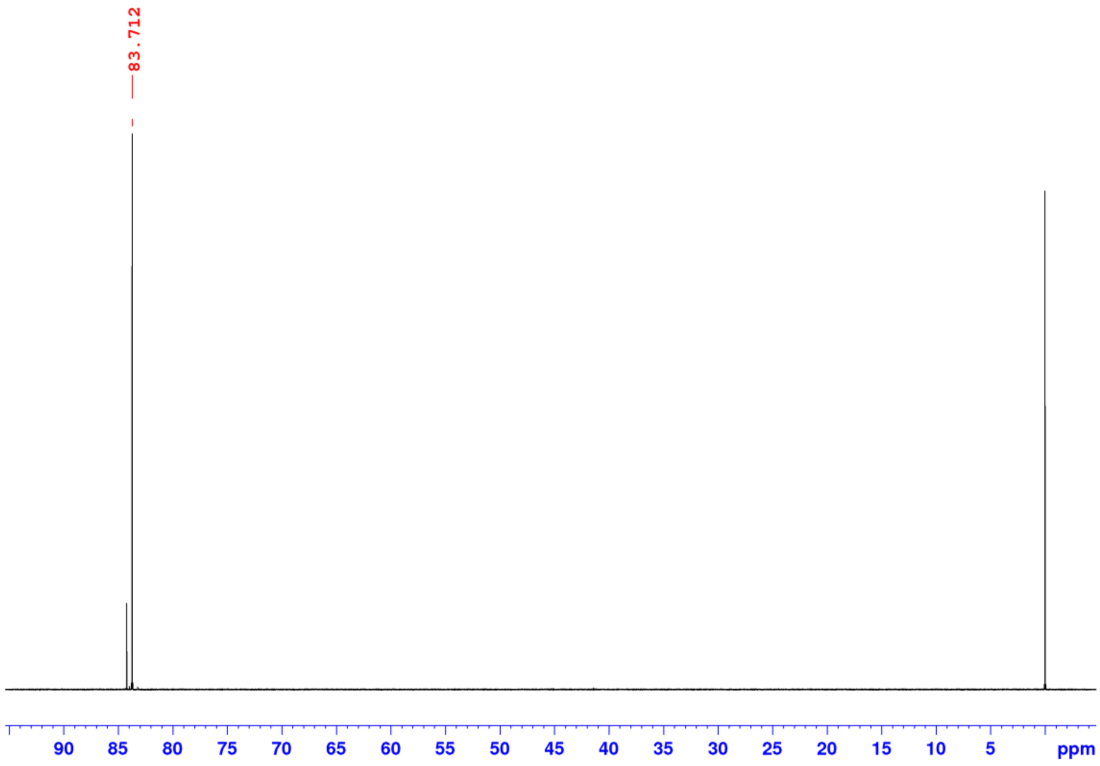

Figure S34. ^{19}F NMR spectrum of **8a**^{c} (mixed with **8a**^{t} in the ratio 89:11).

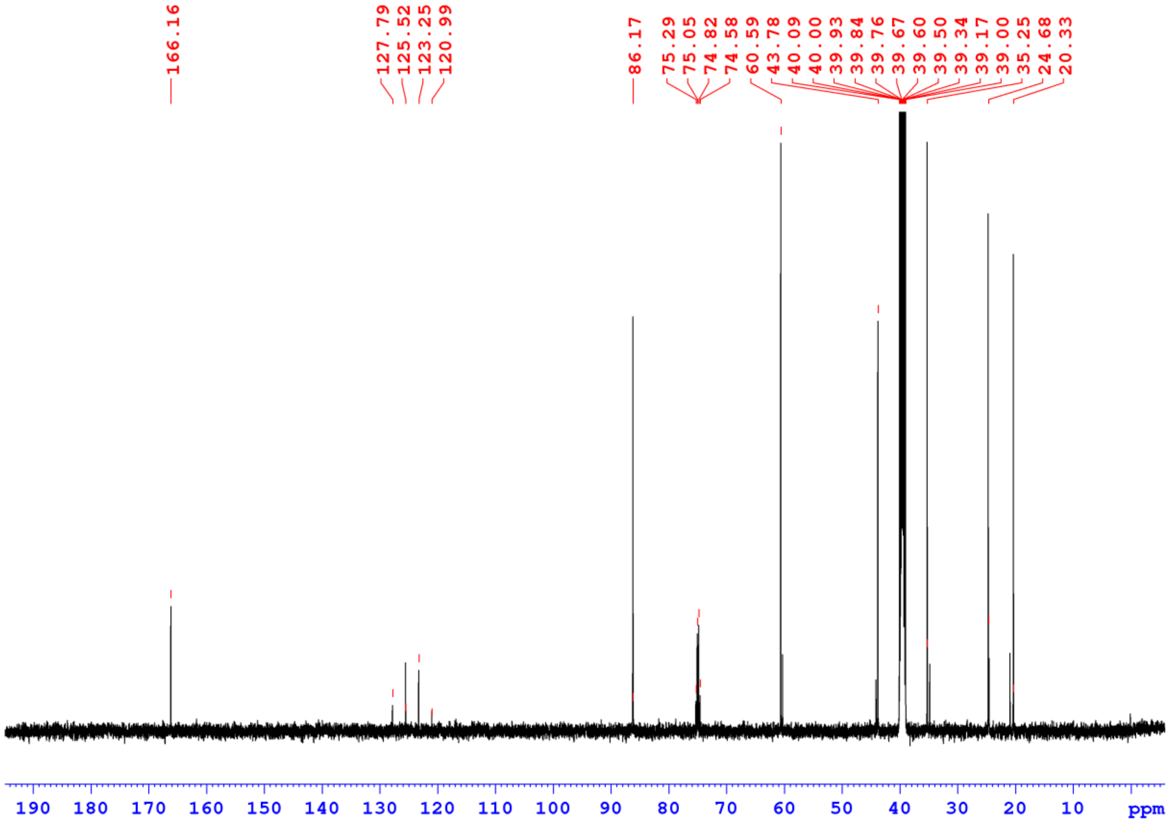

Figure S35. ^{13}C NMR spectrum of **8a^{c}** (mixed with **8a^{t}** in the ratio 89:11).

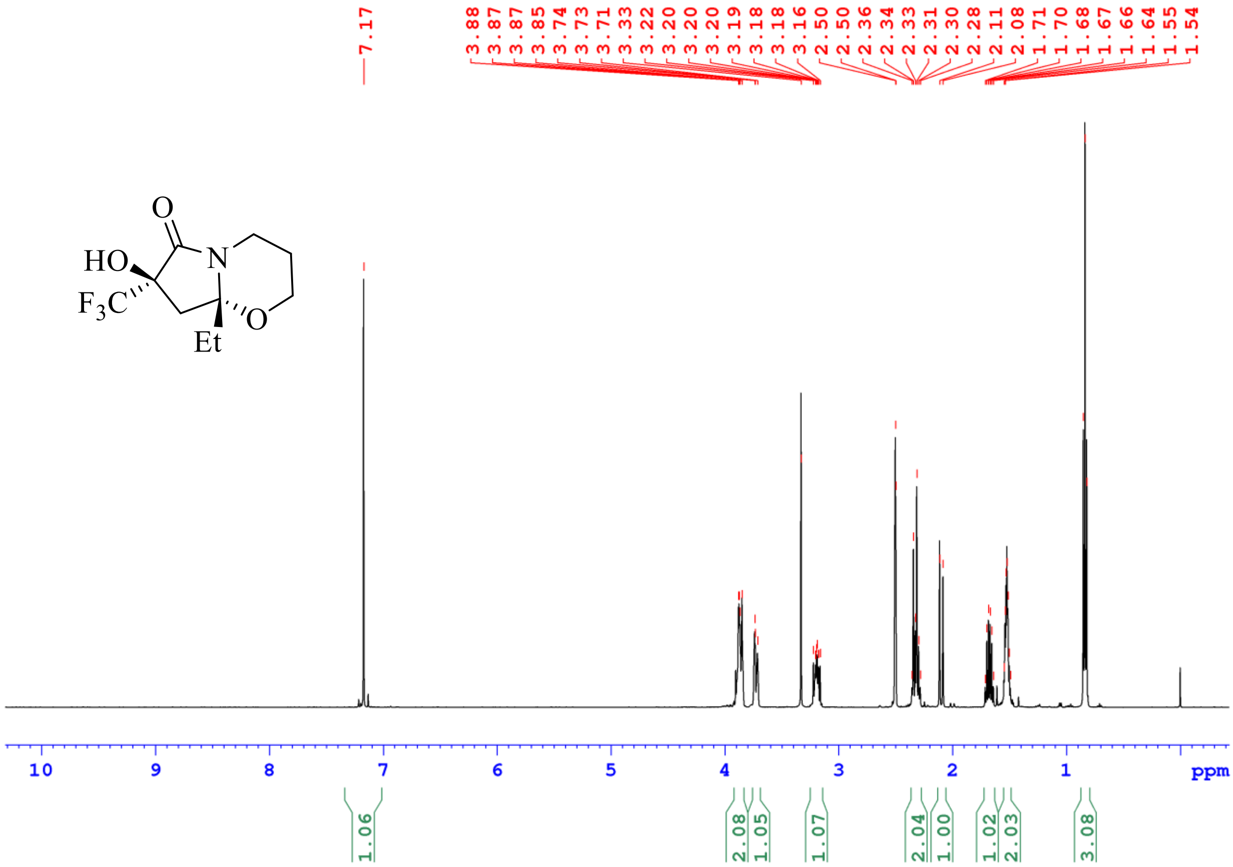

Figure S36. ^{1}H NMR spectrum of **8b^{t}**.

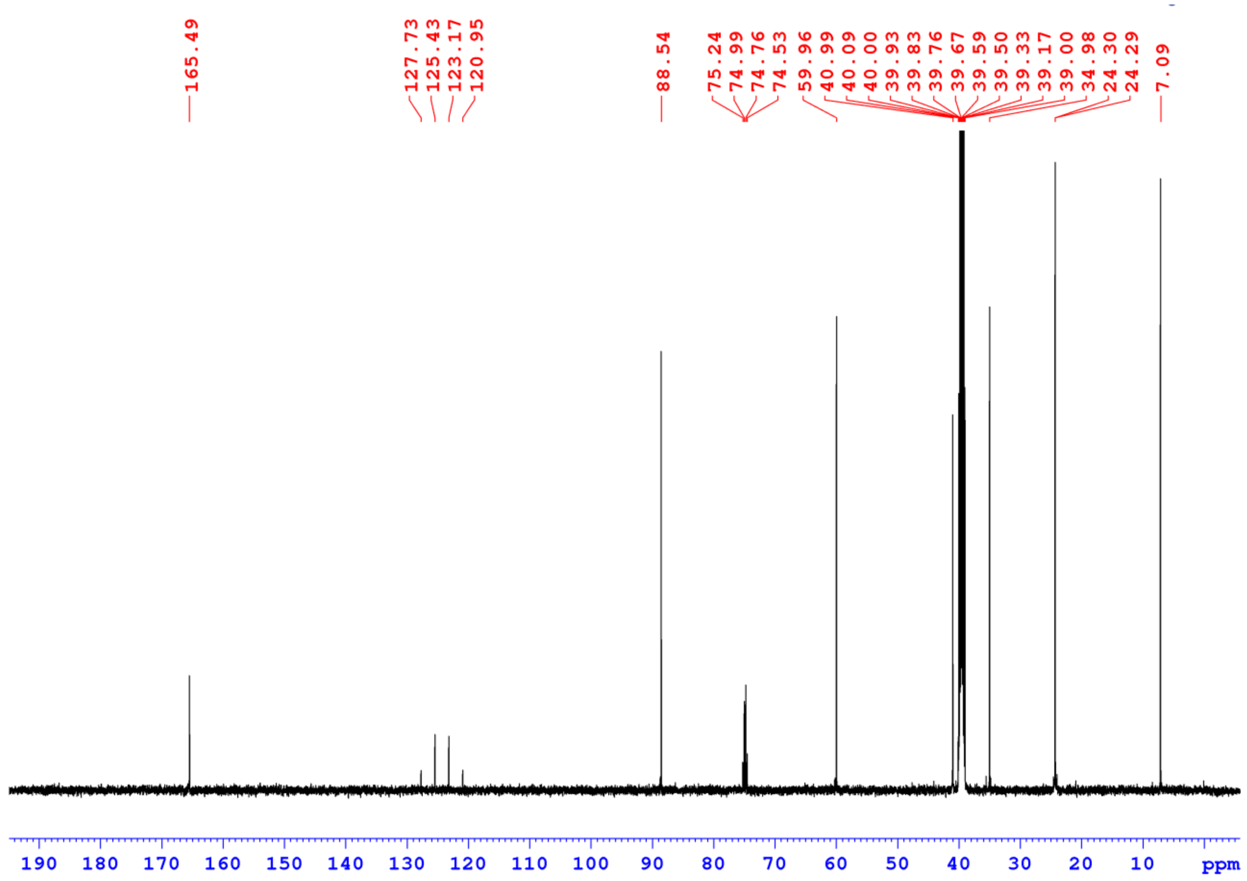

Figure S37. ^{13}C NMR spectrum of **8b**^{t}.

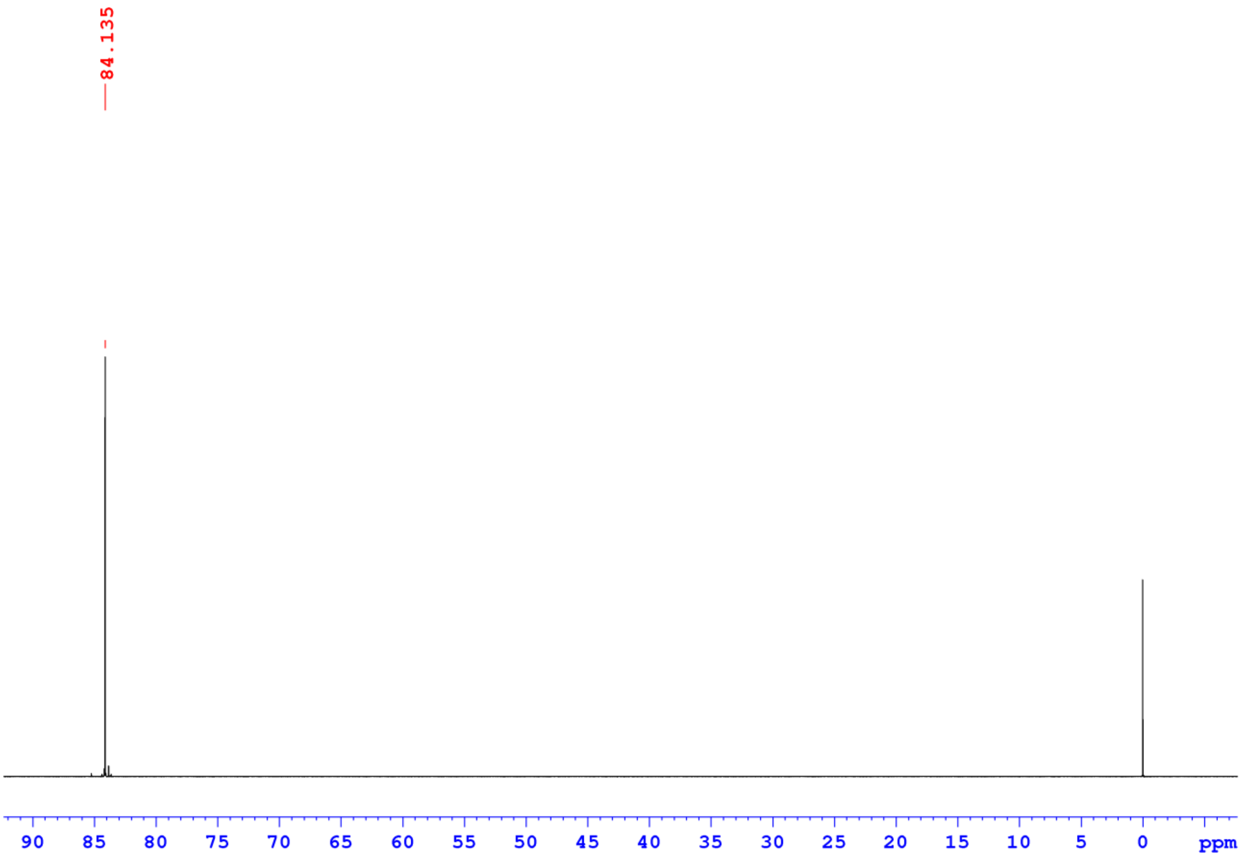

Figure S38. ^{19}F NMR spectrum of **8b**^{t}.

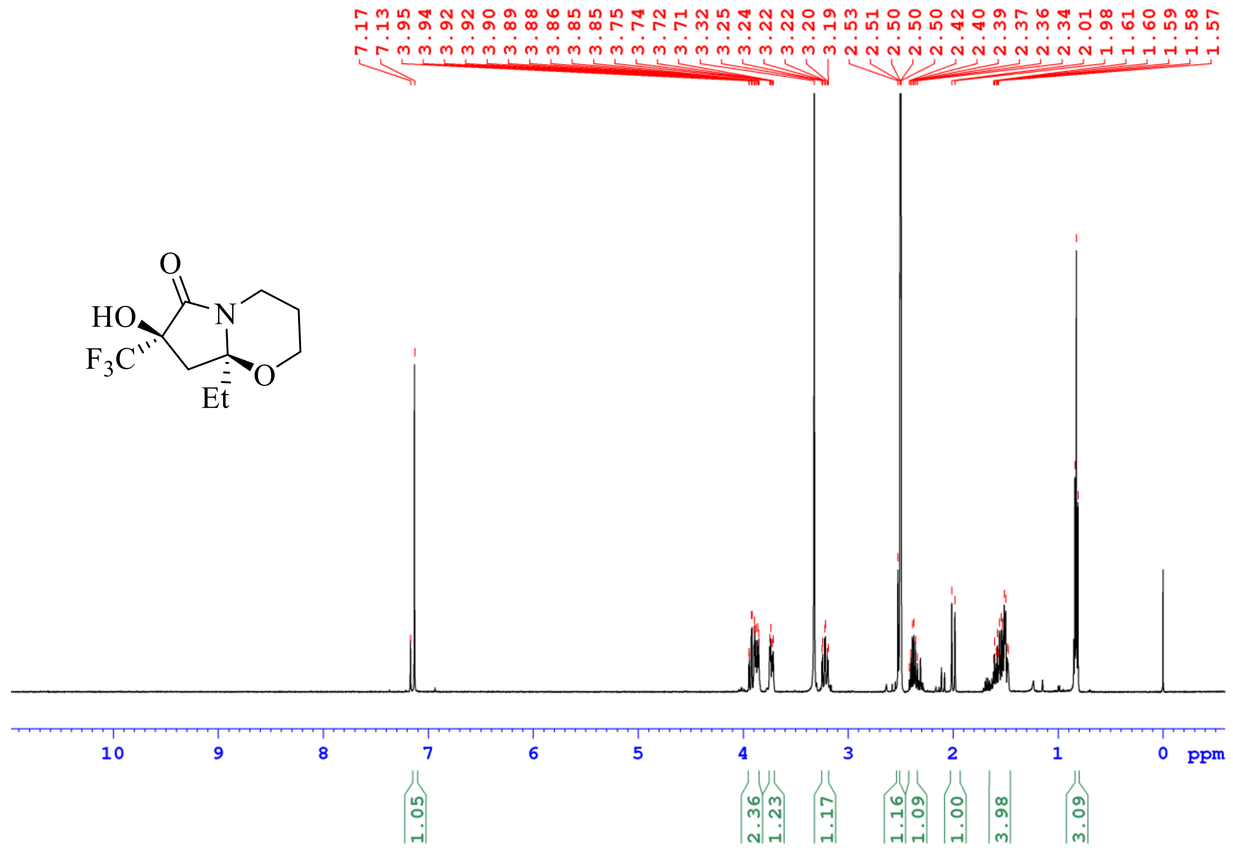

Figure S39. ^{1}H NMR spectrum of **8b^{c}** (mixed with **8b^{t}** in the ratio 81:19).

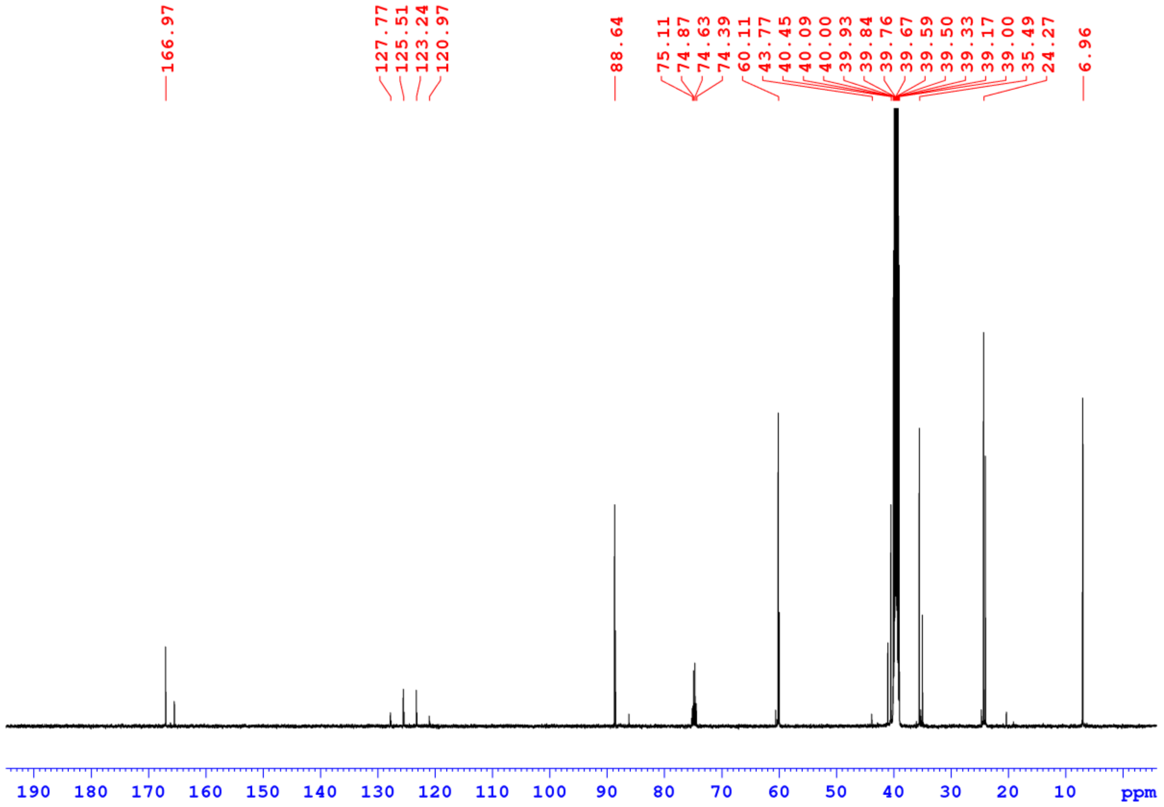

Figure S40. ^{13}C NMR spectrum of **8b^{c}** (mixed with **8b^{t}** in the ratio 81:19).

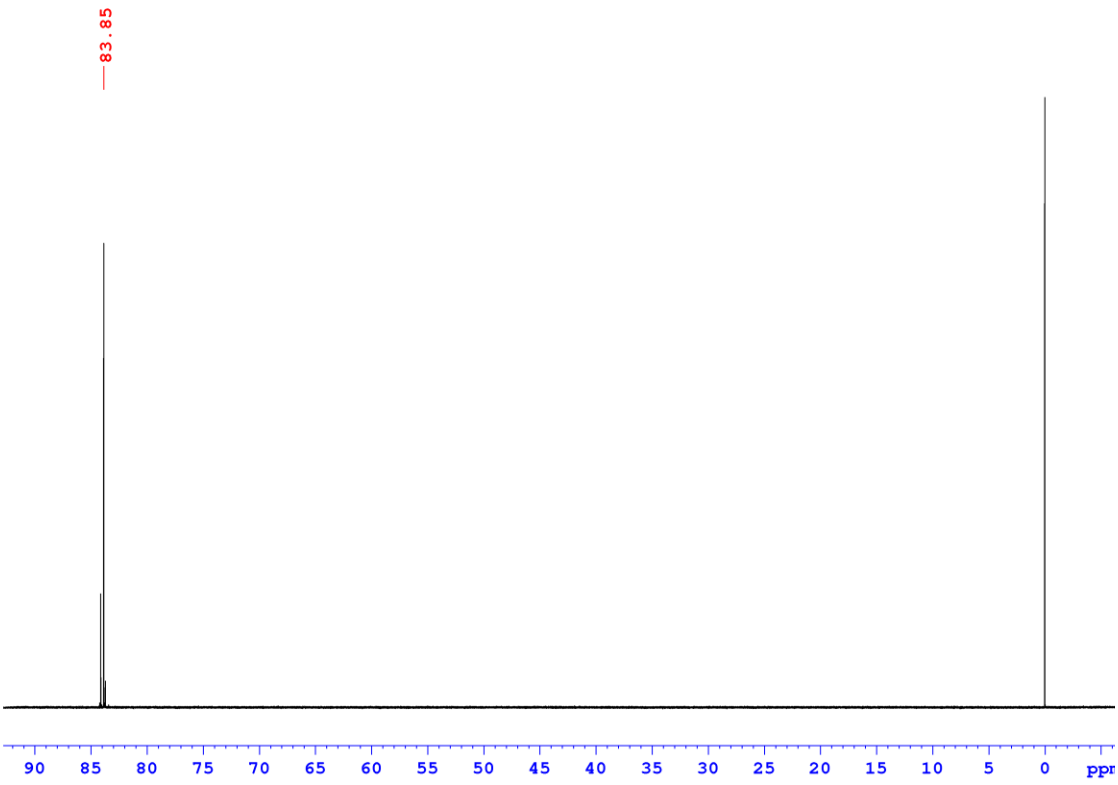

Figure S41. ^{19}F NMR spectrum of **8b**^{c} (mixed with **8b**^{t} in the ratio 81:19).

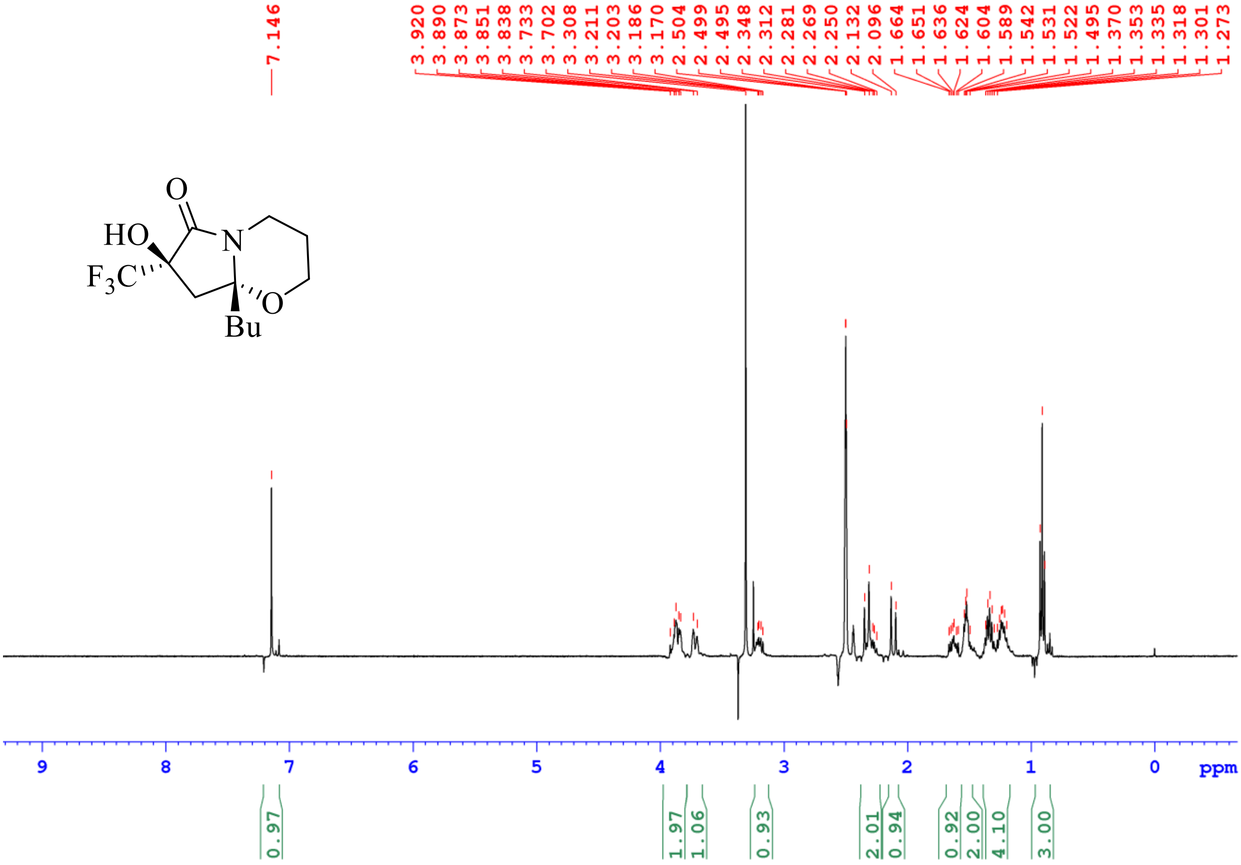

Figure S42. ^{1}H NMR spectrum of **8c**^{t}.

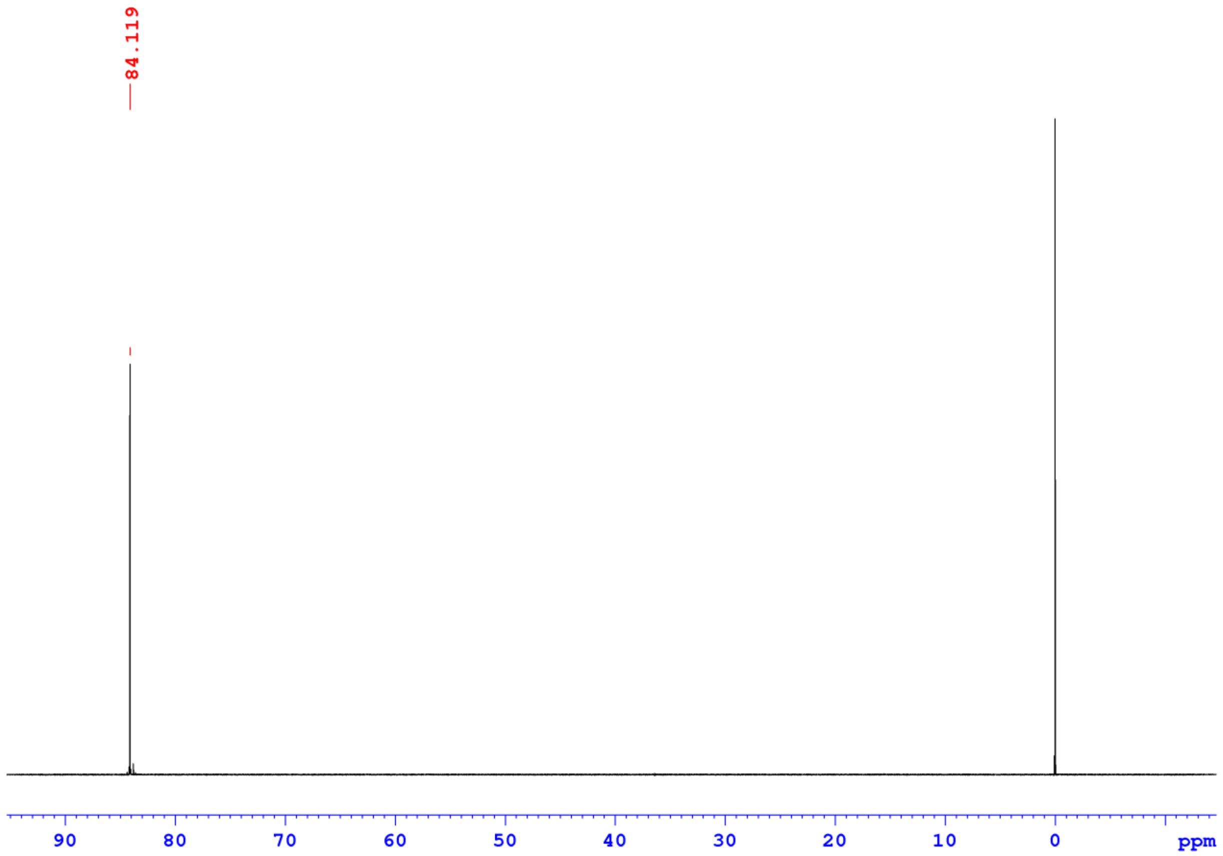

Figure S43. ^{19}F NMR spectrum of **8c^{t}**.

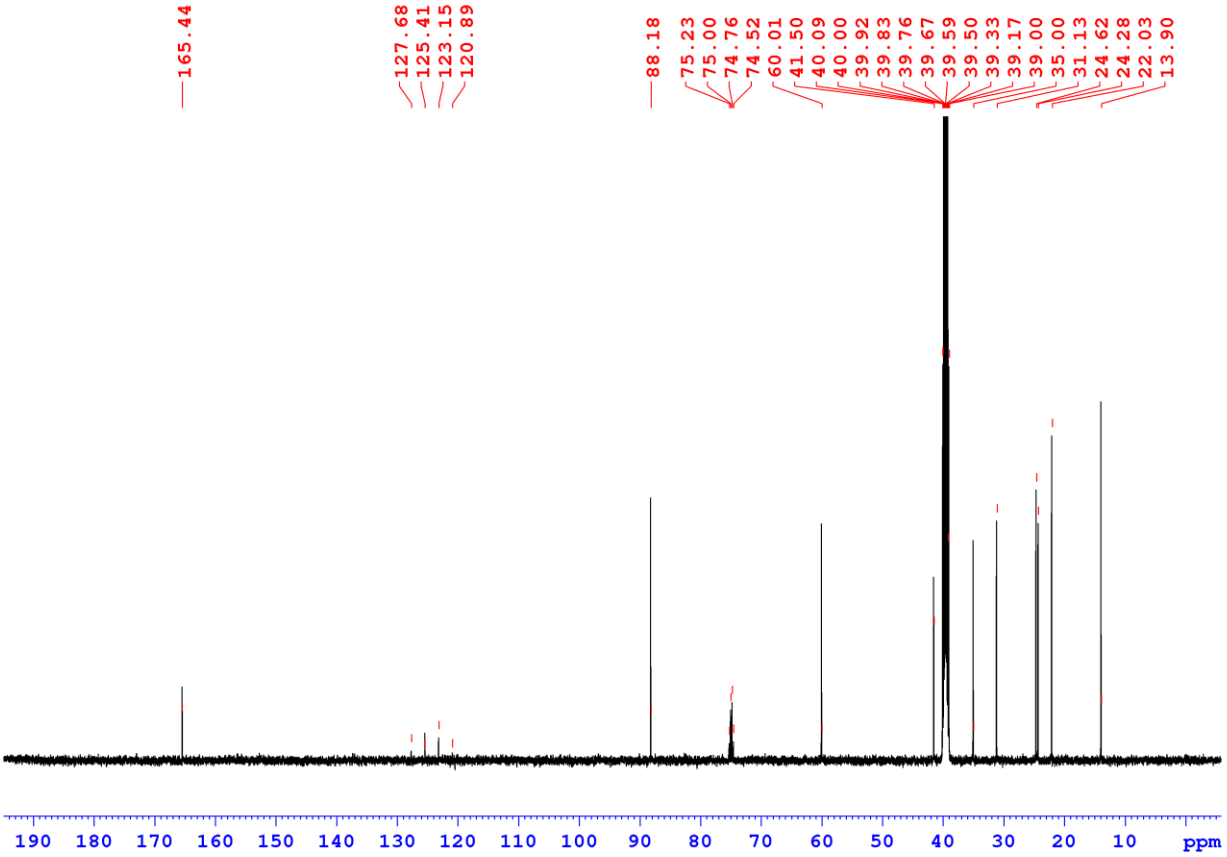

Figure S44. ^{13}C NMR spectrum of **8c^{t}**.

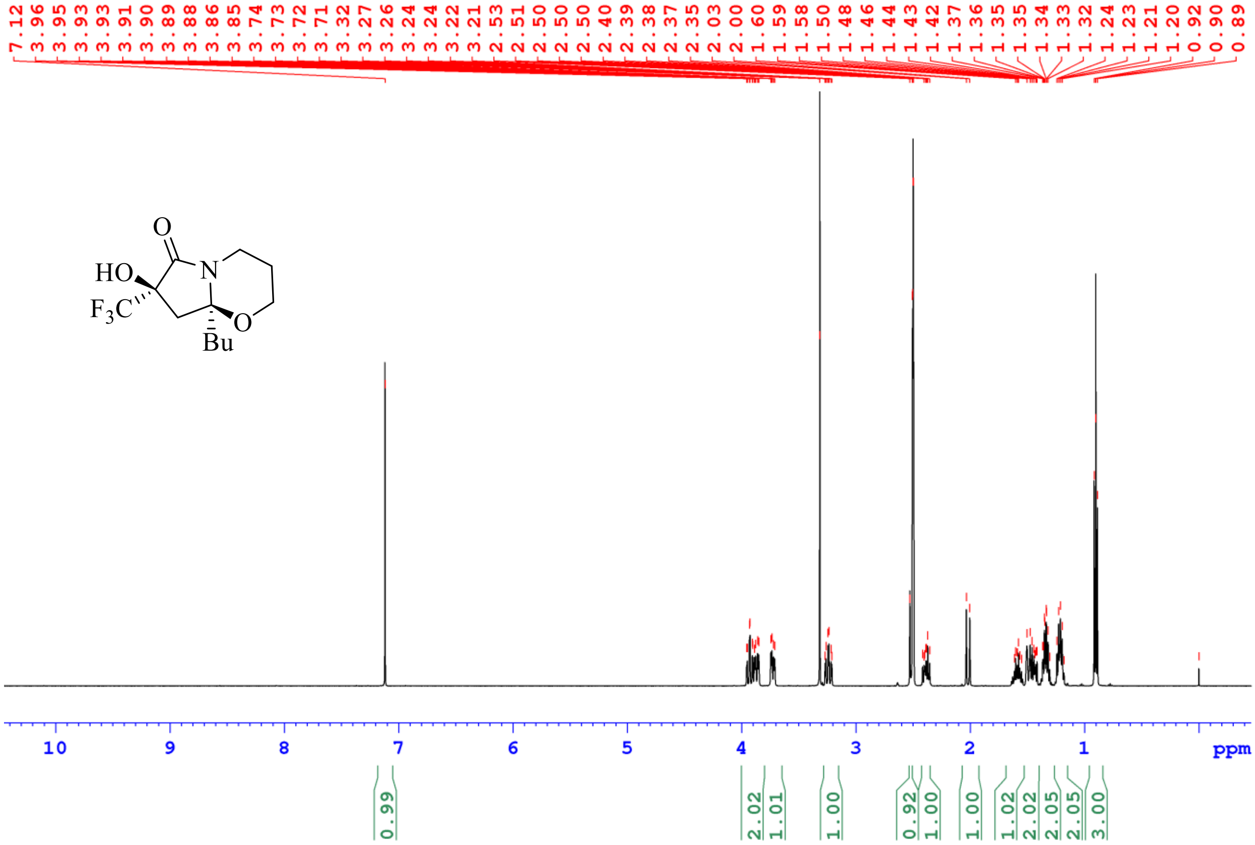

Figure S45. ^{1}H NMR spectrum of **8c**^{c}.

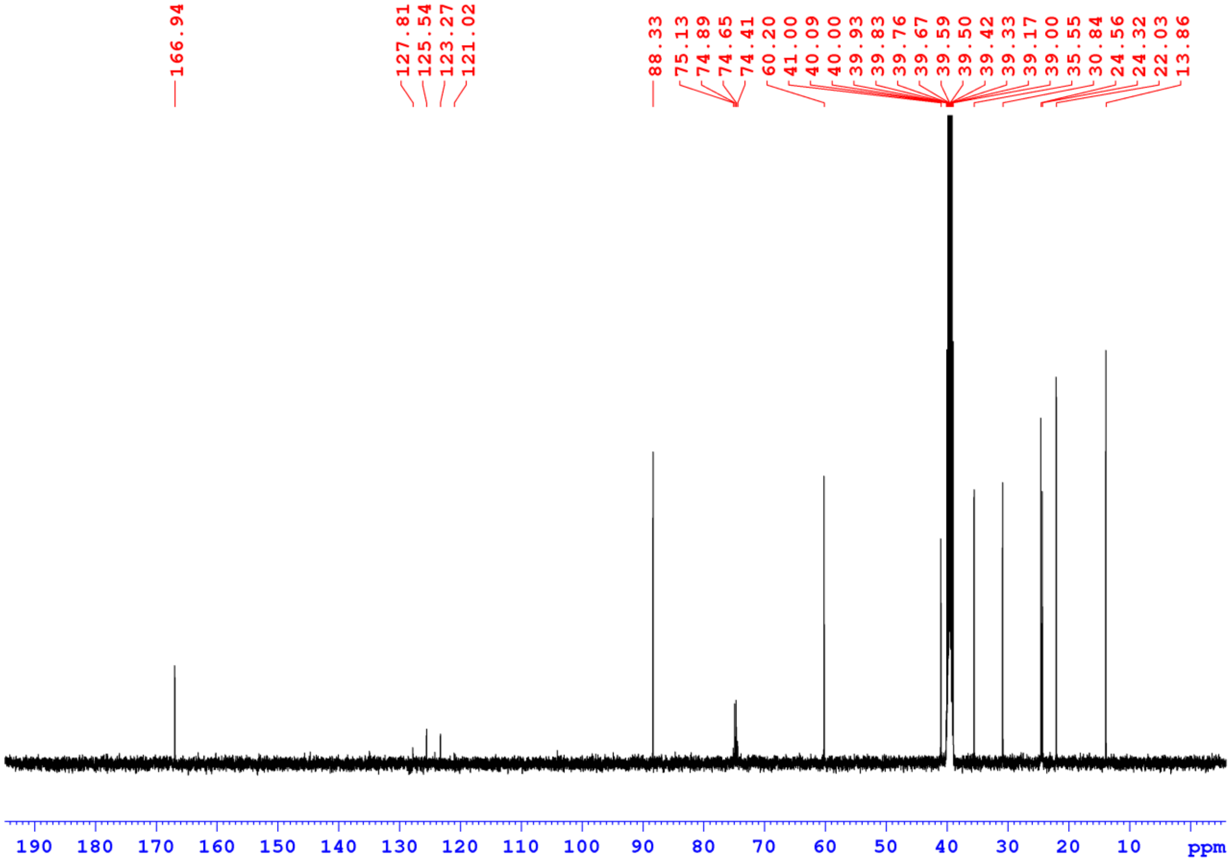

Figure S46. ^{13}C NMR spectrum of **8c**^{c}.

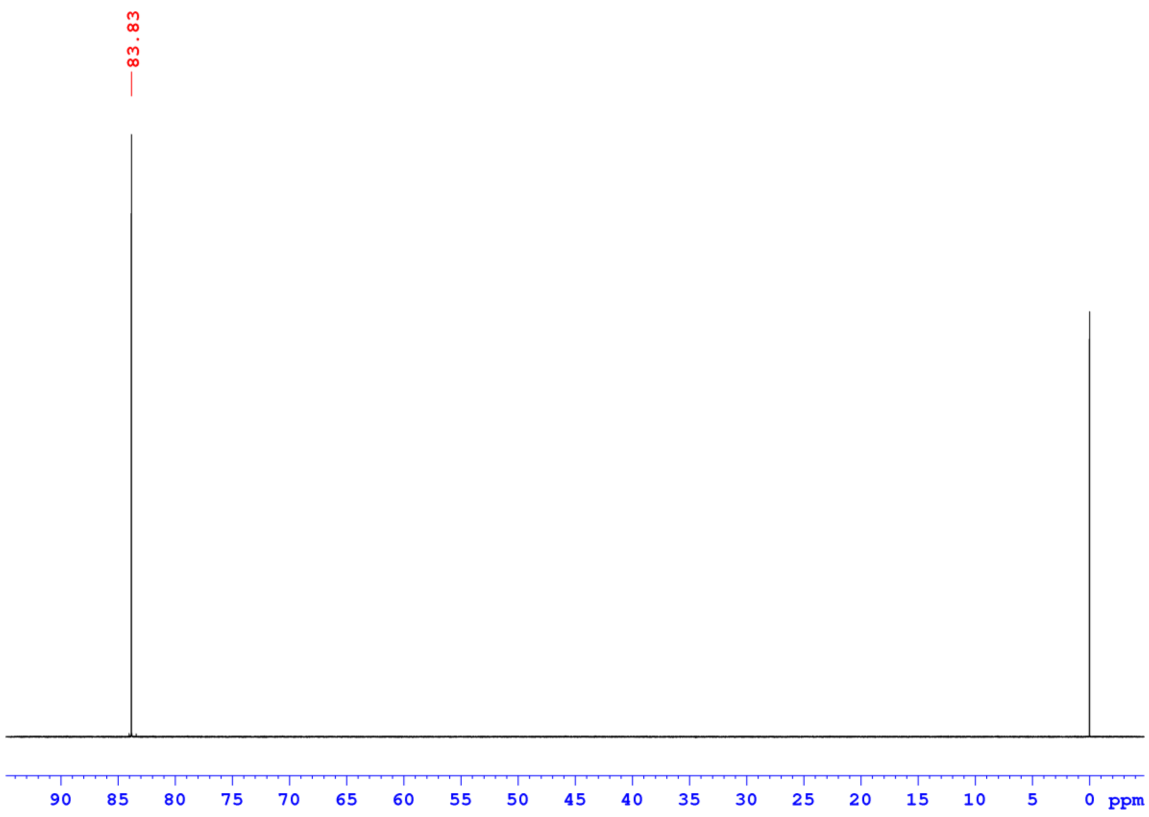

Figure S47. ^{19}F NMR spectrum of **8c**^{c}.

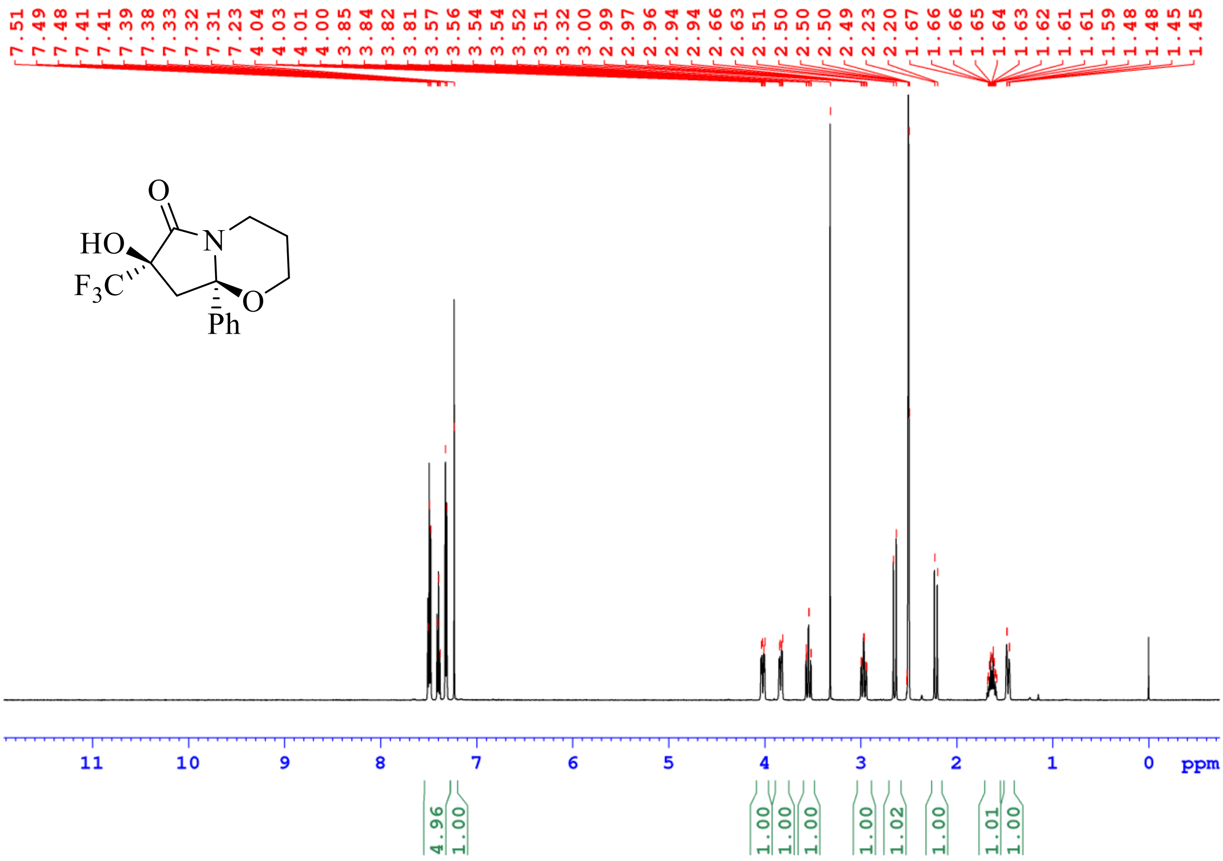

Figure S48. ^{1}H NMR spectrum of **8d**^{t}.

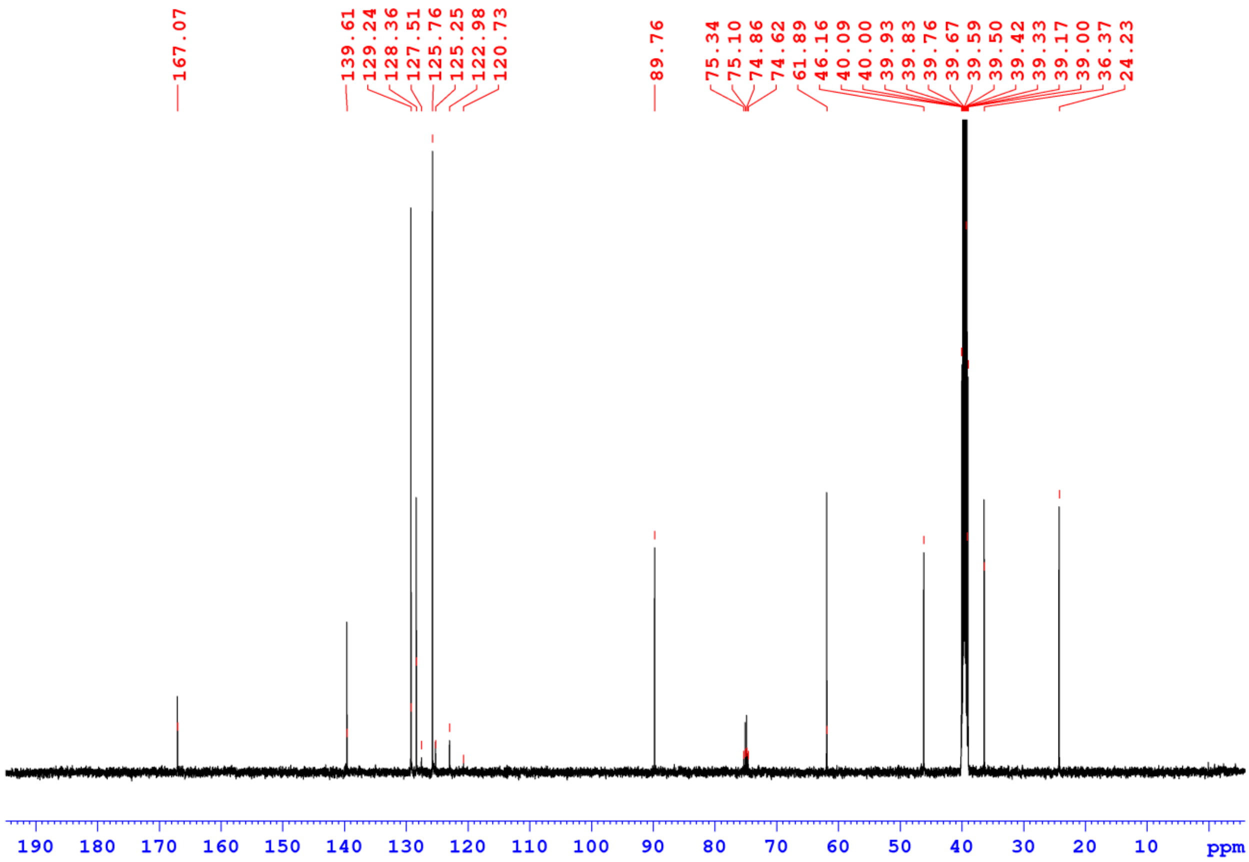

Figure S49. ^{13}C NMR spectrum of **8d**$^{\mathbf{t}}$.

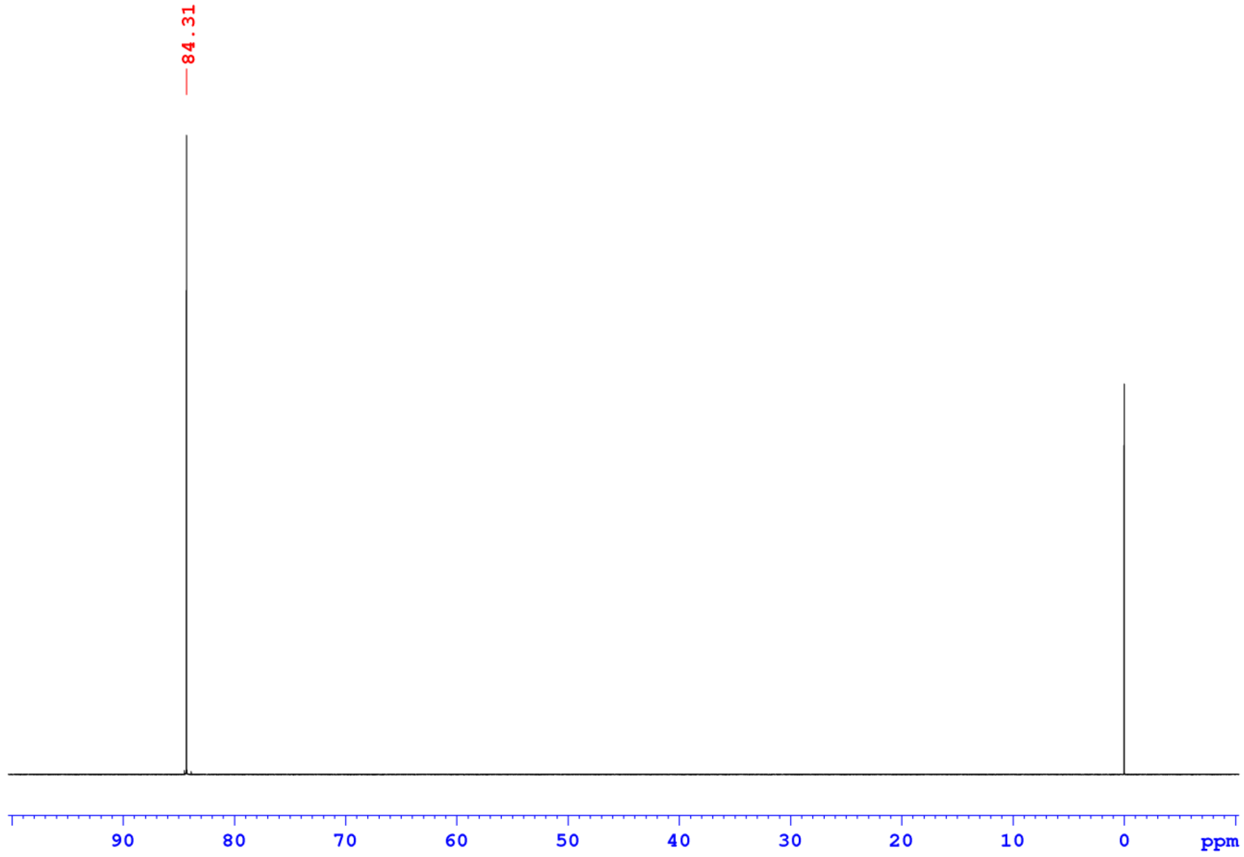

Figure S50. ^{19}F NMR spectrum of **8d**$^{\mathbf{t}}$.

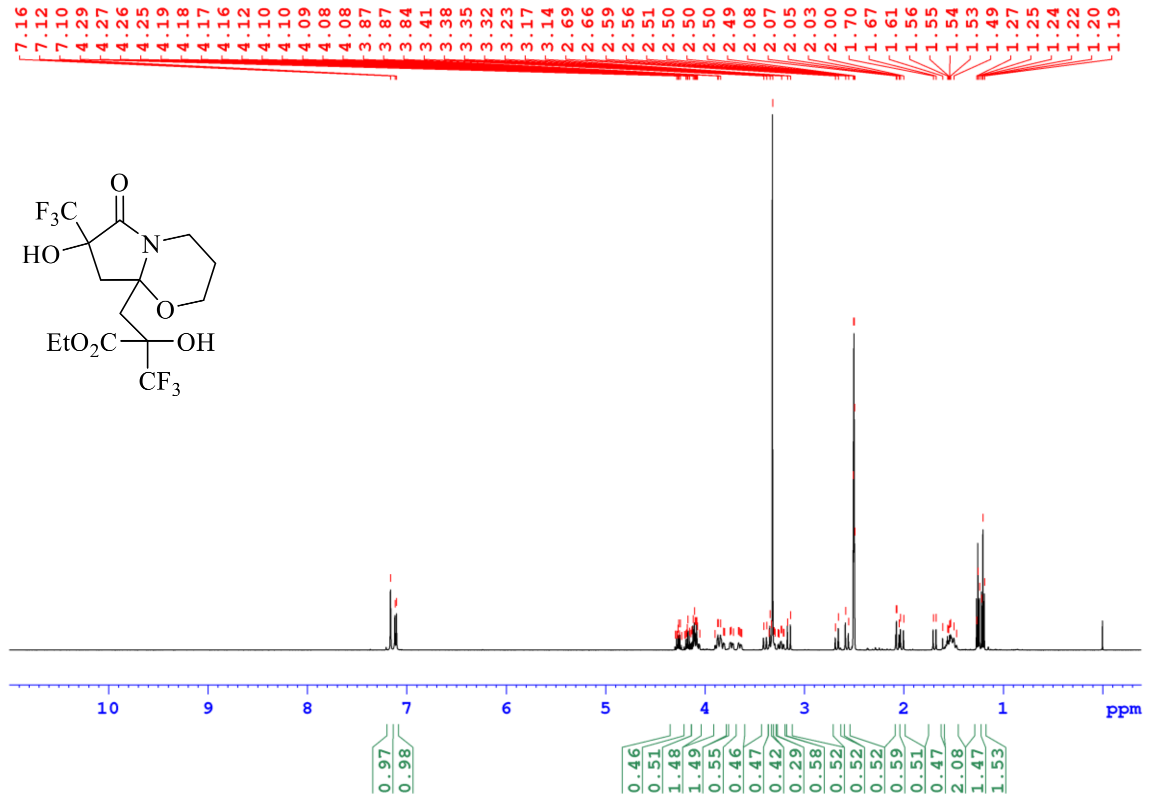

Figure S51. ^{1}H NMR spectrum of a mixture of **9** : **9'** in the ≈ 1 : 1.

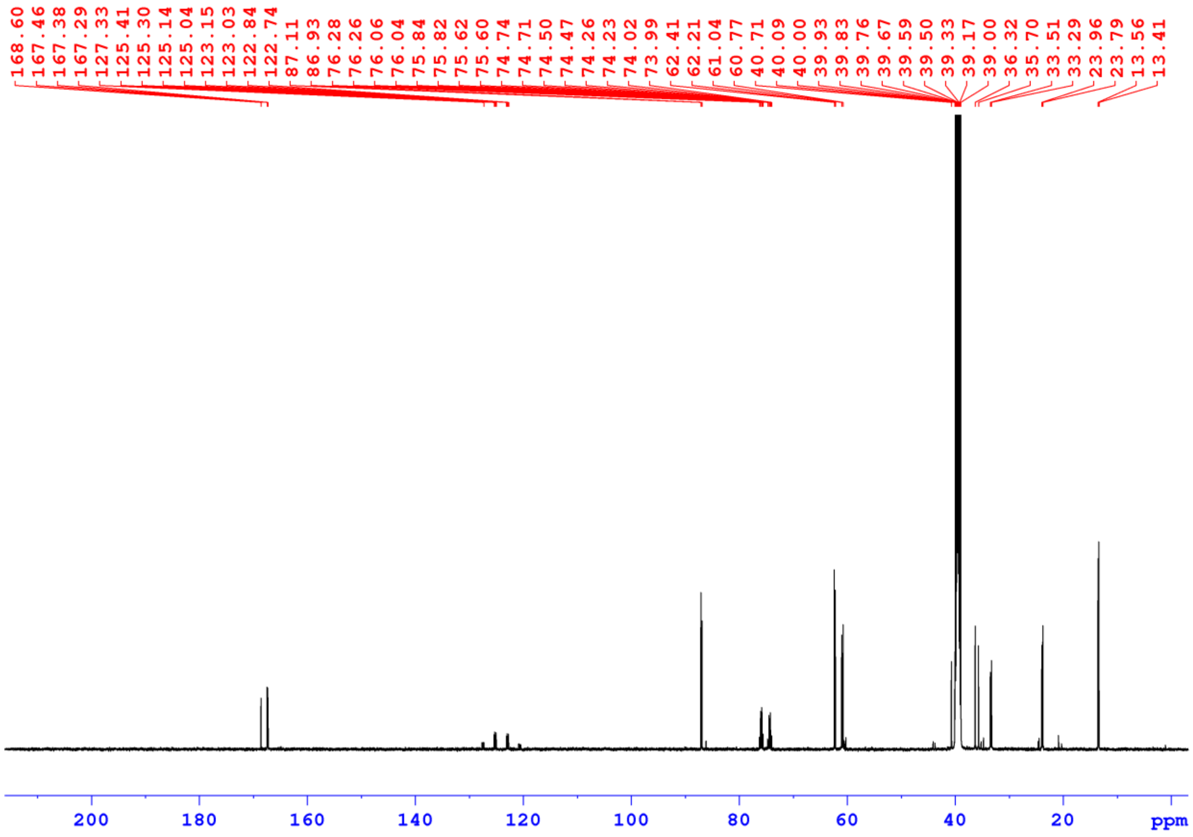

Figure S52. ^{13}C NMR spectrum of a mixture of **9** : **9'** in the ratio ≈ 1 : 1.

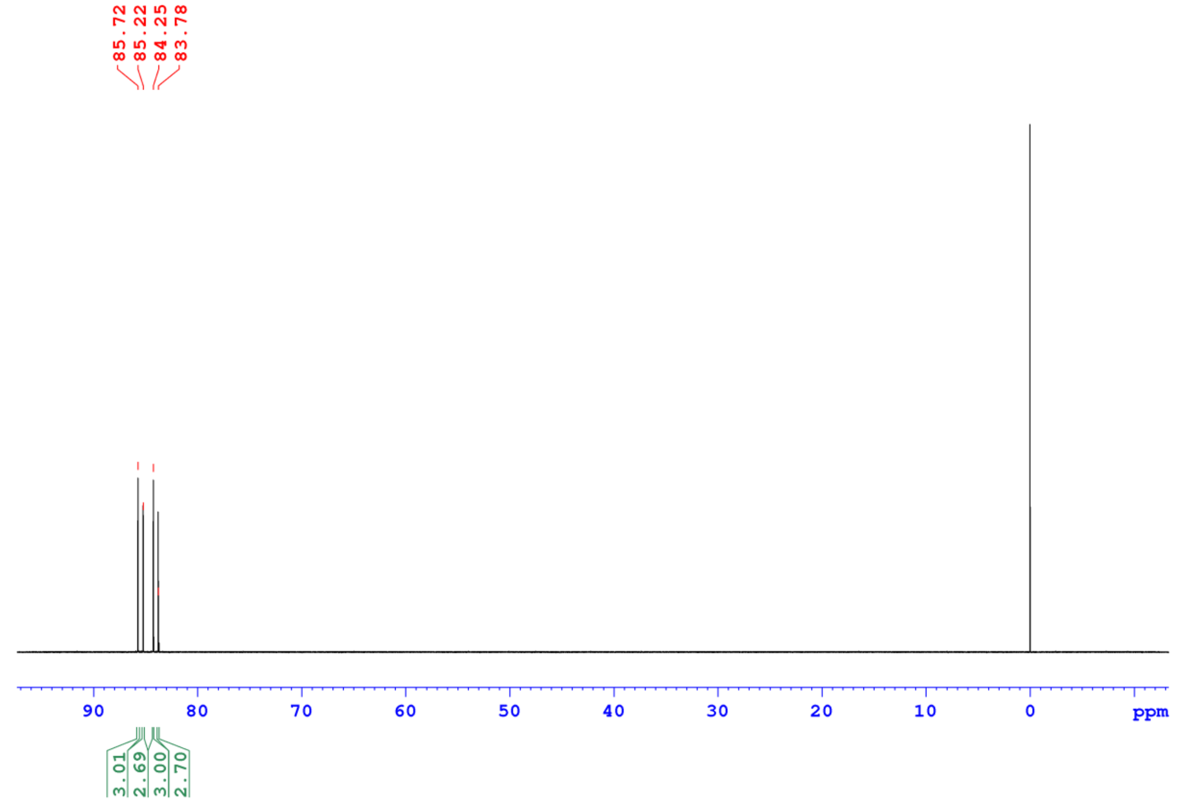

Figure S53. ^{19}F NMR spectrum of mixture of **9** : **9'** in the ratio ≈ 1 : 1.

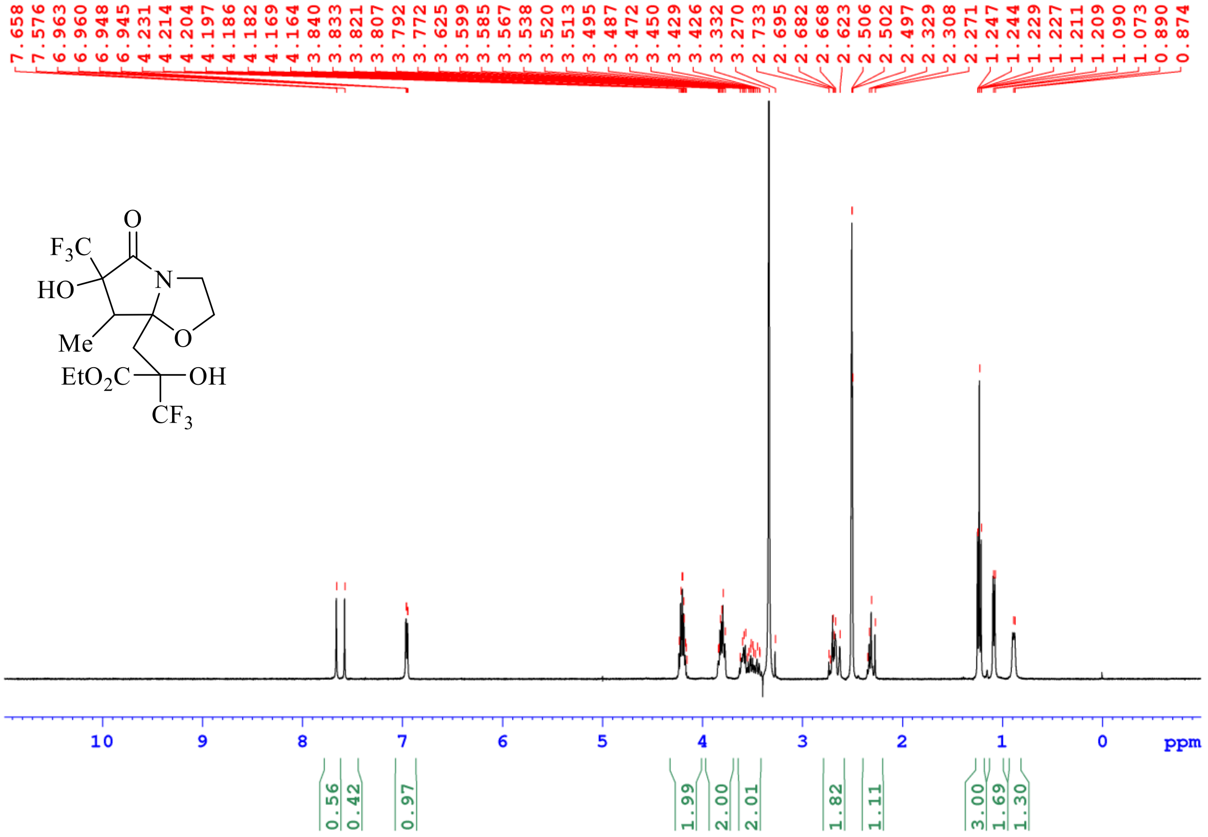

Figure S54. ^{1}H NMR spectrum of mixture of **10** : **10'** in the ratio 56 : 44.

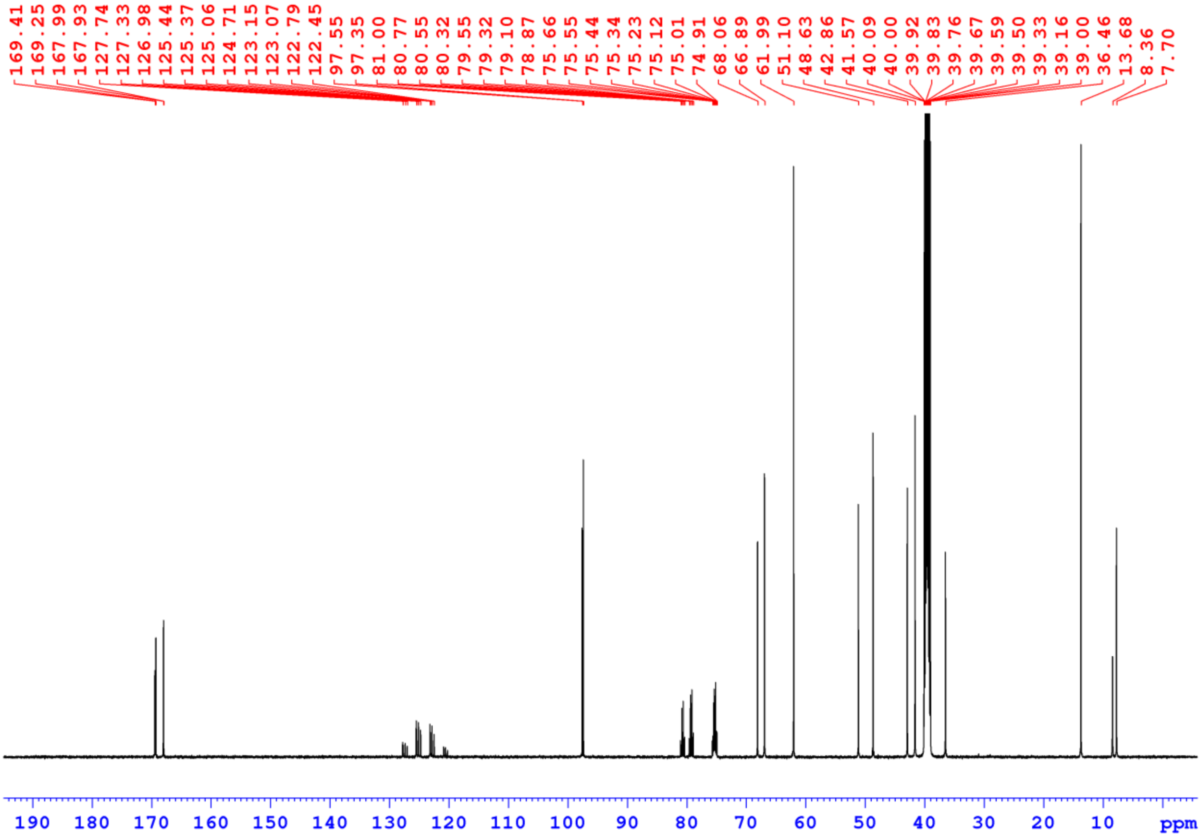

Figure S55. ^{13}C NMR spectrum of mixture of **10** : **10'** in the ratio 56 : 44.

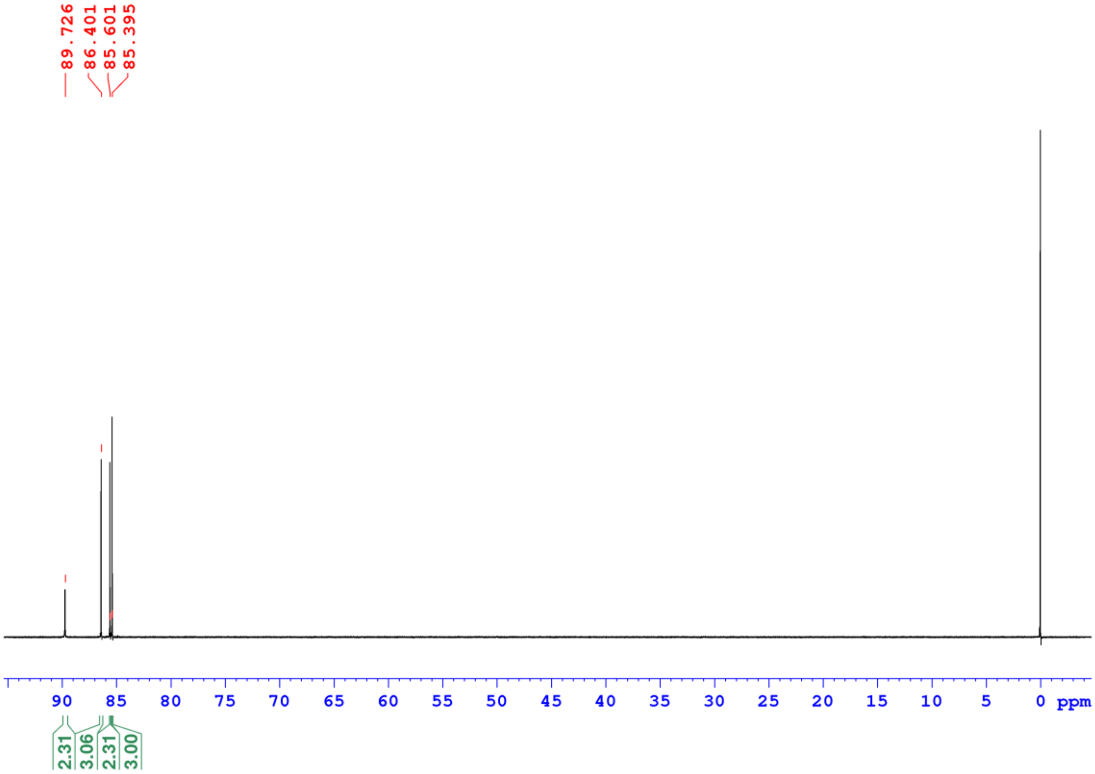

Figure S56. ^{19}F NMR spectrum of mixture of **10** : **10'** in the ratio 56 : 44.

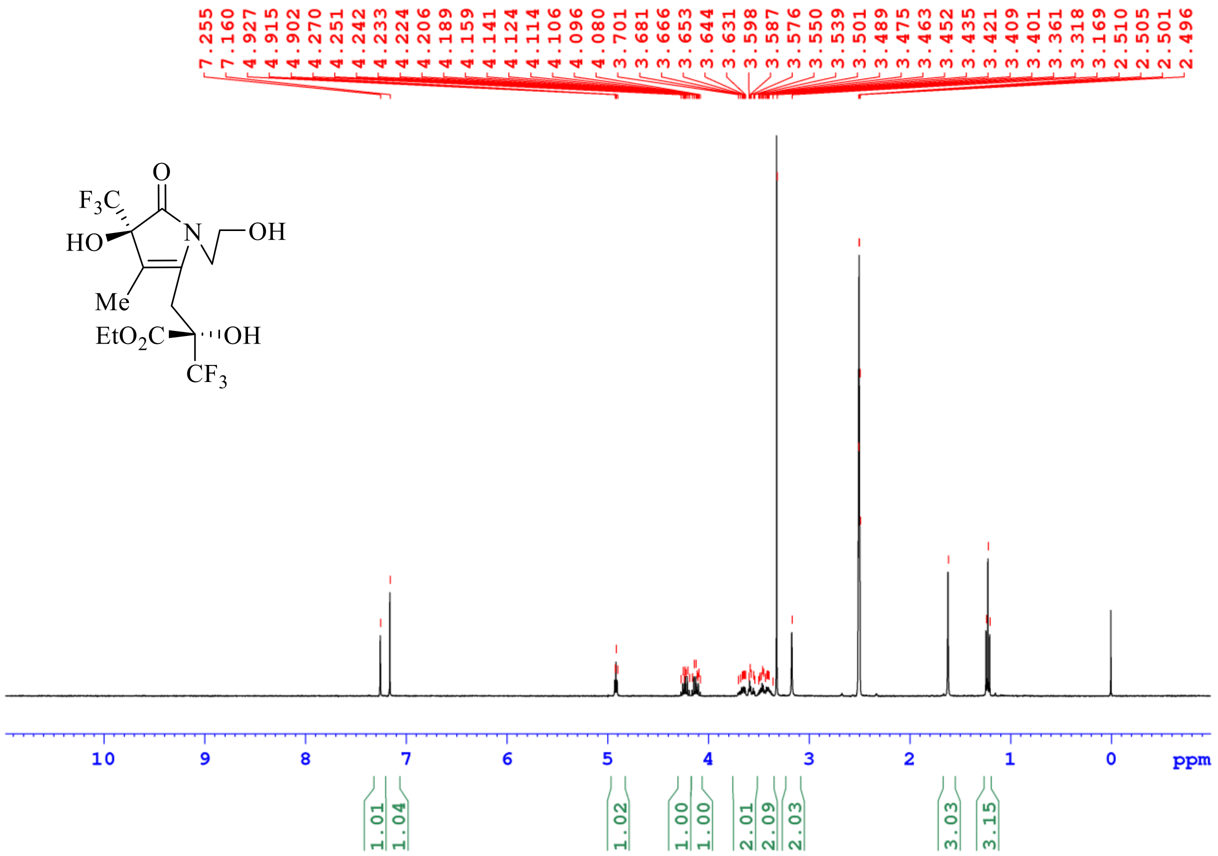

Figure S57. ^{1}H NMR spectrum of mixture of **11a.**

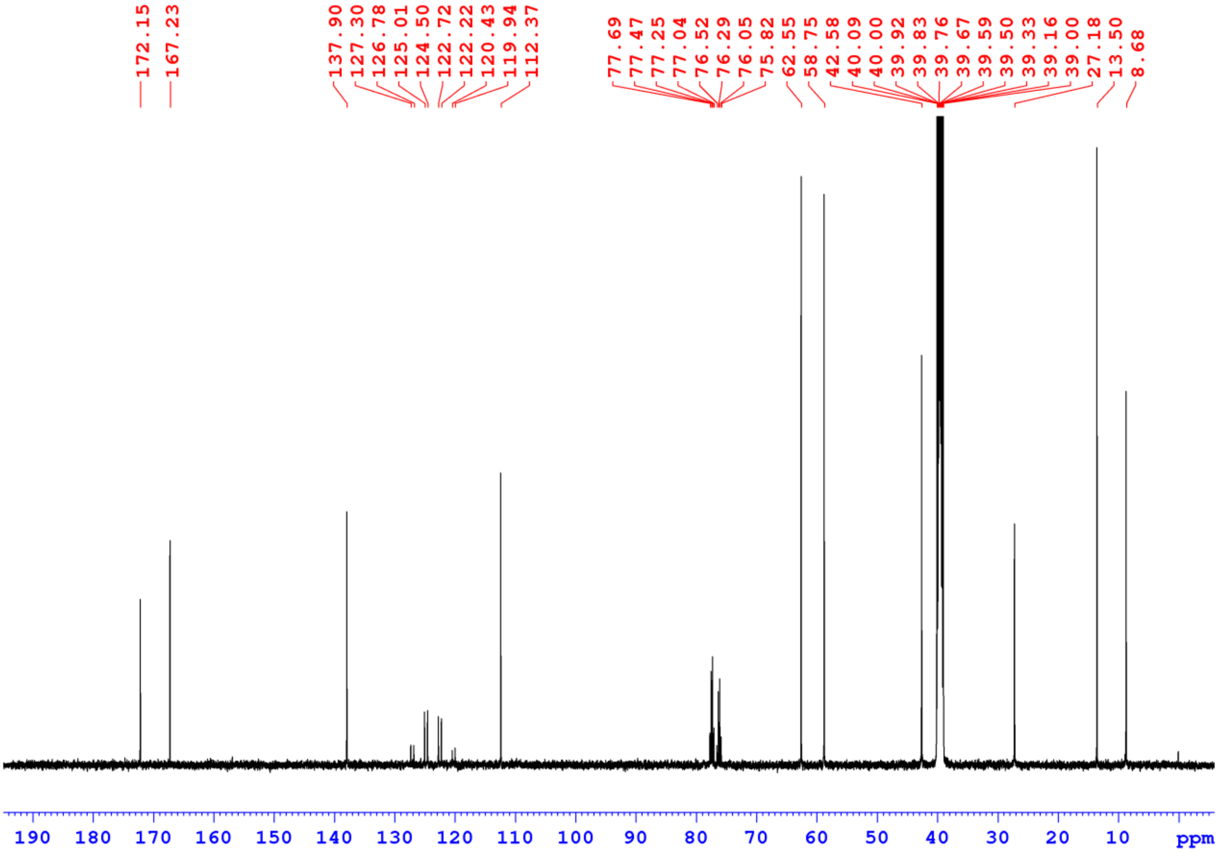

Figure S58. ^{13}C NMR spectrum of mixture of **11a.**

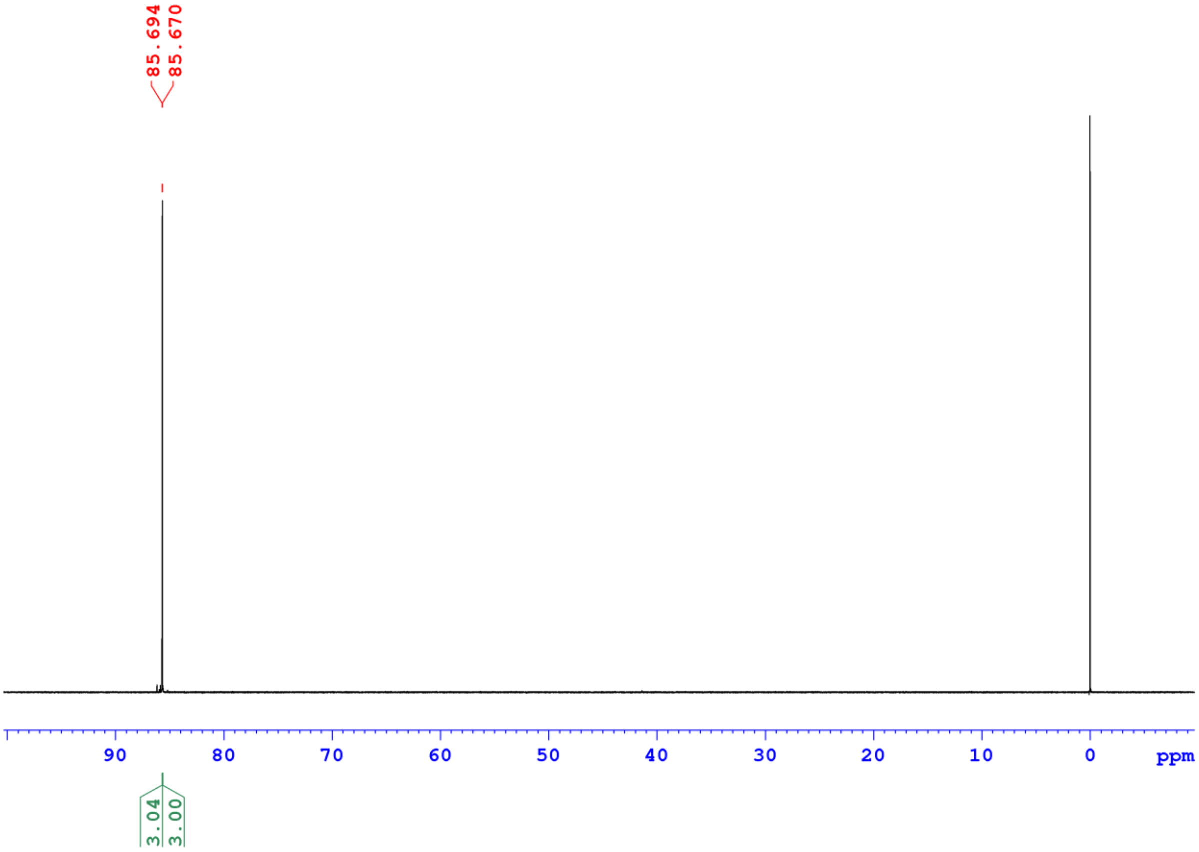

Figure S59. ^{19}F NMR spectrum of mixture of **11a.**

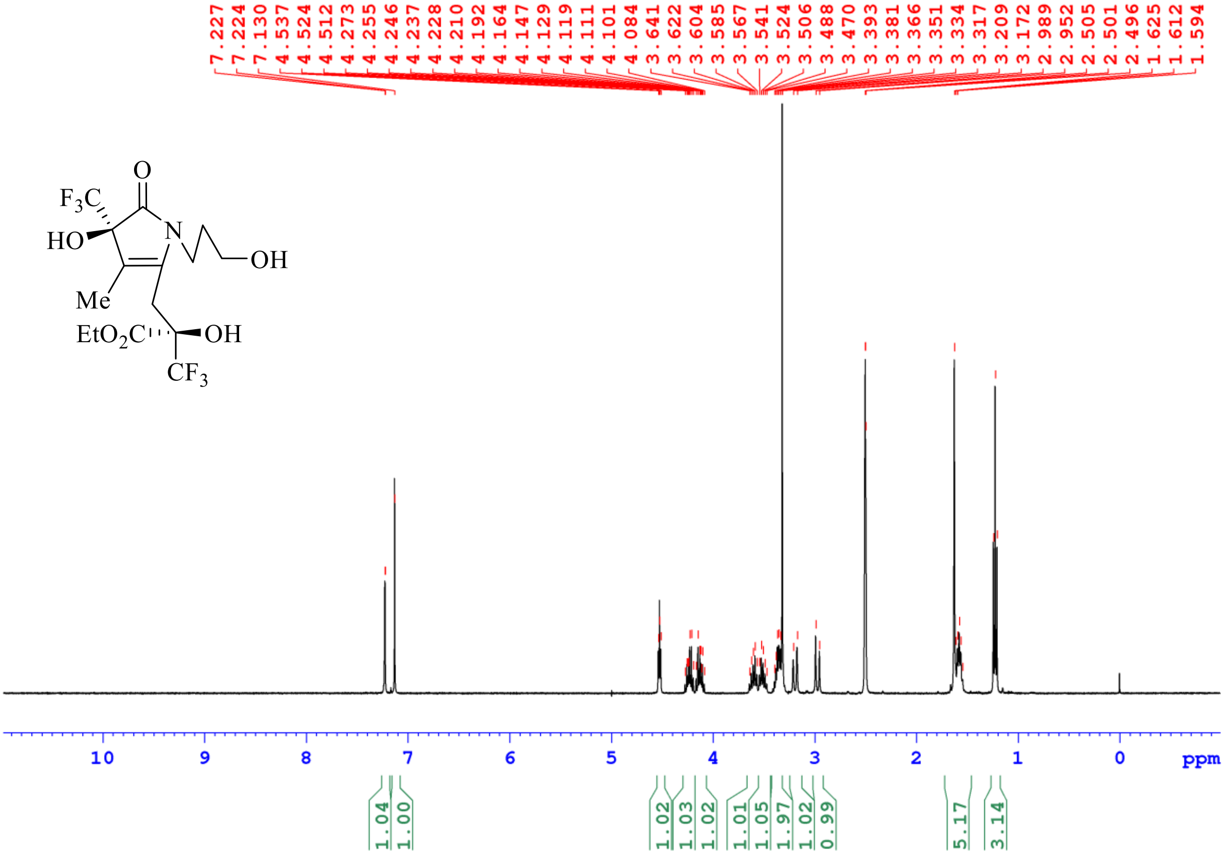

Figure S60. ^{1}H NMR spectrum of mixture of **11b.**

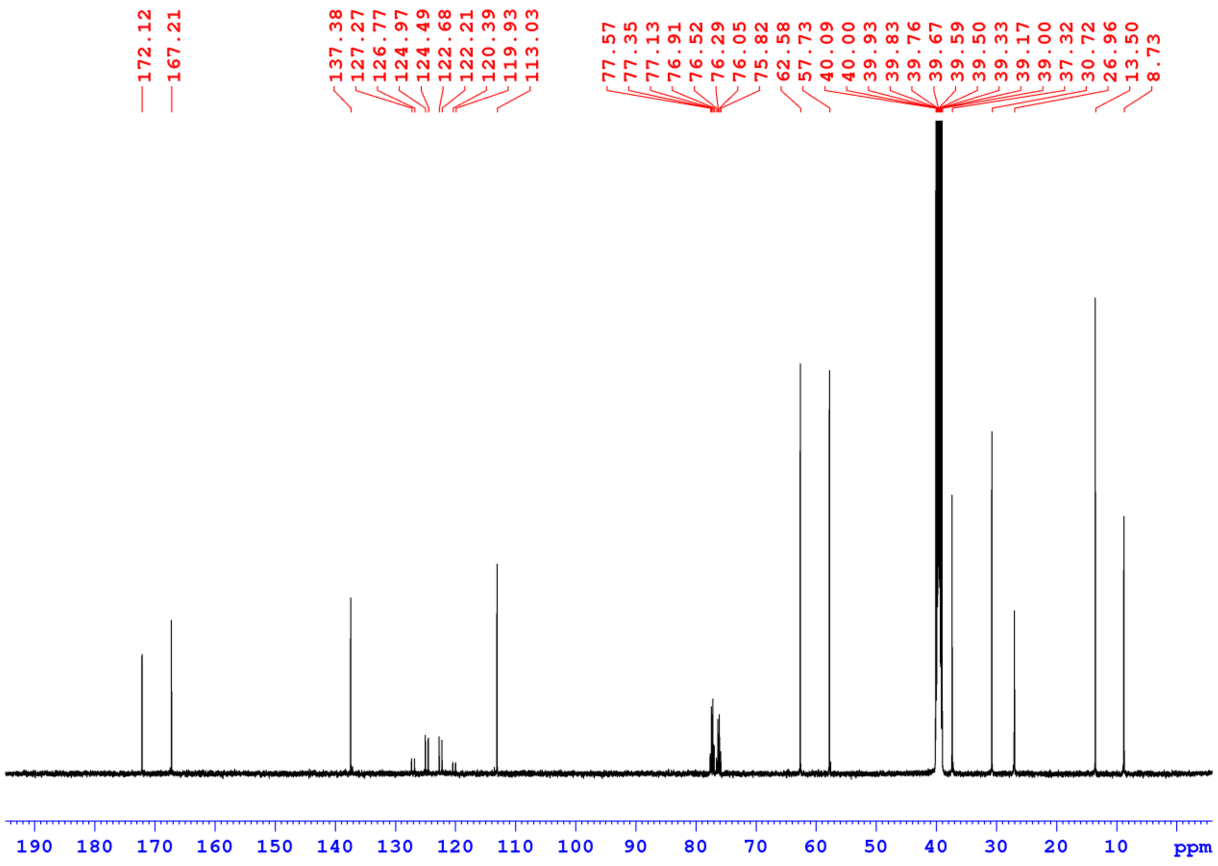

Figure S61. ^{13}C NMR (126 MHz, DMSO-d_6) spectrum of mixture of **11b.**

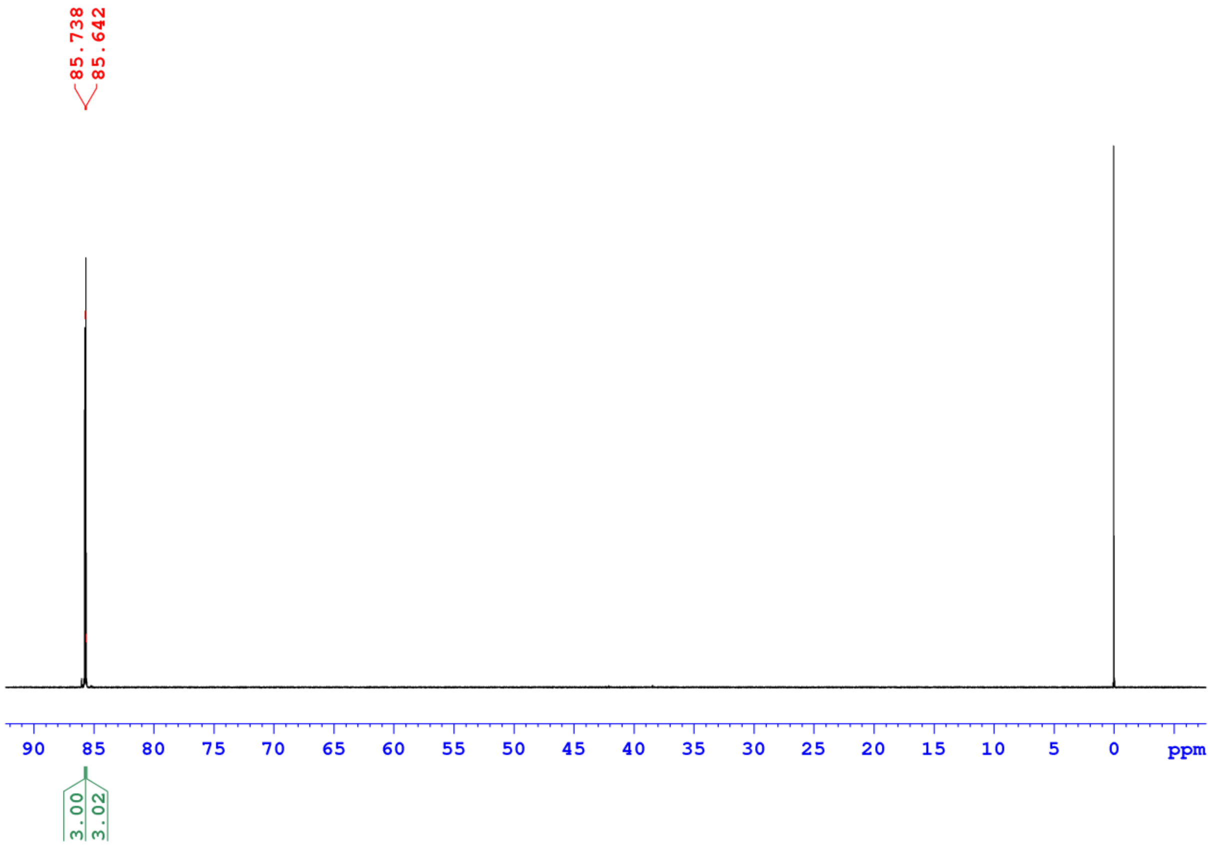

Figure S62. ^{19}F NMR spectrum of mixture of **11b.**

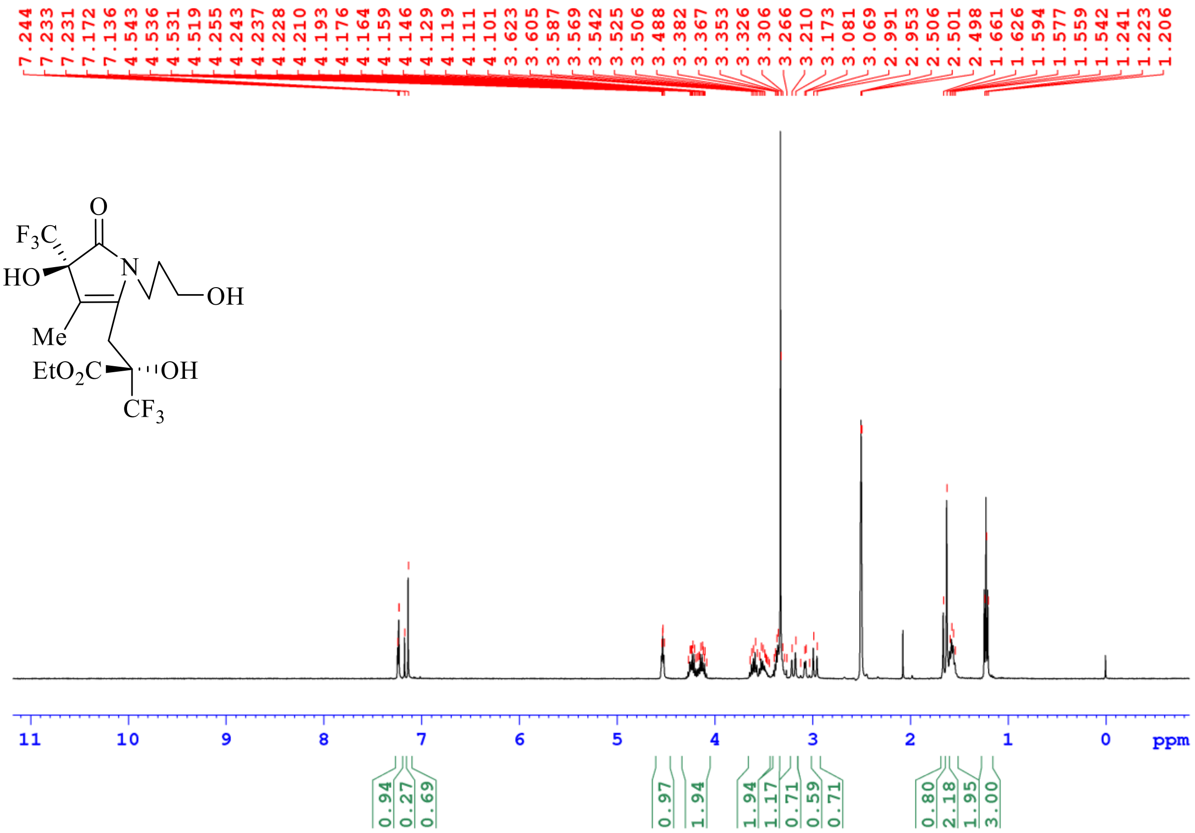

Figure S63. ^{1}H NMR spectrum of mixture of **11b : 11b'** in the ratio 72 : 28.

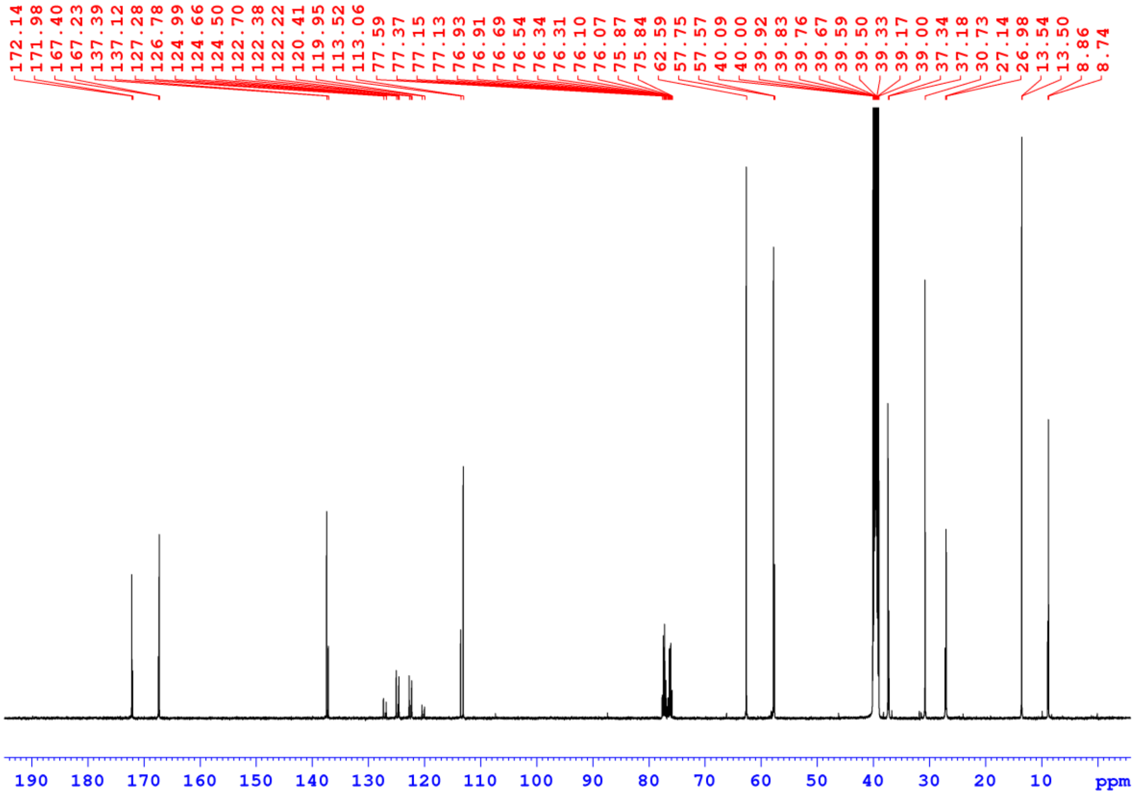

Figure S64. ^{13}C NMR spectrum of mixture of **11** : **11'** in the ratio 72 : 28**.**

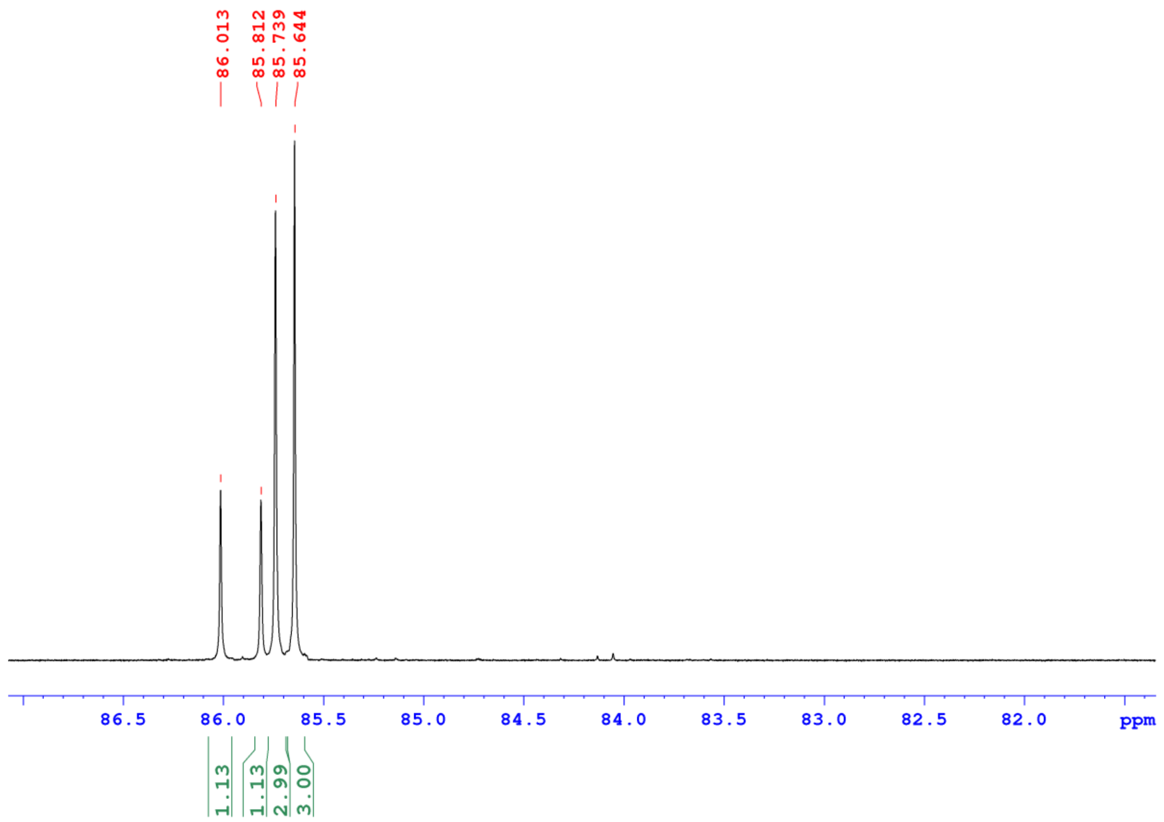

Figure S65. ^{19}F NMR spectrum of mixture of **11** : **11'** in the ratio 72 : 28**.**
